# Preparation and Characterization of Carvacrol-Loaded PLA Nanofibers by the Solution Blow-Spinning Method for the Long Shelf-Life of Chicken Breast Meat

**DOI:** 10.1021/acsomega.5c02604

**Published:** 2026-01-23

**Authors:** Tuğba Güngör Ertuğral, Yalçın Coşkun, Mine Çardak, Simge Özalp, Oğuz Kaan Coşkun, Aren Gürler

**Affiliations:** † Çanakkale Faculty of Applied Sciences, Department of Food Technology, 52950 Çanakkale Onsekiz Mart University, Çanakkale 17100, Turkey; ‡ Lapseki Vocational School, Department of Plant Production and Animal Husbandry, 52950 Çanakkale Onsekiz Mart University, Çanakkale 17100, Turkey; § Canakkale Faculty of Applied Sciences, Department of Fishery Technology, 52950 Çanakkale Onsekiz Mart University, Çanakkale 17100, Turkey; ∥ TÜBİTAK, Ankara 06420, Turkey; ⊥ TÜBİTAK, Ankara 06420, Turkey; # TÜBİTAK, Ankara 06420, Turkey

## Abstract

Using natural active packaging materials in food preservation is a healthy and safe method. Chicken meat is one of the foods in which microorganisms grow rapidly and so has a short shelf life. Extending the shelf life of food is also important for the economy. Packaging with biodegradable antimicrobial materials can reduce the rate of microorganism growth. In material studies on antimicrobial essential oils, carvacrol is generally used, and it is one of the most effective antimicrobial compounds of the Origanum onites species. Polylactic acid (PLA) is a biodegradable, natural polymer and a food-compatible biopolymer that is economical to produce. In this study, PLA nanofibers loaded with carvacrol (PLA/C) at 10, 20, and 30% (v/w) were produced by the solution blow-spinning method (SBS), and their antibacterial effects against *Escherichia coli* (*E. coli*) and *Staphylococcus aureus* (*S. aureus*) were measured by the disk diffusion method. Chicken breast meat samples packaged with PLA/C nanofibers were stored at +4 °C for 7 days, and the increase in total aero-mesophilic bacteria values was determined as 5 × 10^5^, 5 × 10^4^, 5 × 10^3^, and 4 × 10^3^ cfu/g for PLA/C, respectively. All processes were analyzed in triplicate, and the consistency of the results was tested at 5%. PLA/C nanofiber was characterized by scanning electron microscopy (SEM), Fourier transform infrared spectroscopy, and thermogravimetric analysis (TGA). Nanofiber diameters in SEM images were between 1.01 and 3.21 μm. According to TGA data, the nanofiber showed degradation at 378.21 °C.

## Graphical abstract

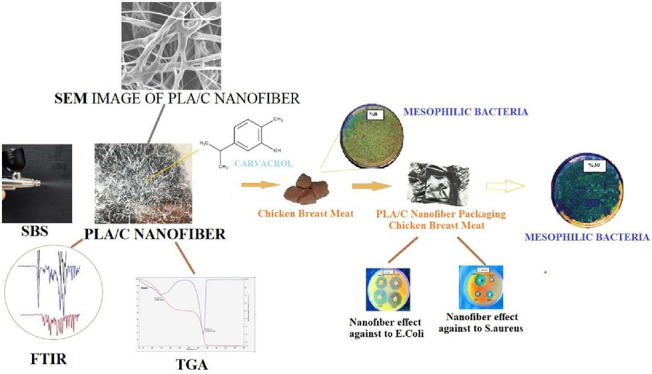

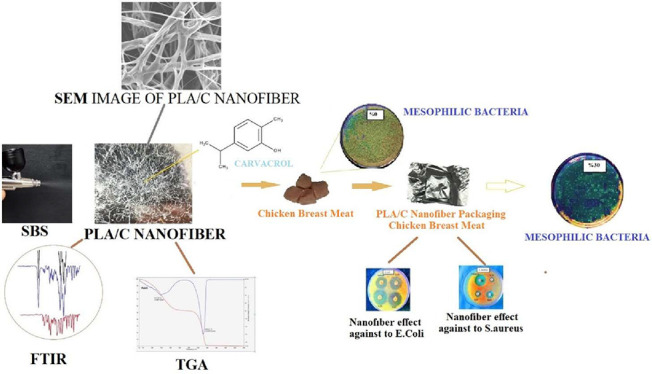

## 1 Introduction

Increasing food demand due to the world population brings food safety and quality to the forefront. In order to protect food and prevent the disposal of expired foods, interest in functional natural packaging materials that increase the shelf life has increased. However, plastic-based packaging used for food preservation causes serious environmental pollution and poses a threat to human health.^1^ On the other hand, using additives in food preservation may not be economical in terms of health and modified atmosphere packaging. This situation has increased the importance given to the development of biopolymer packaging systems that are biodegradable and do not cause any harm on contact with food, and biopolymers are versatile biodegradable materials obtained from natural sources.^2,3^ Especially in recent years, interest in nanofiber studies loaded with antimicrobial bioactive compounds against microbial spoilage in food products has increased, and these nanofibers are generally produced by the electrospinning (ES) method.^4^ The most consumed animal food in the world as a protein source is chicken meat, at 122 million tons.^5^ Fresh chicken meat is at risk of microbial spoilage during storage and transportation due to factors such as nutrient content, water activity, and temperature. To prevent this situation, modified atmosphere packaging^6^ or frozen preservation, which are uneconomical, are preferred,^7^ and chicken carcass meat can maintain its quality only for about 3 days at 4 °C.^8^

Alternatively, food preservation and shelf-life studies with antimicrobial nanofibers^9^ and especially nanomaterials containing antimicrobial essential oils can provide active protection in food preservation.^10^ Herbal essential oils have biological activities including antibacterial, antifungal, antiviral, and antioxidant.^11^ Also, thyme (Thymus genus) species have strong disease-preventing properties.^12–15^ Azaz et al. (2004) reported that thymol, carvacrol, and borneol were the major components in the essential oils of *Thymus longicaulis* and *Thymus zygioides* species.^7,16^ The presence of various antimicrobial compounds in essential oils and the biodegradable films activated by these agents are effective in minimizing the growth potential of microorganisms, ensuring food safety and increasing the shelf life of food.^17^ Thymol and carvacrol, the major components of thyme essential oil, are known to be effective against pathogenic bacteria,^18,16^ viruses,^19^ fungi,^20,21^ and parasites.^22^ Nanoscale containers and fibrous materials may have applications not only in packaging but also in plant protection, agriculture, food logistics, and the food chain including storage, and this could be an effective application for thymol and carvacrol.^23,15^ Previous studies have also indicated that carvacrol exhibits antimicrobial effects in soy protein, starch, and polyester-based packaging materials.^24,25^ On the other hand, polylactic acid (PLA), serves as a polymer matrix to produce active food packaging films with the addition of natural extracts or essential oils from plants.^26–28^ One of the main characteristics of the PLA matrix is its ease of degradation by enzymatic^29,30^ or hydrolytic means.^31^ PLA is Food and Drug Administration approved, noncytotoxic, and compatible with living metabolism.^32^ Petroleum-based plastics used for food packaging have some disadvantages such as decreasing oil and gas resources, increasing crude oil prices, and environmental concerns and global warming due to its combustion.^33^ At the same time, essential oils and phase change materials can be loaded into PLA-based polymer matrices.^34^

The production of environmentally friendly, biodegradable, and antimicrobial nanofiber materials has been the focus of attention in recent years. The most commonly used method in nanofiber production is the the ES method, but it operates at high voltage of 14 or 20 kV rms AC in protected cabins. The ES system poses occupational health and safety risks in these aspects and is an expensive system.^35,36^

On the other hand, different researchers are conducting nanofiber studies with the solution blow-spinning method (SBS), which was first reported by Medeiros et al. in 2009.^37^ The SBS method is not applied at high voltage and is based on the principle of passing a polymer solution through a fine-tipped nozzle with a pump providing air pressure. In the ES system, nanofibers are produced only in the laboratory and in a fixed location inside the cabin. The SBS method is safe and also allows the production of nanofibers at the desired location. It can provide fast and easy production.^38^

In the SBS method, compressed air/gas replaces electrical forces, and the production speed is 30 times higher than that of ES.^39^ This method provides nanofiber formation by thinning the polymer solution from the inner channel and the polymer from the spinneret tip with pneumatic jet force effect from the outer channel.^38–40^

Active packaging system studies have been conducted on chicken meat in food preservation. Liu et al. (2022) developed a food packaging antibacterial hydrogel based on gelatin, chitosan, and 3-phenyllactic acid to extend the shelf life of chilled chicken.^41^ Higueras et al. (2014) also determined the antimicrobial effect depending on the storage period and film dimensions in the study where they obtained the films by dipping them in carvacrol for 3 weeks to reach the equilibrium in glycerol-plasticized chitosan: hydroxypropyl-β-cyclodextrin films^42^ and, also, agar/konjac glucomannan films combined with 2% carvacrol can extend the shelf life of chilled chicken breast meat from 5 to 9 days.^43^ A chitosan film containing 2% thyme oil reduces bacterial load in cold storage for more than a week; on the other hand, PLA nanofibers loaded with perilla essential oil can extend the shelf life of chicken meat by 12 days.^44^ Curcumin-loaded polycaprolactone/carboxymethyl chitosan nanofibers capable of photodynamic inactivation of *S. aureus* were produced using the SBS method.^45^ In addition, gelatin/zein/polyurethane complex nanofibers were obtained using the SBS method for application in food antimicrobial packaging.^46^ Volatile oil obtained from the sausage spice mixture was incorporated into PLA, and nanofibers were produced via SBS; their antibacterial properties were tested against *E. coli* and *S. aureus*.^47^ The applicability of poly­(vinyl alcohol)-based nanofibers containing various extracts produced using the SBS method for strawberry preservation has been proven in increasing the shelf life of strawberries.^48^ Chicken meat pieces were coated with nanofibers produced by the ES method coated with eugenol-loaded nanofibers, and a decrease in bacterial growth was observed for 7 days.^49^ In a different product such as chicken soup, thyme essential oil/β-cyclodextrin ε-polylysine nanoparticles showed antimicrobial activity.^50^ On the other hand, chitosan-based poly­(ethylene oxide) containing nanofibers showed antibacterial activity against bacteria that frequently play a role in food contamination and spoilage such as *Escherichia coli*, *Salmonella enterica* serovar Typhimurium, *Staphylococcus aureus*, and *Listeria innocua*.^51^ In this study, which aims to select carvacrol as an effective antimicrobial agent and load onto natural and biodegradable PLA and apply it as an active packaging in foods, PLA nanofibers loaded with carvacrol at different percentages were produced by the SBS method, and nanofibers were characterized, the antibacterial effect against *E. coli* and *S. aureus* in chicken breast meat coated with PLA/C nanofibers was tested, and total aero-mesophilic bacteria (TAMB) test development after cold storage was investigated.

## 2 Materials and Methods

### 2.1 Materials

PLA was purchased from Natureworks LLC (4043 D Nebraska, USA) (*M*_n_ = 160,000 g/mol). Dichloromethane (DCM) 99% (purity) (Merck EMSURE) was used as a solvent, and carvacrol was purchased from Sigma-Aldrich brand, CAS no: 499-75-2, natural, 99%, FG.

### 2.2 Preparation of PLA/C Nanofibers by the SBS Method

Nanofibers were prepared by modifying the formulation of Zhang et al. 2020 and Güngör Ertuğral 2024. For this purpose, 0.5 g of PLA was dissolved in 10 mL of DCM solution at room temperature for 24 h;^52,44^ then, carvacrol was added to this solution and mixed again for 10 min. This process was applied separately for each of the nanofibers containing 10, 20, and 30% (v/w) carvacrol.^43^ Then, 8 mL was placed in the portable air injector (thin needle atomization spray; Sky-4-automatic) compartment. Nosel with an inner diameter of 0.3 mm and 0.5 mm outer diameter was sprayed on the aluminum foil collector at a distance of 17 cm at a pressure of 0.3 MPa.^52^

### 2.3 Characterization of Nanofibers

#### 2.3.1 FTIR Spectroscopy Analysis

Fourier transform infrared (FTIR) spectroscopy analyses of PLA/C nanofibers were carried out with a 100 FTIR spectroscopy spectrum spectrometer in transmission mode with a resolution of 4 cm^–1^ and a wavelength scanning range of 4000–650 cm^–1^.^53^

#### 2.3.2 Thermogravimetric Analysis

The thermal degradation behavior of PLA/C nanofibers was measured by thermogravimetric analysis (TGA; SDT Q600 V20.9 Build 20) between 0 and 650 °C at a heating rate of 10 °C/min in a nitrogen atmosphere.^54^

#### 2.3.3 SEM Analysis

The morphology of nanofibers was examined by scanning electron microscopy energy-dispersive X-ray spectroscopy (SEM-EDX; JEOL JSM-7100-F, Tokyo, Japan) at an operating voltage of 15 kV. The conductivity was increased by coating the samples (Quarum Coated Device) with Au–Pd (80/20%).^40^

### 2.4 In Vitro Antibacterial Test

The disk diffusion method was applied to examine the antibacterial activity properties of 10, 20, and 30% (v/w) PLA/C nanofibers produced by the SBS method^55^ and tested against Gram-negative *E. coli* ATCC 25922 and Gram-positive *S. aureus* ATCC 25923 reference bacterial strains. 100 μL (0.5Mc Farland) of 0.5% Mueller–Hinton Agar (MHA) medium was used and nanofiber samples were prepared as standard disks with a diameter of 0.5 mm and placed in the medium with control samples (Vancomycin and Amikacin). Inhibition zones formed at the end of 18–24 h incubation period at 37 °C were measured in millimeters.

Chicken breast samples were wrapped in PLA/C nanofibers and stored at 4 °C in closed polypropylene containers to prevent moisture loss. The relative humidity during storage was maintained at 85–90% using moistened cotton pads. All samples were kept under dark conditions to avoid the photodegradation of carvacrol. The storage period lasted 7 days, with microbiological analysis performed on days 0 and 7.

### 2.5 TAMB Test

For the TAMB test, a 200 g chicken breast meat sample, chicken breast meat sold as “chicken tenderloin,” was randomly taken from markets in 500–1000 g packages and stored at +4 °C. Simultaneously, chicken meat samples were coated with 4 different nanofibers under aseptic conditions and then packaged in aluminum foil, a packaging material with low water vapor and oxygen permeability, and stored at +4 °C for 7 days (Scheme 1).

**Scheme 1:**
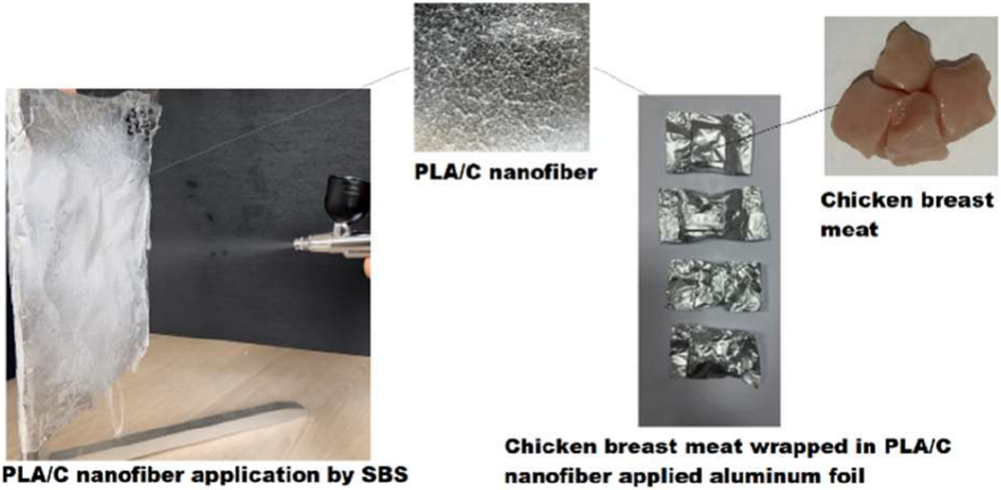
Schematic Representation of Nanofiber Preparation by the SBS Method and Chicken Breast Meat Application

TAMB counts for each sample were calculated according to the method of Harrigan (1998), and the samples were weighed in 10 g sterile plastic bags under aseptic conditions; 90 mL of 0.1% sterile peptone water was added, and a 10^–1^ dilution was prepared by homogenizing in a mixer (Interscience BagMixer 400 mL, France) for 2–3 min; then, decimal dilutions up to 10^–6^ were obtained from this dilution, and inoculation was performed.^56^ TAMB ISO 4833 (2003) counts were determined in chicken breast meat samples according to the methods reported by the International Organization for Standardization (ISO).

### 2.6 Statistical Analysis

Statistical Packages for the Social Sciences (SPSS) version 25 commercial software (IBM Corp.; Armonk, NY, USA) was used to analyze the data obtained in the study. Measurements were expressed as mean ± standard deviation. The normality of numerical variables was determined using the Kolmogorov–Smirnov test and kurtosis–skewness coefficients. Differences between measurements were compared with each other for *S. aureus* and *E. coli* using one-way analysis of variance (ANOVA). Following ANOVA, Tukey’s multiple comparison tests (Posthoc tests) were applied. The significance level for the comparison was set at *p* < 0.05.

## 3 Results and Discussion

### 3.1 FTIR Spectroscopy Analysis

Peaks between 3455 cm^–1^ are due to stretching vibrations of hydroxyl groups^57^ and PLA varlığı ile titerşim azalmıştır. In the FTIR spectroscopy spectrum of pure PLA (Figure 1), the asymmetric stretching of the methyl group at 2961 cm^–1^ increased in the PLA/C nanofiber according to the pure PLA stretching intensity. The symmetric −C–H stretching and the C=O stretching vibration of the carbonyl group from the repeating ester unit at 1755 cm^–1^ decreased according to the pure PLA stretching intensity. 1129–1361 cm^–1^ and −CH_3_ group at 1456 cm^–1^ were determined. Carvacrol shows characteristic peaks at 3371 cm^–1^ (OH), 2959 cm^–1^ (CH stretching), 1458, 1420, and 1301 cm^–1^ (CH), and 865 and 812 cm^–1^ (aromatic rings).^58^

**Figure 1:**
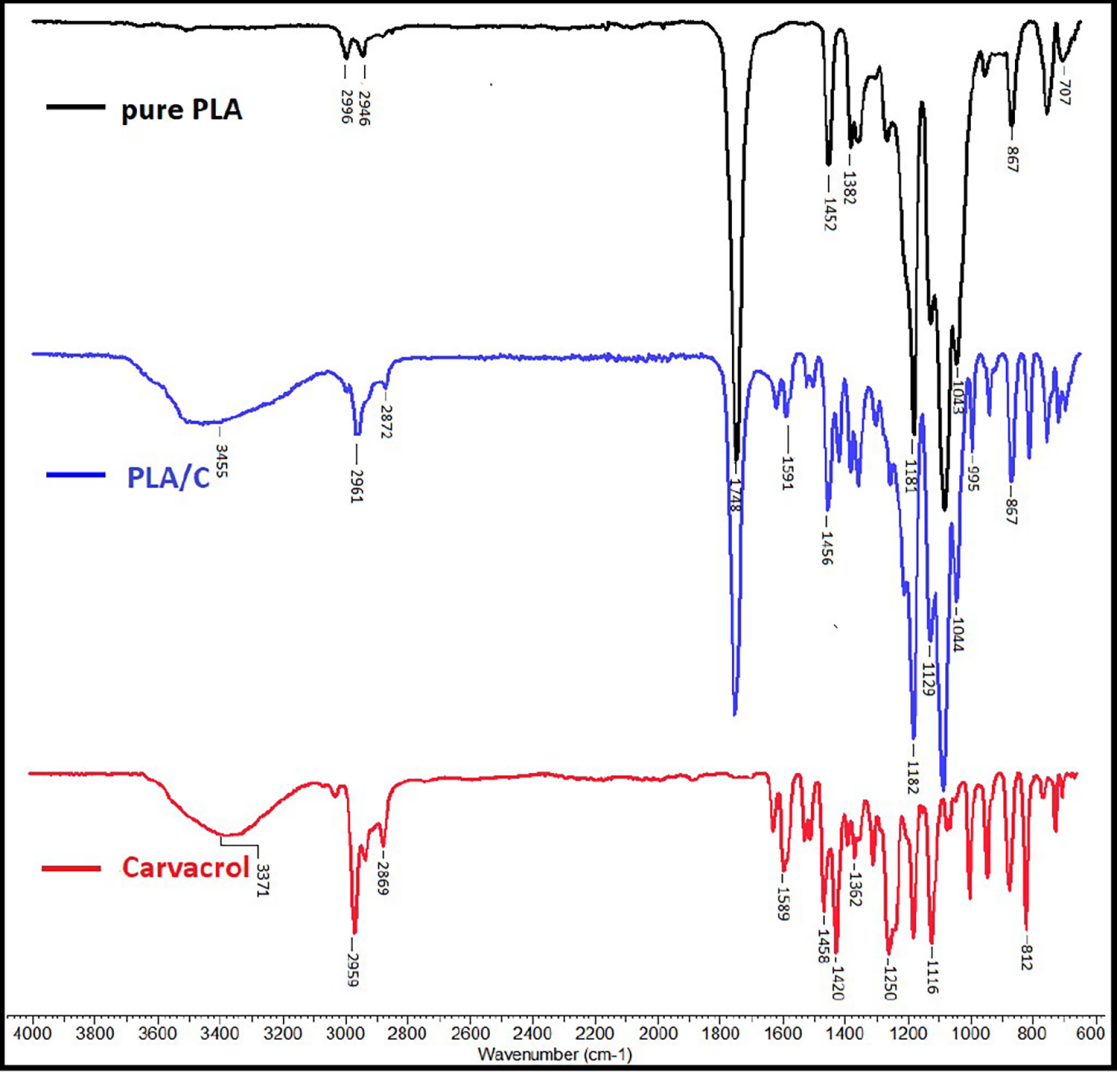
FTIR spectroscopy spectra of pure PLA nanofiber, PLA/C nanofiber, and carvacrol samples.

The distinct peak at 1452 cm^–1^ in the pure PLA nanofiber decreased in the PLA/C nanofiber, which may be due to the interaction.^59^ In addition, new peaks were formed between 1470 and 1690 cm^–1^ with the addition of carvacrol, and the tension intensity decreased, indicating that the decrease in the C–C tension intensity is due to interaction of carvacrol and PLA. The vibration of 1755 cm^–1^ belonging to the carbonyl group in the pure PLA nanofiber decreased. Stretching vibration changes in functional groups could be attributed to the presence of carvacrol (Figure 1).^60^

### 3.2 Thermogravimetric Analysis

The thermal degradation curves of pure PLA and PLA/C 30% (v/w) nanofibers were analyzed by TGA (Figure 2). The degradation temperature of the pure PLA nanofiber was approximately 348 °C, and it completely degraded at above 530 °C; PLA/C 30% (v/w) began to degrade at approximately 350 °C and completely degraded above 570 °C; PLA/C 30% (v/w) and carvacrol degraded at 147.79 °C.^61^ PLA/C 30% (v/w) and pure PLA nanofibers are close in value but the PLA/C 30% (v/w) nanofiber increases by approximately 5 °C compared to pure PLA.^37^

**Figure 2:**
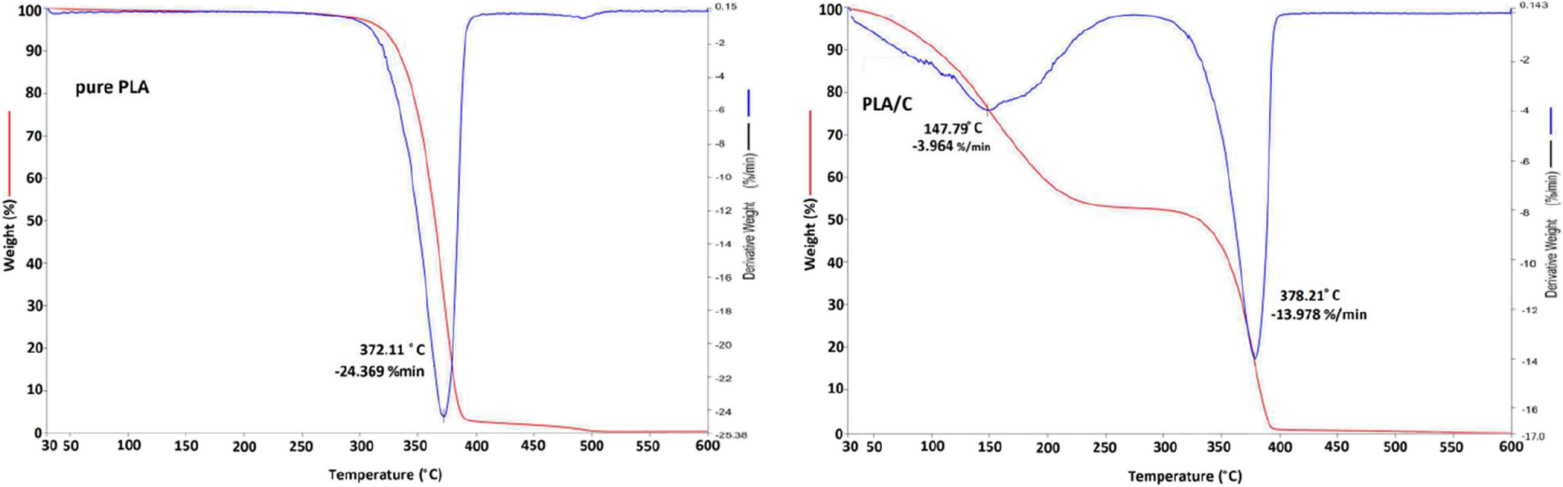
TGA curve of pure PLA and PLA/C 30% (v/w) nanofibers.

### 3.3 SEM Analysis

According to the SEM results, PLA/C has a nanofiber appearance. With the increase in carvacrol, the irregularity in the nanofiber distribution increased; a decrease in the distance between the fibers was observed (Figure 3), and the nanofiber diameter increased compared to that of pure PLA.^47^ When nanofibers were examined, the thickness of pure PLA nanofibers was 2.06 μm, and with an increase in the percentage of added carvacrol, their thickness increased to approximately 3.24 μm (Figure 3a–d).

**Figure 3:**
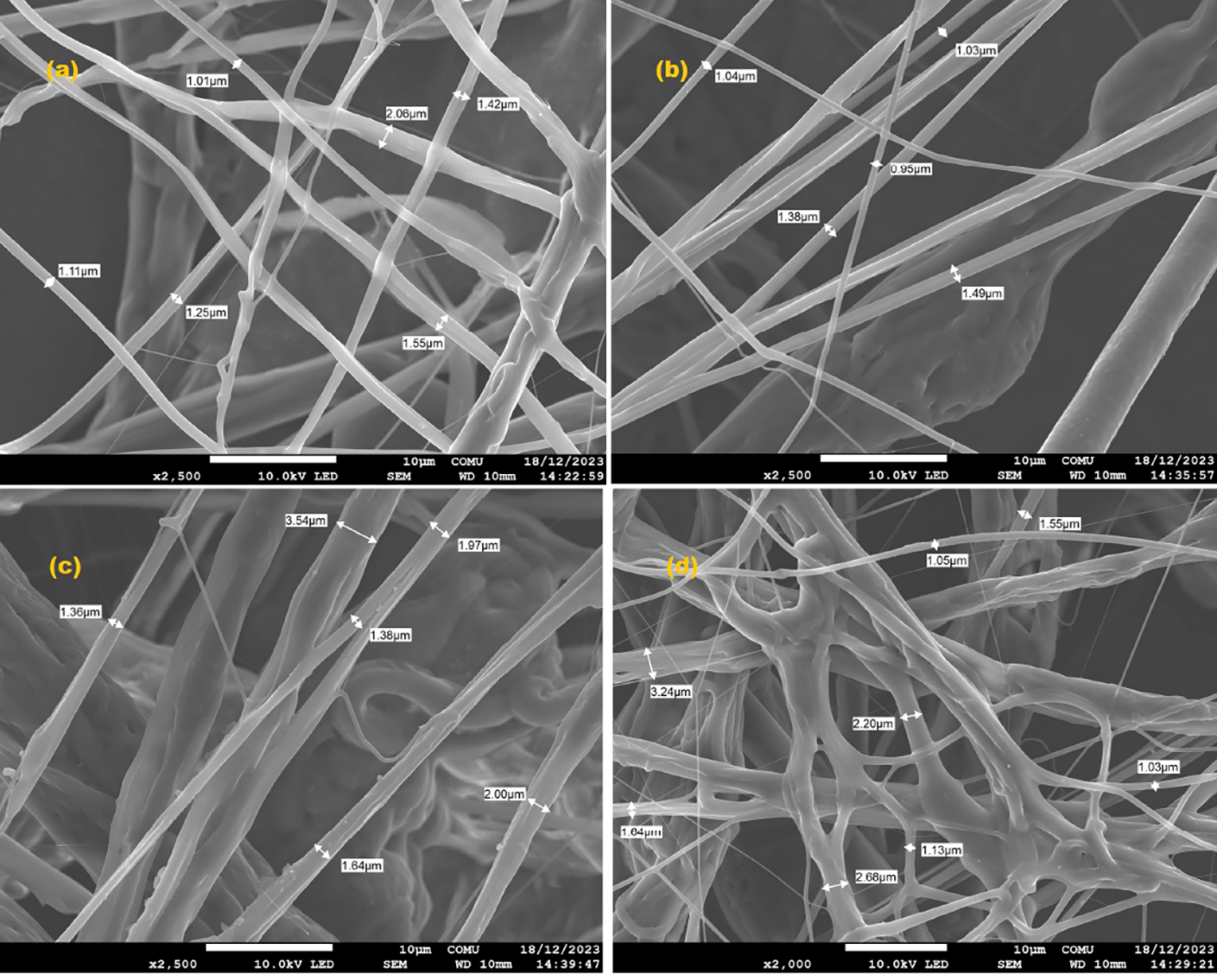
SEM images of PLA/C nanofibers with carvacrol contains 0% (a), 10% (b), 20% (c), and 30% (d).

### 3.4 In Vitro Antibacterial Test

According to the results of the “agar disk diffusion method” tests conducted to determine antibacterial effects of PLA/C nanofibers under in vitro conditions, it is shown that PLA/C nanofibers containing different amounts of carvacrol have antimicrobial effects against *E. coli* and *S. aureus* (Table 1, Figure 4).

**Figure 4:**
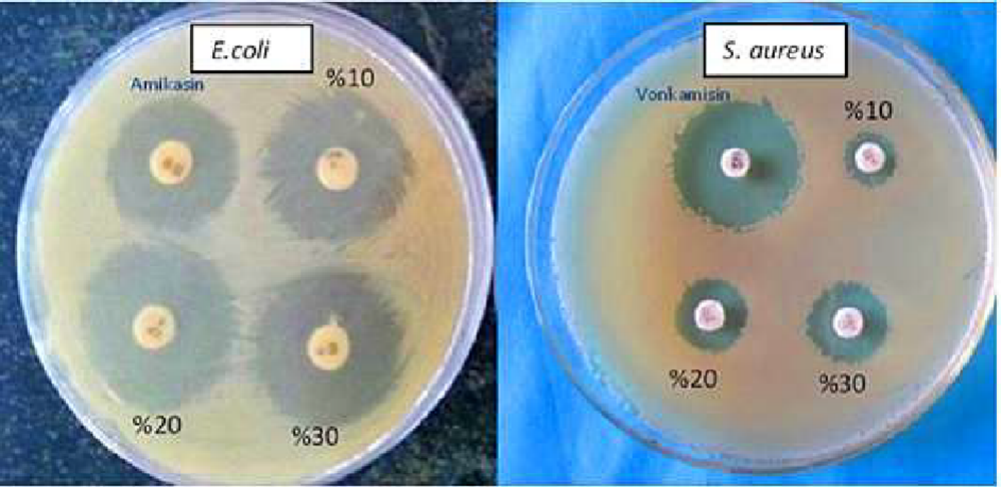
Inhibition zone images of PLA/C nanofibers containing different doses of 0% (antibiotics), 10, 20, and 30% (v/w) carvacrol against *E. coli* and *S. aureus*.

**Table 1:** Inhibition Zones of PLA/C Nanofibers Containing Different Doses of Carvacrol (0% (Antibiotics), 10, 20, 30% (v/w)) against *E. coli* and *S. aureus* (*n* = 10)^a^

	*S. aureus*	*E. coli*
PLA/C %30 (mm)	16.01 ± 0.81^b^	30.40 ± 1.09^b^
PLA/C %20 (mm)	14.23 ± 0.20^c^	32.01 ± 0.97^a^
PLA/C %10 (mm)	11.60 ± 0.21^d^	30.90 ± 1.04^b^
vancomycin (mm)	26.03 ± 0.17^a^	−
amikacin (mm)	−	26.38 ± 0.62^c^

a −: Not tested for this bacterial strain. a−d: Means within a column followed by the same letter are not significantly different at 5% level according to the Tukey test.

Samples were measured at specific time intervals (PLA/C 30%, PLA/C 20%, and PLA/C 10%) in 10 replicate measurements. The data set is presented in Table 1 as the mean and standard deviation. The analysis revealed a statistically significant difference between the measurements and the *S. aureus* bacteria. Disk analysis of PLA/C nanofibers containing different doses of carvacrol revealed a statistically significant difference (*p* < 0.05 for each). The mean lengths for PLA/C nanofibers containing 30, 20, and 10% (v/w) carvacrol were 16.01, 14.23, and 11.60 mm, respectively. Although the zones formed on PLA/C nanofibers containing carvacrol were smaller than those containing vancomycin, the zone diameters increased in parallel with the increase in the carvacrol content (Table 1). Furthermore, antibiotic (vancomycin) applications resulted in an average bacterial (*S. aureus*) growth zone of 26.03 mm. Disk diffusion tests conducted with PLA/C nanofibers containing different doses of carvacrol revealed that PLA/C nanofibers containing 30, 20, and 10% (v/w) carvacrol produced an average bacterial (*E. coli*) growth zone of 30.40, 32.01, and 30.90 mm, respectively, and treatments containing antibiotic (amikacin) produced an average bacterial (*E. coli*) growth zone of 26.38 mm.^26^ It was determined that there was a statistically significant difference between these regions, and this difference was due to the PLA/C 30% and PLA/C 10% measurements being lower than that of PLA/C 20% (Table 1).

### 3.5 TAMB Test

The TAMB test count in raw chicken meat should be at most 5.0 × 10^6^ cfu/g.^62^ The values determined were below 5.0 × 10^6^ cfu/g. According to the International Commission for Microbiological Food Standards (ICMSF), the highest acceptable limit value for TAMB count in chicken meat is 7 log 10 cfu/g,^63^ and the samples in this study did not exceed this limit value. The samples were analyzed after 7 days of cold storage (+4 °C) wrapped in PLA/C nanofibers, and TAMB values determined in PLA/C were 5 × 10^5^, 5 × 10^4^, 5 × 10^3^, and 4 × 10^3^ cfu/g for 0, 10, 20% (v/w), and 30% carvacrol, respectively (Figure 5).

**Figure 5:**
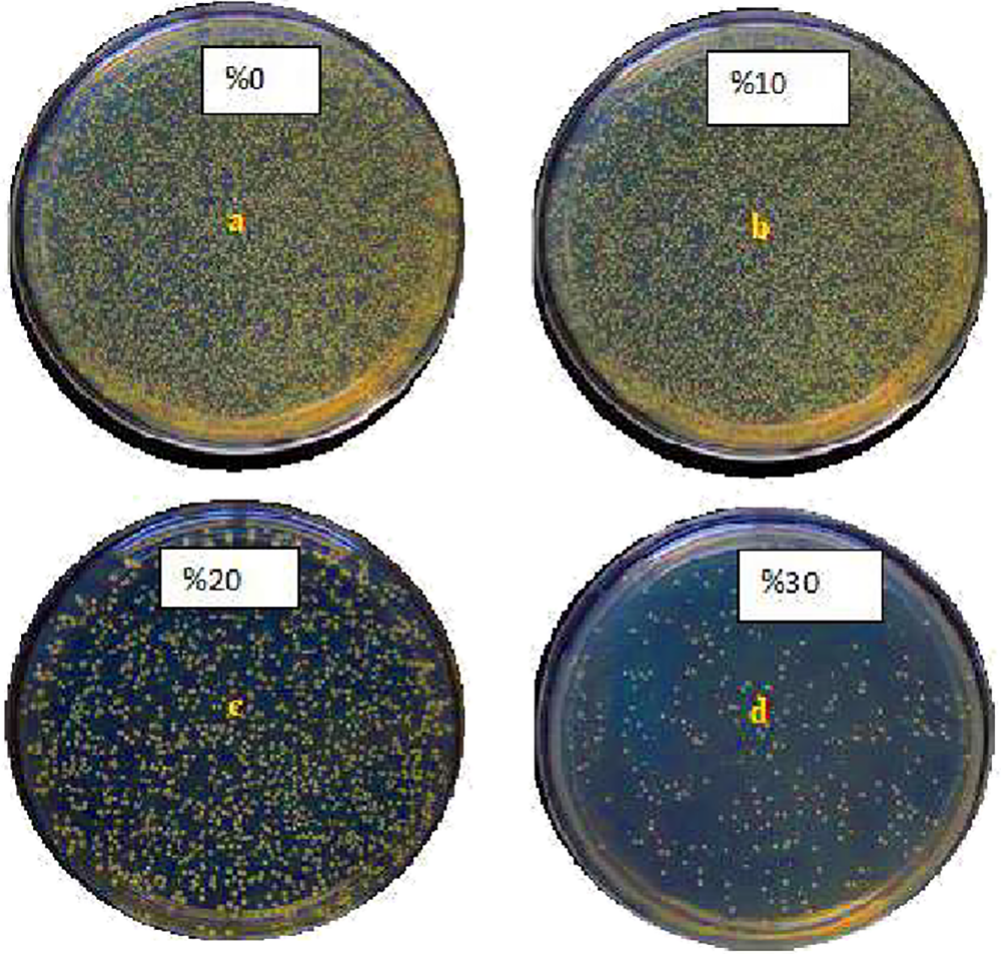
TAMB colony images in chicken breast meat samples wrapped with PLA/C nanofibers containing different amounts of 0% (a), 10% (b), 20% (c), and 30% (d) carvacrol.

The results obtained showed that at the end of seventh day, the TAMB count in the nanofiber-wrapped chicken breast meat samples was below the maximum limit specified in the regulation (Table 2). Unwrapped chicken breast meat samples (no foil, no nanofiber) were used to determine the natural spoilage rate at 4 °C. These samples showed visible deterioration and significantly higher bacterial counts approximately 24 h earlier than other groups, confirming the susceptibility of untreated meat to rapid microbial growth. PLA/C nanofibers directly applied to chicken meat without foil backing were also tested. These samples exhibited faster bacterial proliferation compared to foil-backed PLA/C samples, suggesting that the structural support and barrier properties of the foil contribute synergistically to microbial inhibition. These observations indicate that both the active antimicrobial effect of carvacrol and the physical barrier effect of the foil play a role in the shelf-life extension. However, the carvacrol-loaded PLA nanofiber remains the primary antimicrobial agent, as indicated by dose-dependent reduction in TAMB counts across all carvacrol concentrations.

**Table 2:** TAMB Count in PLA/C Nanofiber-Wrapped Chicken Breast Meat

nanofibers	TAMB count (cob/g)
PLA/C 0%	5 × 10^5^ ± 0.24
PLA/C 10%	5 × 10^4^ ± 0.62
PLA/C 20%	5 × 10^3^ ± 0.75
PLA/C 30%	4 × 10^3^ ± 0.81

### 3.6 Statistical Analysis

ANOVA (one-way) was performed to compare data groups, and Tukey’s post hoc analysis was performed for multiple comparisons, with significance at *p* < 0.05.

## 4 Conclusions

In this contribution, PLA-based nanofibers with the addition of various ratios of carvacrol (10, 20, 30% (v/w)) were successfully tested by the SBS method. The results demonstrated that the incorporation of carvacrol into the PLA polymeric solution provides active functionality. The morphological and physical properties and thermal behaviors of PLA and PLA/C (10, 20, 30% v/w) nanofibers were investigated. The presence of carvacrol in the structure of PLA/C nanofibers was confirmed by FTIR spectroscopy. In general, the nanofibers had a homogeneous morphology, and a significant increase in the nanofiber diameter (2.68–3.24 μm) was observed in line with the carvacrol content ratio. Thermal analyses demonstrated that the thermal properties of PLA and PLA/C nanofibers were feasible. Antibacterial tests of PLA nanofibers loaded with different amounts of carvacrol (10, 20, and 30% (v/w)) against *Escherichia coli* and *Staphylococcus aureus* confirmed that PLA/C nanofibers exhibit significant inhibitory effects. Furthermore, the application of PLA/C nanofibers to active packaging for chicken breast meat reduced the total bacterial load compared to control samples, demonstrating that PLA/C nanofibers play an effective role in extending the shelf life of chicken breast meat. This study demonstrates the shelf-life-enhancing effect of new PLA/C packaging with active properties on chicken breast meat, offering a new method for preserving perishable food. The widespread use of biodegradable and smart packaging systems that can be produced quickly and economically could create significant opportunities for sustainability and food safety.

## Supplementary Material



## Notes

2024 students research project evaluation list; schematic illustration of PLA/C and its effects (PDF)
