# Supplementary material for: Preparation and Characterization of Carvacrol-Loaded PLA Nanofibers by the Solution Blow-Spinning Method for the Long Shelf-Life of Chicken Breast Meat
Source: ACS Omega. 2026 Jan 23;11(5):7014–21. doi: 10.1021/acsomega.5c02604 (PMC12902995; doi:10.1021/acsomega.5c02604)
Supplement: Supplementary file 1 [file ao5c02604_si_001.pdf]

TÜBİTAK

2024 YILI LİSE ÖĞRENCİLERİ ARAŞTIRMA PROJELERİ YARIŞMASI
BÖLGE SERGİSİ PROJE DEĞERLENDİRMELERİ DERECE LİSTESİ

SIRA NO	BAŞVURU NO	ÖĞRENCİ ADI SOYADI	DANIŞMAN ADI SOYADI	OKULU	PROJENİN ADI	ANA ALANI	TEMATİK ALANI	BÖLGESİ	BÖLGE AŞAMASI SONUCU
1	1689B012405434	E** H****<br>E*** E*** A******<br>E** H**************	H*** E****	GAZİANTEP ÖZEL İSTEK GAZİANTEP FEN LİSESİ	MANYETİK POLİMER KUMAŞ TABANLI B12 VİTAMİN TESPİT CİHAZININ GELİŞTİRİLMESİ	BİYOLOJİ	Biyomedikal Cihaz Teknolojileri	ADANA	BÖLGE BİRİNCİLİĞİ
2	1689B012429113	B**** G*******<br>Ö*** C*****	İ****** E**** B****	GAZİANTEP ÖZEL SANKO KOLEJİ<br>GAZİANTEP ÖZEL SANKO FEN VE TEKNOLOJİ LİSESİ	MUSCA DOMESTİCA'NIN ONTOJENEZİNDE MİKROPLASTİK	BİYOLOJİ	Ekolojik Denge	ADANA	BÖLGE BİRİNCİLİĞİ
3	1689B012429105	Ö*** B*******<br>B**** K********<br>A**** B*****	R**** Ş**	GAZİANTEP NURAY TUNCAY KARA BİLİM VE SANAT MERKEZİ	ÇEVRE DOSTU TARIM TEKNOLOJİLERİYLE ENTEGRE BİYOLOJİK OT KONTROL SİSTEMİ	BİYOLOJİ	Tarım ve Hayvancılık Teknolojileri	ADANA	BÖLGE İKİNCİLİĞİ
4	1689B012430453	N**** D*** A******	Y****** S*********	ADANA SEYHAN BORSA İSTANBUL FEN LİSESİ	AKCİĞER KANSERİNDE HÜCRE DÖNGÜSÜNÜ DURDURARAK KEMOTERAPİ İLAÇLARININ ETKİNLERİNDEKİ DEĞİŞİKLİKLERİN İNCELENMESİ	BİYOLOJİ	Genetik ve Biyoteknoloji	ADANA	BÖLGE ÜÇÜNCÜLÜĞÜ
5	1689B012404083	M****** K**** K***<br>G**** İ*******<br>A****** P****	N****** E*******	GAZİANTEP ÖZEL SANKO KOLEJİ	YENİ NESİL BALAST SUYU YÖNETİMİ: NONİNVAZİV YÖNTEMLERLE BİYOÇEŞİTLİLİĞİ KORUMA VE BİYOLOJİK İSTİLAYI ENGELLEME SİSTEMİ	COĞRAFYA	Biyoçeşitlilik	ADANA	BÖLGE BİRİNCİLİĞİ
6	1689B012406858	E**** Ö***<br>S**** S*****<br>D**** D*** G*******	R**** K***	GAZİANTEP ÖZEL SANKO KOLEJİ	İNDİGO BOYASI GİDERİMİNDE SODA, TUZ, KAFEİN VE LİMON KABUĞU KARIŞIMI İLE HAZIRLANAN ADSORBANIN PERFORMANSI	COĞRAFYA	Çevre ve Çevreyi Koruma	ADANA	BÖLGE BİRİNCİLİĞİ
7	1689B012405881	G**** Ç****<br>P**** Ç**** G*******<br>İ*** A******	L***** K*********	MERSİN 75.YIL FEN LİSESİ<br>MERSİN TARSUS ŞEHİT HALİL ÖZDEMİR FEN LİSESİ	TOROSLARDA TETİS İZLERİ	COĞRAFYA	Doğal Miras ve Doğal Kaynaklar	ADANA	BÖLGE İKİNCİLİĞİ
8	1689B012424305	M******* T***** E*****	S**** K*****	ADANA KOZAN BİLİM VE SANAT MERKEZİ	AY IŞIĞININ DIŞ AYDINLATMA SİSTEMLERİNDE KULLANILARAK ENERJİ TASARRUFU YAPILMASI	COĞRAFYA	Sürdürülebilir Şehirler ve Toplumlar	ADANA	BÖLGE ÜÇÜNCÜLÜĞÜ
9	1689B012419645	E** M***** K***<br>E*** S*** İ****	N******* Y*****	MERSİN ERDEMLİ BİLİM VE SANAT MERKEZİ	PKETLEMEDEN KAYNAKLANAN İÇME İNEK SÜTÜ İSRAFININ BELİRLENMESİ VE ÖNLENMESİ	DEĞERLER EĞİTİMİ	Sorumlu Üretim ve Tüketim	ADANA	BÖLGE BİRİNCİLİĞİ
10	1689B012437570	E** Y****<br>S***** Ö****	G** Ş****	GAZİANTEP NURAY TUNCAY KARA BİLİM VE SANAT MERKEZİ	BARIŞ MANÇO ŞARKILARI İLE DEĞERLERİMİZİ HATIRLAYALIM	DEĞERLER EĞİTİMİ	Değerler Eğitimi	ADANA	BÖLGE İKİNCİLİĞİ
11	1689B012434412	M***** E**** K***<br>İ***** T**** A***	C**** A***** A****	GAZİANTEP NURAY TUNCAY KARA BİLİM VE SANAT MERKEZİ	MOBİL OYUN TEMELLİ CANLILARLA TÜRKÜLERİ SEVDİRELİM (GAZİANTEP YÖRESİ ÖRNEĞİ)	DEĞERLER EĞİTİMİ	Kültürel Miras	ADANA	BÖLGE ÜÇÜNCÜLÜĞÜ
12	1689B012405242	Z**** S*** Ş*******<br>M***** T**** Ş********<br>M**** U*** K*******	C**** A****	GAZİANTEP ÖZEL SANKO FEN VE TEKNOLOJİ LİSESİ	MANYETİK NANOPARTİKÜLLÜ ISI İLETEN KUMAŞ SENTEZİ	FİZİK	Giyilebilir Teknolojiler	ADANA	BÖLGE BİRİNCİLİĞİ
13	1689B012428696	A****** G****<br>M******* A** B*******	İ****** E**** B****	GAZİANTEP ÖZEL SANKO FEN VE TEKNOLOJİ LİSESİ	BETONUN DURABİLİTESİNİN BELİRLENMESİNDE TAHRİBATSIZ MUAYNENE YÖNTEMİ	FİZİK	Doğal Afetler ve Afet Yönetimi	ADANA	BÖLGE BİRİNCİLİĞİ
14	1689B012414424	H***** S****<br>M****** M******	E**** A***	MERSİN MEZİTLİ ANADOLU İMAM HATİP LİSESİ	TOPRAK NEMİ ÖLÇÜMÜ İLE TASARRUFLU TARIMSAL SULAMA	FİZİK	Tarım ve Hayvancılık Teknolojileri	ADANA	BÖLGE İKİNCİLİĞİ
15	1689B012406714	S**** Ç****<br>K**** Ç****<br>E**** E*** A*******	Ö**** G****	GAZİANTEP ÖZEL SANKO FEN VE TEKNOLOJİ LİSESİ	KARBOKSİ METİL SELÜLOZDEN ELDE EDİLEN İLETKEN HİDROJEL İLE ÇEVRE DOSTU VE BİYOUYUMLU TRİBOELEKTRİK NANOJENERATÖR TASARIMI	FİZİK	Yenilenebilir Enerji	ADANA	BÖLGE ÜÇÜNCÜLÜĞÜ
16	1689B012405416	E** G****<br>K***** G****<br>B**** B**********	C**** A****	GAZİANTEP ÖZEL SANKO FEN VE TEKNOLOJİ LİSESİ	MİKROPLASTİK VE YAĞ-SU KARIŞIMININ AYNI YÜZEYDE AYIRIMI İÇİN HDTMS MODİFİYELİ MANYETİT KAPLI ÇELİK AĞLAR	KİMYA	Malzeme Bilimi ve Nanoteknoloji	ADANA	BÖLGE BİRİNCİLİĞİ
17	1689B012412398	K**** Y****** Y****<br>C** A***	Ü****** Y****	ADANA TED ÖZEL ADANA KOLEJİ ANADOLU LİSESİ	ÇEVRE İÇİN BİR ADIM: SİGARA İZMARİTLERİNİN GERİ DÖNÜŞÜMÜYLE SUNTA ÜRETİMİ	KİMYA	Atık Yönetimi ve Geri Dönüşüm	ADANA	BÖLGE İKİNCİLİĞİ
18	1689B012406847	Y***** A****<br>İ*** A****<br>F***** N**** Y*****	İ****** K****	GAZİANTEP ÖZEL SANKO KOLEJİ<br>GAZİANTEP ÖZEL SANKO FEN VE TEKNOLOJİ LİSESİ	ENDÜSTRİYEL AĞIR METAL GİDERİMİNDE ET SUYU VE MUZ İÇERİKLİ ADSORBAN MALZEME SENTEZİ VE UYGULANMASI	KİMYA	Malzeme Bilimi ve Nanoteknoloji	ADANA	BÖLGE ÜÇÜNCÜLÜĞÜ
19	1689B012426742	İ***** A*******		MERSİN ÖZEL TARSUS AMERİKAN KOLEJİ	RAMANUJAN'IN ANA TEOREMİ, KESİRLİ TÜREVLER VE GENELLEŞTİRİLMİŞ BİR İNTEGRALİN HESAPLANMASI	MATEMATİK	STEAM (Fen, Teknoloji, Mühendislik, Sanat ve Matematik)	ADANA	BÖLGE BİRİNCİLİĞİ
20	1689B012419574	E** Ö******<br>İ*** K****	H***** K********	GAZİANTEP NURAY TUNCAY KARA BİLİM VE SANAT MERKEZİ	İTERATİF SAYI DİYAGRAMLARI YARDIMIYLA XOR İŞLEMLİ ŞİFRELEME ALGORİTMASI	MATEMATİK	Özgün Algoritma Tasarımı	ADANA	BÖLGE İKİNCİLİĞİ
21	1689B012417908	M***** T****	M****** B********	MERSİN EYÜP AYGAR FEN LİSESİ	PÜSK KRİPTOLOJİ ALGORİTMASI	MATEMATİK	Özgün Algoritma Tasarımı	ADANA	BÖLGE ÜÇÜNCÜLÜĞÜ
22	1689B012431365	S**** T*****<br>İ**** D*********<br>P**** B******	S**** A***	GAZİANTEP NURAY TUNCAY KARA BİLİM VE SANAT MERKEZİ	HELİKOPTER EBEVEYNLİK VE OKUL TÜKENMİŞLİĞİ İLİŞKİSİNDE ÖZ-KONTROLÜN ARACILIK ROLÜNÜN İNCELENMESİ	PSİKOLOJİ	Değerler Eğitimi	ADANA	BÖLGE BİRİNCİLİĞİ
23	1689B012421998	H******** B********<br>Ü**** K**** B******	M******* S**** T*******	GAZİANTEP ŞEHİTKAMİL BELEDİYESİ KIZ ANADOLU İMAM HATİP LİSESİ	KELİME SPORU: ZİHİNSEL FİTNESS	PSİKOLOJİ	Yabancı Dil Eğitimi	ADANA	BÖLGE İKİNCİLİĞİ
24	1689B012434107	F*** G******<br>B****** V*** K*********	A*** Z*****	ADANA CEMİL MERİÇ ANADOLU LİSESİ	POZİTİF PSİKOLOJİ ETKİNLİKLERİ YOLUYLA YABANCI DİL BECERİLERİNİN GELİŞTİRİLMESİ: İNGİLİZCE DERSLERİNE YENİ BİR YAKLAŞIM	PSİKOLOJİ	Yabancı Dil Eğitimi	ADANA	BÖLGE ÜÇÜNCÜLÜĞÜ
25	1689B012418932	S*** A****<br>B**** E*** Y****	N***** Ö*****	GAZİANTEP ŞAHİNBEY BELEDİYESİ BİLİM VE SANAT MERKEZİ<br>GAZİANTEP ÖZEL GAZİANTEP KAVRAM FEN LİSESİ	DİJİTAL KÜLTÜRÜN FARKLI SOSYO EKONOMİK DÜZEYDEKİ ÖĞRENCİLERİN YARATIM SÜRECİNE ETKİLERİ	SOSYOLOJİ	Görsel ve İşitsel Sanatlar	ADANA	BÖLGE BİRİNCİLİĞİ
26	1689B012419240	Y**** Ç*****<br>E**** D****	İ***** C*****	K.K.T.C. HALA SULTAN İLAHİYAT KOLEJİ	6 ŞUBAT 2023 DEPREMLERİ SONRASI KKTC'YE GÖÇ EDEN ÖĞRENCİLERİN UYUM VE ÖZNEL İYİ OLUŞLARININ İNCELENMESİ	SOSYOLOJİ	Göç ve Uyum	ADANA	BÖLGE BİRİNCİLİĞİ
27	1689B012421632	M***** S*****<br>Z***** A** Y********	H***** K********	GAZİANTEP NURAY TUNCAY KARA BİLİM VE SANAT MERKEZİ	GÖRME ENGELLİ BİREYLERİN MERKEZİ SINAVLARDA KARŞILAŞTIĞI SORUNLARA YENİLİKÇİ ÇÖZÜMLER: MOBİL SINAV UYGULAMASI	SOSYOLOJİ	Dijital Dönüşüm	ADANA	BÖLGE İKİNCİLİĞİ
28	1689B012429044	Ü*** Ş**** Ö****<br>Ş***** C*****<br>E*** İ****	A****** Y******* B****	ADANA ÇUKUROVA BİLİM VE SANAT MERKEZİ	ALMANYA'DA YAŞAYAN TÜRK AİLELERİ VE 3. KUŞAK GENÇLERİN ÇEŞİTLİ OLGULAR AÇISINDAN İNCELENMESİ: REUTLİNGEN ÖRNEĞİ.	SOSYOLOJİ	Göç ve Uyum	ADANA	BÖLGE ÜÇÜNCÜLÜĞÜ

SIRA NO	BAŞVURU NO	ÖĞRENCİ ADI SOYADI	DANIŞMAN ADI SOYADI	OKULU	PROJENİN ADI	ANA ALANI	TEMATİK ALANI	BÖLGESİ	BÖLGE AŞAMASI SONUCU
29	1689B012421026	E*** S*** D***	M****** K**** K*****	ADANA ADANA ANADOLU LİSESİ	TARİHİ İNŞA ETMEK 6 ŞUBAT DEPREMİNDE YIKILAN TARİHİ ESERLER EKSENİNDE TARİH ÖĞRETİMİ	TARİH	Kültürel Miras	ADANA	BÖLGE BİRİNCİLİĞİ
30	1689B012431309	A********* T******<br>D**** Y*****	M****** G******	GAZİANTEP NİZİP MESLEKİ VE TEKNİK ANADOLU LİSESİ	NİZİP'TEKİ TARİHİ SABUNHANELERİN SOSYO-EKONOMİK VE KÜLTÜREL HAYATA YENİDEN KAZANDIRILMASI	TARİH	Kültürel Miras	ADANA	BÖLGE İKİNCİLİĞİ
31	1689B012418923	A** F**** A*****<br>U*** B*******	B***** Z*****	GAZİANTEP ŞAHİNBEY BELEDİYESİ BİLİM VE SANAT MERKEZİ	SEMBOLLERİN DİLİNDE YÜZLERCE YILLIK MİRASIN İZİNDE: GELENEKSEL GAZİANTEP EV KAPILARI VE TOKMAKLARI	TARİH	Kültürel Miras	ADANA	BÖLGE ÜÇÜNCÜLÜĞÜ
32	1689B012400101	F***** M***** Y*****<br>E****** E******<br>H**** E****	P****** Ö*****	ADANA SEYHAN ROTARY ANADOLU LİSESİ	ENGEL KARŞITI LOJİSTİK ARAÇ (EKAR)	TEKNOLOJİK TASARIM	Robotik ve Kodlama	ADANA	BÖLGE BİRİNCİLİĞİ
33	1689B012414670	A**** K********<br>T*** M****** K****<br>N** Y*****	Ö**** E*****	GAZİANTEP GAZİKENT ANADOLU LİSESİ	KATLANIR SINIF	TEKNOLOJİK TASARIM	STEAM (Fen, Teknoloji, Mühendislik, Sanat ve Matematik)	ADANA	BÖLGE BİRİNCİLİĞİ
34	1689B012410947	A** G***** G**<br>M*** M*****	Y**** B*****	OSMANİYE KADİRLİ BİLİM VE SANAT MERKEZİ	ÇİÇEK BAKIMI UZMANI	TEKNOLOJİK TASARIM	STEAM (Fen, Teknoloji, Mühendislik, Sanat ve Matematik)	ADANA	BÖLGE İKİNCİLİĞİ
35	1689B012420799	A*** E*** N*****	Z**** Ş******	MERSİN İÇEL ANADOLU LİSESİ	MİKROPLASTİK İÇERMEYEN , KOMPOSTLAŞTIRILABİLEN, ÇEVRE DOSTU, MUZ LİFİ İÇERİKLİ POŞET ÇAY AMBALAJI	TEKNOLOJİK TASARIM	Malzeme Bilimi ve Nanoteknoloji	ADANA	BÖLGE ÜÇÜNCÜLÜĞÜ
36	1689B012401310	D****** M*** A****<br>T*** Z**** K*******<br>M***** Ü***	H**** M****	ADANA ÇUKUROVA BİLİM VE SANAT MERKEZİ	ANLATI BİLİMİ VE YAPAY ZEKA	TÜRK DİLİ VE EDEBİYATI	Yapay Zekâ	ADANA	BÖLGE BİRİNCİLİĞİ
37	1689B012433436	N**** G***	Z***** A***** K******	KİLİS KİLİS BİLİM VE SANAT MERKEZİ	EXLİBRİS İLE KARAKTER TAHLİLİ	TÜRK DİLİ VE EDEBİYATI	Görsel ve İşitsel Sanatlar	ADANA	BÖLGE BİRİNCİLİĞİ
38	1689B012418284	Y******* Ç*****	B***** Ç***** A****	MERSİN TOROSLAR BİLİM VE SANAT MERKEZİ	GERÇEKTENKURGUYA, KURGUDAN SANALA: YÖRÜK KÜLTÜRÜNDEN ÖRNEKLER	TÜRK DİLİ VE EDEBİYATI	Kültürel Miras	ADANA	BÖLGE İKİNCİLİĞİ
39	1689B012436035	E*** A*****	M***** Ö***	GAZİANTEP NURAY TUNCAY KARA BİLİM VE SANAT MERKEZİ	METAVERSE YENİLİĞİ: DİL EĞİTİMİNDE DÖNÜŞÜM	TÜRK DİLİ VE EDEBİYATI	Yabancı Dil Eğitimi	ADANA	BÖLGE ÜÇÜNCÜLÜĞÜ
40	1689B012417589	T******* E*****<br>M****** D**	Ö**** Y***	ADANA HACI HATİCE TURGUT ANADOLU LİSESİ<br>MERSİN ÖZEL MERSİN BAHÇEŞEHİR ANADOLU LİSESİ	GİZEMLİ UZAY MACERASI: ÖZEL GEREKSİNİMLİ ÇOCUKLAR İÇİN SANAL GERÇEKLİKTE REHABİLİTASYON OYUNU	YAZILIM	Artırılmış, Sanal ve Karma Gerçeklik	ADANA	BÖLGE BİRİNCİLİĞİ
41	1689B012420561	Y**** C** T****	M***** B****	MERSİN EYÜP AYGAR FEN LİSESİ	MİNDMOVER3D: ÇOKLU UZAMSAL BECERİ PLATFORMU	YAZILIM	Özgün Algoritma Tasarımı	ADANA	BÖLGE İKİNCİLİĞİ
42	1689B012400239	D**** U*** Ü***<br>N***** A** E******<br>B**** T***	O**** Ç*****	ADANA ÇUKUROVA BİLİM VE SANAT MERKEZİ	YAYDES: İLACIN BENDE	YAZILIM	Nesnelerin İnterneti	ADANA	BÖLGE ÜÇÜNCÜLÜĞÜ
43	1689B012417902	E***** K******<br>A**** B*** Ç*****<br>F**** E** A******	P**** K****** S***	BOLU BOLU FEN LİSESİ	ÇİNKO OKSİT (ZNO) VE TİTANYUM DİOKSİT (TİO2) NANOPARTİKÜL UYGULAMASININ IZA BUĞDAYININ (TRİTİCUM MONOCOCCUM SSP. MONOCOCCUM) ÇİMLENME VE FİTOKİMYASAL İÇERİĞİNE ETKİLERİ	BİYOLOJİ	Tarım ve Hayvancılık Teknolojileri	ANKARA	BÖLGE BİRİNCİLİĞİ
44	1689B012434690	C** H****	G**** E******** E****	ANKARA TED ANKARA KOLEJİ VAKFI ÖZEL LİSESİ	ZOPHOBAS MORİO KURTÇUKLARI İLE POLİSTİREN ATIK TESİSİ	BİYOLOJİ	Atık Yönetimi ve Geri Dönüşüm	ANKARA	BÖLGE BİRİNCİLİĞİ
45	1689B012402518	E** C****	B**** K*****	ANKARA ANKARA ÜNİVERSİTESİ GELİŞTİRME VAKFI OKULLARI ÖZEL ANADOLU LİSESİ	YEŞİL BİYOSENTEZLE TERMOFİLİK AERIBACILLUS PALLIDUS'DAN ÜRETİLEN GÜMÜŞ NANOPARTİKÜLLERİN ANTİMİKROBİYAL ÖZELLİKLİ YÜZEYLERDE KULLANIM POTANSİYELİ	BİYOLOJİ	Genetik ve Biyoteknoloji	ANKARA	BÖLGE İKİNCİLİĞİ
46	1689B012424216	B**** A*****	Ü*** A**	ANKARA ÖZEL ANKARA SANAYİ ODASI (ASO) TEKNİK KOLEJİ MESLEKİ VE TEKNİK ANADOLU LİSESİ	BİLYALI KEKİK UÇUCU YAĞININ AKDENİZ MEYVE SİNEĞİ VE KAHVERENGİ KOKARCA ÜZERİNE FUMİGANT ETKİSİNİN ARAŞIRILMASI	BİYOLOJİ	Ekolojik Denge	ANKARA	BÖLGE ÜÇÜNCÜLÜĞÜ
47	1689B012409569	N**** A***<br>Z***** Ö*** E****<br>D*** I**** Ö****	Y****** K***** Ç******	ANKARA ÖZEL BEŞTEPE ANADOLU LİSESİ	AKILLI JEL TOPRAK BULUTU	COĞRAFYA	Tarım ve Hayvancılık Teknolojileri	ANKARA	BÖLGE BİRİNCİLİĞİ
48	1689B012417008	B**** S****<br>D**** O***<br>M***** E***	Y**** G****	KIRIKKALE KIRIKKALE İL ÖZEL İDARESİ BİLİM VE SANAT MERKEZİ	ÇEVRE SORUNLARINA FARKINDALIK OLUŞTURMADA DİJİTAL ÖYKÜLEME ARAÇLARININ ETKİNLİĞİNİN ARAŞTIRILMASI	COĞRAFYA	Çevre ve Çevreyi Koruma	ANKARA	BÖLGE İKİNCİLİĞİ
49	1689B012402172	D**** S* D*******<br>B***** I**** Ö***	D**** D****	ANKARA ETİMESGUT BİLİM VE SANAT MERKEZİ	KAZ DAĞI MODELLEMESİNDEN BİYOÇEŞİTLİLİĞE	COĞRAFYA	Biyoçeşitlilik	ANKARA	BÖLGE ÜÇÜNCÜLÜĞÜ
50	1689B012438826	Z**** Y*****<br>J**** A***** N****<br>E**** E*** Ç*****	H***** E*** U***	ANKARA ULUS İLK MECLİS ANADOLU İMAM HATİP LİSESİ	DÜŞÜNCE EĞİTİMİNE YÖNELİK KARAKALEM ÇİZGİ ROMAN TASARIMI: MİNERVA İLE SİSİFOS'UN YOLCULUĞU	DEĞERLER EĞİTİMİ	Değerler Eğitimi	ANKARA	BÖLGE BİRİNCİLİĞİ
51	1689B012424177	F***** N** S*******<br>M***** B*** S*****	L****** E*******	ANKARA ŞEHİT ÖMER HALİSDEMİR ANADOLU İMAM HATİP LİSESİ	MARİFET İLTİFATA TABİDİR	DEĞERLER EĞİTİMİ	Değerler Eğitimi	ANKARA	BÖLGE İKİNCİLİĞİ
52	1689B012422538	E*** C**** E****	N**** P*******	ANKARA SABAHATTİN ZAİM SOSYAL BİLİMLER LİSESİ	OKULUMDA MEDDAH VAR!	DEĞERLER EĞİTİMİ	Kültürel Miras	ANKARA	BÖLGE ÜÇÜNCÜLÜĞÜ
53	1689B012416998	O*** A***	A**** A****	ZONGULDAK KDZ. EREĞLİ ŞAHİNDE HAYRETTİN YAVUZ BİLİM VE SANAT MERKEZİ	SERALARDA YENİLİKÇİ ISI KONTROLÜ: YENİ BOYA TEKNOLOJİSİYLE ENERJİ VERİMLİLİĞİ	FİZİK	Tarım ve Hayvancılık Teknolojileri	ANKARA	BÖLGE BİRİNCİLİĞİ
54	1689B012433353	A** K**** K******	E**** D*****	ANKARA ANKARA FEN LİSESİ	BİYOMİMİKRİ İLE SÜRTÜNME KATSAYISI DÜŞÜK UÇAK TASARIMI	FİZİK	STEAM (Fen, Teknoloji, Mühendislik, Sanat ve Matematik)	ANKARA	BÖLGE BİRİNCİLİĞİ
55	1689B012416790	K******* K******<br>T*** S**** A******		ANKARA KEÇİÖREN VATANSEVER ŞEHİT TÜMGENERAL AYDOĞAN AYDIN FEN LİSESİ	ISPANAK VE HAVUÇ ATIKLARINDAN SIVI TABANLI ORGANİK GÜNEŞ PANELİ	FİZİK	Yenilenebilir Enerji	ANKARA	BÖLGE İKİNCİLİĞİ
56	1689B012420627	C*** G*****	V**** G**	ANKARA TED ANKARA KOLEJİ VAKFI ÖZEL LİSESİ	BİYOTAKLİT YAKLAŞIMLA KANAT PROFİLİNİN ETKİSİNİN İNCELENMESİ VE UÇAK TASARIMI	FİZİK	Havacılık ve Uzay Bilimleri	ANKARA	BÖLGE ÜÇÜNCÜLÜĞÜ
57	1689B012416233	M***** A*** B******<br>D**** Ç*****	Ü********* A******	ANKARA ÖZEL ERYAMAN OKYANUS ANADOLU LİSESİ	SAĞLIKTA ENGİNAR MUCİZESİ	KİMYA	Gıda ve Gıda Arzı Güvenliği	ANKARA	BÖLGE BİRİNCİLİĞİ
58	1689B012432604	B*** D****<br>D**** B**** D******<br>E*** E***	B**** A***** Ö******	ZONGULDAK PROF. DR. ŞABAN TEOMAN DURALI BİLİM VE SANAT MERKEZİ	ZARGANA BALIĞI (BELONE BELONE) KILÇIĞINDAN ELDE EDİLEN KUANTUM NOKTALARIN FLORESANS ÖZELLİKLERİNİN ATIK SULARDA HG(II) İYONLARININ BELİRLENMESİNDE KULLANILMASI	KİMYA	Malzeme Bilimi ve Nanoteknoloji	ANKARA	BÖLGE BİRİNCİLİĞİ
59	1689B012418771	A**** T**** A******	M***** K***** D***	ANKARA TED ANKARA KOLEJİ VAKFI ÖZEL LİSESİ	KEVLAR POLİMERİNE ALTERNATİF ÜRÜN GELİŞTİRİLMESİ VE GELİŞTİRİLEN ÜRÜNÜN ÖZELLİKLERİNİN TEST EDİLMESİ	KİMYA	Malzeme Bilimi ve Nanoteknoloji	ANKARA	BÖLGE İKİNCİLİĞİ

SIRA NO	BAŞVURU NO	ÖĞRENCİ ADI SOYADI	DANIŞMAN ADI SOYADI	OKULU	PROJENİN ADI	ANA ALANI	TEMATİK ALANI	BÖLGESİ	BÖLGE AŞAMASI SONUCU
60	1689B012431960	A**** B*****<br>H**** K**** A*	A*** E******	ANKARA TED ANKARA KOLEJİ VAKFI ÖZEL LİSESİ	MG-AL TABAKALI ÇİFT HİDROKSİTLERİ İLE 2,4-DİKLOROFENOKSİ ASETİK ASİDİN SULARDAN UZAKLAŞTIRILMASI VE DESORBSİYONU İLE GERİ KAZANIMI	KİMYA	Ekolojik Denge	ANKARA	BÖLGE ÜÇÜNCÜLÜĞÜ
61	1689B012418398	Y**** E*** E****<br>K****** A******	Ü********* A*******	ANKARA SİNCAN ŞEHİT ABDULLAH BÜYÜKSOY BİLİM VE SANAT MERKEZİ	POZİTİF İNDİSLİ FİBONACCİ SAYILARI İLE POZİTİF TAM SAYILARIN ZECKENDORF GÖSTERİMLERİYLE ÖZGÜN BİR ŞİFRELEME ALGORİTMASI	MATEMATİK	Özgün Algoritma Tasarımı	ANKARA	BÖLGE BİRİNCİLİĞİ
62	1689B012436282	T***** Ö**** G********<br>S*** S**** İ***	S***** S********	ANKARA ETİMESGUT BİLİM VE SANAT MERKEZİ	DEĞİŞKEN MATRİSLİ ŞİFRELEME	MATEMATİK	Özgün Algoritma Tasarımı	ANKARA	BÖLGE BİRİNCİLİĞİ
63	1689B012424454	Z**** E*** A******	M****** K**** İ***	ZONGULDAK KDZ. EREĞLİ ŞAHİNDE HAYRETTİN YAVUZ BİLİM VE SANAT MERKEZİ	EKOKU BELLİ OLAN N TAMSAYI ÜZERİNE	MATEMATİK	Özgün Algoritma Tasarımı	ANKARA	BÖLGE İKİNCİLİĞİ
64	1689B012400058	E**** T*** B*****<br>D**** B******<br>M****** Y*****	R**** A****	ANKARA ÇUBUK BİLİM VE SANAT MERKEZİ<br>ANKARA ANKARA ATATÜRK LİSESİ<br>ANKARA ÇUBUK YILDIRIM BEYAZIT ANADOLU LİSESİ	FİBONACCİ, LUCAS, PELL VE PELL-LUCAS SAYILARININ, DOĞRU, DÜZLEM VE POLİNOMLARDA UYGULANMASI	MATEMATİK	Özgün Algoritma Tasarımı	ANKARA	BÖLGE ÜÇÜNCÜLÜĞÜ
65	1689B012424172	M**** F***** U***	H***** A******* D****	ANKARA SİNCAN ŞEHİT ABDULLAH BÜYÜKSOY BİLİM VE SANAT MERKEZİ	ÖĞRENCİLERİNİN ÇATIŞMA ÇÖZME BECERİ DÜZEYLERİNİ ARTIRMAYA YÖNELİK BİR ÇALIŞMA;MOBİUS'DA YOLCULUK	PSİKOLOJİ	İnsan Hakları ve Demokrasi	ANKARA	BÖLGE BİRİNCİLİĞİ
66	1689B012429560	E*** Y****<br>A*** N**** Ç****	E*** A*****	ANKARA YENİMAHALLE BİLİM VE SANAT MERKEZİ	BARBİE BEBEK ÜZERİNDEN İNŞA EDİLEN POPÜLER KÜLTÜRÜN ANNE ÇOCUK BAĞLAMINDA ANALİZİ	PSİKOLOJİ	Medya Okuryazarlığı	ANKARA	BÖLGE İKİNCİLİĞİ
67	1689B012432838	K*** D**** E****<br>E** E*** Y******	G**** E******** E****	ANKARA TED ANKARA KOLEJİ VAKFI ÖZEL LİSESİ	LİSE ÖĞRENCİLERİNDE İNTİHALE İLİŞKİN TUTUM VE ÜRETKEN YAPAY ZEKA PLATFORMU KULLANIMI	PSİKOLOJİ	Yapay Zekâ	ANKARA	BÖLGE ÜÇÜNCÜLÜĞÜ
68	1689B012401398	M***** S*** Ü***<br>Z***** E** A***	M******* D*******	ANKARA YENİMAHALLE BİLİM VE SANAT MERKEZİ	KADINA YÖNELİK ŞİDDETİN ENGELLENMESİNDE OKULLARDA PEDAGOJİK BİR ARAÇ OLARAK İMGE TİYATROSUNUN KULLANIMI	SOSYOLOJİ	İnsan Hakları ve Demokrasi	ANKARA	BÖLGE BİRİNCİLİĞİ
69	1689B012437376	D**** A****	A**** D****	ANKARA TED ANKARA KOLEJİ VAKFI ÖZEL LİSESİ	CHAT GPT'NİN EĞİTİM ORTAMLARINDA KULLANILMASINA İLİŞKİN LİSE ÖĞRENCİLERİNİN GÖRÜŞLERİNİN BELİRLENMESİ (TED ANKARA KOLEJİ ÖRNEĞİ)	SOSYOLOJİ	Dijital Dönüşüm	ANKARA	BÖLGE İKİNCİLİĞİ
70	1689B012406907	E****** A****	M**** K***** Ç*****	BOLU BOLU BİLİM VE SANAT MERKEZİ	BOLU AŞININ TOPLUMSAL YOLCULUĞU	SOSYOLOJİ	Kültürel Miras	ANKARA	BÖLGE ÜÇÜNCÜLÜĞÜ
71	1689B012419913	A** T*********	N***** G*******	ANKARA ÖZEL ANKARA MAYA FEN VE TEKNOLOJİ LİSESİ	YAPAY ZEKA DESTEKLİ DİJİTAL ÇİZGİ ROMANLA TÜRK'ÜN MAKUS TALİHİNİ YENDİĞİ GÜNLERE YOLCULUK	TARİH	Yapay Zekâ	ANKARA	BÖLGE BİRİNCİLİĞİ
72	1689B012407208	E*** Ç****	E**** E*** Ö***	ANKARA ODTÜ GELİŞTİRME VAKFI ÖZEL LİSESİ	NAKKAŞIN GÖNLÜNDEN KAĞIDA NAKŞOLAN SESSİZ DİL: KÜLTÜR MİRASIMIZ MİNYATÜR SANATI SANAL MÜZE ÖRNEĞİ	TARİH	Kültürel Miras	ANKARA	BÖLGE İKİNCİLİĞİ
73	1689B012418021	A**** E** I******<br>L**** A** Y********<br>N**** G****	S**** U*** G**	ANKARA HACI ÖMER TARMAN ANADOLU LİSESİ	EVLİYA ÇELEBİ'NİN ANKARA'SINDAN GÜNÜMÜZ ANKARA'SINA KALAN DOĞAL VE SOMUT KÜLTÜREL MİRAS	TARİH	Kültürel Miras	ANKARA	BÖLGE ÜÇÜNCÜLÜĞÜ
74	1689B012409337	C**** Y**** A*******<br>M***** E*** K****<br>O**** T*** A****	İ**** D****	ANKARA ÖZEL ANKARA SANAYİ ODASI (ASO) TEKNİK KOLEJİ MESLEKİ VE TEKNİK ANADOLU LİSESİ	MANİPÜLATÖRLÜ BOMBA İMHA İHA'SI	TEKNOLOJİK TASARIM	Havacılık ve Uzay Bilimleri	ANKARA	BÖLGE BİRİNCİLİĞİ
75	1689B012434771	Ö*** E*******<br>U*** S*******<br>A*** Y*****	T****** D******	ZONGULDAK ZONGULDAK BORSA İSTANBUL ANADOLU LİSESİ<br>ZONGULDAK ZONGULDAK FEN LİSESİ<br>ZONGULDAK KOZLU ANADOLU LİSESİ	GÜDÜLENMİŞ YAPAY ZEKâ İLE ELEKTRONİK HARP YÖNTEMLERİNDEN ETKİLENMEYEN TAM OTONOM İNSANSIZ HAVA ARACI YAPIMI	TEKNOLOJİK TASARIM	Havacılık ve Uzay Bilimleri	ANKARA	BÖLGE BİRİNCİLİĞİ
76	1689B012424079	D**** B*********<br>Y**** S****** Y*****	A**** E****	ANKARA ÖZEL ÇANKAYA BAHÇEŞEHİR KOLEJİ 50.YIL FEN VE TEKNOLOJİ LİSESİ	SU SAYACI İLE SUDA RİSK TESPİTİ	TEKNOLOJİK TASARIM	Su Okuryazarlığı	ANKARA	BÖLGE İKİNCİLİĞİ
77	1689B012432771	İ**** K***<br>B***** T**	Y******* T********	BARTIN MEHMET AKİF ERSOY MESLEKİ VE TEKNİK ANADOLU LİSESİ	BİR DIŞ CEPHE MİMARİSİ: DOĞADAN İLHAMLA AKILLI MAŞRABİYE TEKNİĞİ	TEKNOLOJİK TASARIM	Biyotaklit	ANKARA	BÖLGE ÜÇÜNCÜLÜĞÜ
78	1689B012402729	A**** D**** S****<br>M*** Z*****	C**** K******	KIRIKKALE PROF.TBP.TÜM GENERAL DERVİŞ ŞEN ÇOK PROGRAMLI ANADOLU LİSESİ	ÇOCUKLARIN GÖZÜNDEN ATASÖZLERİ	TÜRK DİLİ VE EDEBİYATI	Oyun ve Oyunlaştırma	ANKARA	BÖLGE BİRİNCİLİĞİ
79	1689B012431245	E*** A***<br>F***** U*** K*****	F****** E****	KARABÜK DEMİR ÇELİK ANADOLU LİSESİ	EFSANE ÖRNEKLERİYLE ENGELSİZ EDEBİYAT	TÜRK DİLİ VE EDEBİYATI	Dijital Dönüşüm	ANKARA	BÖLGE BİRİNCİLİĞİ
80	1689B012405458	Y***** Y*****<br>A**** B*** K******	T**** A****	BOLU BOLU BİLİM VE SANAT MERKEZİ	DEYİM, NASIL ÖĞRETEYİM? (DEYİM ÖĞRETİM YÖNTEMLERİ)	TÜRK DİLİ VE EDEBİYATI	Dil ve Edebiyat	ANKARA	BÖLGE İKİNCİLİĞİ
81	1689B012424802	Z***** Y**** K*****<br>Z**** N** Z*****	E*** A****** A****	ANKARA PURSAKLAR ANADOLU İMAM HATİP LİSESİ	KÜLTÜREL ZENGİNLİKTEKİ HAZİNE- ESKİMEYEN DEĞERLERİN İZİNDE: GELENEKLİ ÇOCUK OYUNLARI	TÜRK DİLİ VE EDEBİYATI	Kültürel Miras	ANKARA	BÖLGE ÜÇÜNCÜLÜĞÜ
82	1689B012423908	E** Y*******	Z*** C*****	ANKARA PROF. DR. AZİZ SANCAR BİLİM VE SANAT MERKEZİ	TÜRKÇEMETRE: KİŞİSELLEŞTİRİLMİŞ E-TEST SİSTEMİ	YAZILIM	Dil ve Edebiyat	ANKARA	BÖLGE BİRİNCİLİĞİ
83	1689B012437372	F***** S**** T*******<br>Y**** T***	K**** Ç****	ANKARA ANKARA FEN LİSESİ	POLİRİTİM BENZETİMİ KULLANARAK ELEKTROKARDİYOGRAFİYİ YORUMLAYAN YAPAY ZEKâ MODELİ	YAZILIM	Özgün Algoritma Tasarımı	ANKARA	BÖLGE İKİNCİLİĞİ
84	1689B012434308	B**** A***<br>M****** A*** B**	S**** C***** D********	ANKARA NERMİN MEHMET ÇEKİÇ ANADOLU LİSESİ<br>ANKARA ÖZEL YİRMİ DOKUZ MAYIS FERDA FEN LİSESİ	VİSİONEDDRAW: SANAT EĞİTİMİ ALANINDA YENİLİKÇİ ÖLÇME VE DEĞERLENDİRME YAZILIMI	YAZILIM	Görsel ve İşitsel Sanatlar	ANKARA	BÖLGE ÜÇÜNCÜLÜĞÜ
85	1689B012434601	G**** E**********<br>İ*** N** P*******<br>N*** N** Ö****	T**** T*****	ESKİŞEHİR ESKİŞEHİR ANADOLU LİSESİ	NAZOKOMİYAL ENFEKSİYONLARDA KULLANILABİLECEK DEFNE VE ÖKSE OTU EKSTRESİ YÜKLÜ İLAÇ TAŞIYICI SİSTEMLERİN GELİŞTİRİLMESİ	BİYOLOJİ	Biyomedikal Cihaz Teknolojileri	BURSA	BÖLGE BİRİNCİLİĞİ
86	1689B012435653	B**** Ç****<br>Z***** D*****<br>G**** S*** A******	S**** D*****	BALIKESİR SIRRI YIRCALI ANADOLU LİSESİ<br>BALIKESİR ALTIEYLÜL FEN LİSESİ	VAJİNAL KANDİDİYAZİS TESPİT PEDİ İLE KONTROLLÜ SALINIMLI MİKROKAPSÜLLENMİŞ ÜZÜM ÇEKİRDEĞİ YAĞI İÇEREN VAJİNAL TAMPON VE KREM	BİYOLOJİ	Halk Sağlığı ve Koruyucu Sağlık Hizmetleri	BURSA	BÖLGE BİRİNCİLİĞİ
87	1689B012436763	M**** C**** B****<br>A***** Ö******	P**** S****	BALIKESİR ŞEHİT PROF. DR. İLHAN VARANK BİLİM VE SANAT MERKEZİ	GRAFEN KUANTUM NOKTA TEMELLİ VOLTAMETRİK HİDROJEN PEROKSİT BİYOSENSÖRÜYLE ERKEN HÜCRESEL STRES TESPİTİNİN ARAŞTIRILMASI	BİYOLOJİ	Biyomedikal Cihaz Teknolojileri	BURSA	BÖLGE BİRİNCİLİĞİ
88	1689B012435223	G**** Ü***	M***** B*******	YALOVA YALOVA-TERMAL FEN LİSESİ	ORTAOKUL BİYOLOJİ KONULARINI PEKİŞTİREN OYUN	BİYOLOJİ	Oyun ve Oyunlaştırma	BURSA	BÖLGE İKİNCİLİĞİ
89	1689B012430804	O*** K*** C*****<br>A*** G*****	S**** Ö****	ÇANAKKALE ÖZEL İSTEK ÇANAKKALE BİNDOKUZYÜZONBEŞ FEN LİSESİ	ÜFLEMELİ EĞİRME YÖNTEMİYLE ÜRETİLEN KARVAKROL İÇERİKLİ NANOLİFLERLE UZUN RAF ÖMÜRLÜ TAVUK ETİ	BİYOLOJİ	Gıda ve Gıda Arzı Güvenliği	BURSA	BÖLGE ÜÇÜNCÜLÜĞÜ
90	1689B012428493	E****** A******<br>D**** U***<br>E** D*********	H**** K********	BALIKESİR BANDIRMA ANADOLU LİSESİ	YAPAY ZEKÂ DESTEKLİ KARACABEY LONGOZU DOĞA EĞİTİM MERKEZİNİN OLUŞTURULMASI VE BİYOÇEŞİTLİLİK EĞİTİMİNE KOLAYLAŞTIRICI ETKİSİ	COĞRAFYA	Biyoçeşitlilik	BURSA	BÖLGE BİRİNCİLİĞİ

SIRA NO	BAŞVURU NO	ÖĞRENCİ ADI SOYADI	DANIŞMAN ADI SOYADI	OKULU	PROJENİN ADI	ANA ALANI	TEMATİK ALANI	BÖLGESİ	BÖLGE AŞAMASI SONUCU
91	1689B012417906	H**** B**** S****<br>E**** A*******<br>T**** F***** C*****	M****** S****	BURSA BTSO ALİ OSMAN SÖNMEZ SOSYAL BİLİMLER LİSESİ	3D BASKI VE PLA FİLAMENT ATIKLARININ TARIMDA GÜBRE OLARAK KULLANILABİLİRLİĞİ VE BİTKİLERİN GELİŞİMİNE ETKİSİ	COĞRAFYA	Atık Yönetimi ve Geri Dönüşüm	BURSA	BÖLGE İKİNCİLİĞİ
92	1689B012422844	S*** Ö*******<br>Z***** T****	I*** E******	ESKİŞEHİR ESKİŞEHİR ETİ SOSYAL BİLİMLER LİSESİ	YAPAY ZEKANIN GÖZÜNDEN METAFORLARIM, İKLİM MÜZESİNE DÖNÜŞTÜ	COĞRAFYA	Artırılmış, Sanal ve Karma Gerçeklik	BURSA	BÖLGE ÜÇÜNCÜLÜĞÜ
93	1689B012422829	E*** G**********<br>B**** Ş******<br>D*** Ü***	A*** Ö*****	BURSA BTSO ALİ OSMAN SÖNMEZ SOSYAL BİLİMLER LİSESİ	ÜÇ BOYUTLU MODELLEME VE YAZDIRMA TEKNOLOJİLERİ İLE KİLİM MOTİFLERİNİN GENÇ NESİLE AKTARILMASI: GELENEKTEN GELECEĞE MOTİF3D	DEĞERLER EĞİTİMİ	Kültürel Miras	BURSA	BÖLGE BİRİNCİLİĞİ
94	1689B012402747	C**** Ç*****<br>Y***** S***<br>N*** H****	F**** B*****	ESKİŞEHİR EMİNE-EMİR ŞAHBAZ BİLİM VE SANAT MERKEZİ	FİLMİ İZLE HİKAYENİ YAZ (WATCH THE MOVIE WRITE YOUR STORY)	DEĞERLER EĞİTİMİ	Yabancı Dil Eğitimi	BURSA	BÖLGE İKİNCİLİĞİ
95	1689B012435738	S*** Y****	P**** Y*****	BURSA HALİL İNALCIK SOSYAL BİLİMLER LİSESİ	KIRAATANNE: DİPSİZGÖL'DE YILDA BİR GÜN ROL-MEKAN DEĞİŞİMİ	DEĞERLER EĞİTİMİ	Değerler Eğitimi	BURSA	BÖLGE ÜÇÜNCÜLÜĞÜ
96	1689B012414018	T**** K***<br>A** D**** K********<br>İ*** Y*****	N***** S**** Ö*****	BALIKESİR ŞEHİT TURGUT SOLAK FEN LİSESİ	ÜLEKSİT, KOLEMANİT, KLİNOPTİLOİT ZEOLİTLİ SIVALARIN RADYOAKTİF IŞINIM SOĞURUMU, BASINÇ MUKAVAMETİ, ISI YALITIMI VE ANTİBAKTERİYEL ÖZELLİKLERİNİN KARŞILAŞTIRILMASI	FİZİK	Malzeme Bilimi ve Nanoteknoloji	BURSA	BÖLGE BİRİNCİLİĞİ
97	1689B012419489	U*** Ş****	A*** Y**** Ç****	ESKİŞEHİR ESKİŞEHİR FATİH FEN LİSESİ	FARKLI KONFİGÜRASYONLARDA DBD PLAZMA REAKTÖRLERİNİN GELİŞTİRİLEREK MEDİKAL ONKOLOJİ UYGULAMALARINDA ROS-RNS OLUŞTURAN BAROMETRİK NON-MAXWELLIAN PLAZMALARIN ÜRETİLMESİ	FİZİK	Biyomedikal Cihaz Teknolojileri	BURSA	BÖLGE BİRİNCİLİĞİ
98	1689B012433990	İ*** D*** A******<br>H**** İ**** T******	E** K*** B****	BALIKESİR ÖZEL BALIKESİR AÇI FEN LİSESİ<br>BALIKESİR ALTIEYLÜL FEN LİSESİ	DÜNYA'NIN MANYETİK ALANINDA OLUŞAN ANOMALİLERDEN FAYDALANILARAK EL YAPIMI PATLAYICILARIN UZAKTAN KONTROL İLE TESPİTİNİN ARAŞTIRILMASI	FİZİK	Milli Teknoloji Hamlesi	BURSA	BÖLGE İKİNCİLİĞİ
99	1689B012416350	N**** S****	H**** Ö*******	BURSA ÖZEL ŞAHİNKAYA FEN LİSESİ	SOLAR ENERJİDEN DAHA FAZLA VERİM; ZEYTİN YAPRAĞIYLA BİYOUYUMLU SÜPERKAPASİTÖRÜN KARAKTERİZASYONU	FİZİK	Yenilenebilir Enerji	BURSA	BÖLGE ÜÇÜNCÜLÜĞÜ
100	1689B012409428	A**** S*****<br>A**** K**** Y*****<br>O*** E** S****	S**** A*****	ESKİŞEHİR ÖZEL ESKİŞEHİR BAHÇEŞEHİR FEN VE TEKNOLOJİ LİSESİ	ELEKTRİKLİ ARABALAR İLACA DÖNÜŞECEK	KİMYA	Atık Yönetimi ve Geri Dönüşüm	BURSA	BÖLGE BİRİNCİLİĞİ
101	1689B012430846	E**** H******* S*****<br>Z***** N** E****	P**** S****	BALIKESİR ŞEHİT PROF. DR. İLHAN VARANK BİLİM VE SANAT MERKEZİ	YEŞİL SENTEZLE AZOT VE KÜKÜRT KATKILI KARBON KUANTUM NOKTALARININ SENTEZİ VE YÜKSEK HASSASİYETLİ DEMİR BİYOSENSÖRÜ HAZIRLANMASI	KİMYA	Biyomedikal Cihaz Teknolojileri	BURSA	BÖLGE İKİNCİLİĞİ
102	1689B012420634	D**** B****<br>C**** G*****	M**** Ç*****	ÇANAKKALE ÖZEL İSTEK ÇANAKKALE BİNDOKUZYÜZONBEŞ FEN LİSESİ	AKILLI ETİKET İLE BEYAZ ET KESİM TARİHİ VE BOZULMA TESPİTİ	KİMYA	Gıda ve Gıda Arzı Güvenliği	BURSA	BÖLGE ÜÇÜNCÜLÜĞÜ
103	1689B012420115	İ****** D**** A***<br>E*** E******<br>D**** Y*****	H**** Ç******	BALIKESİR ÖZEL BİLNET FEN LİSESİ	DİOPHANTİNE DENKLEMLERİ YARDIMIYLA YENİ BÖLÜNEBİLME ALGORİTMALARI OLUŞTURMA	MATEMATİK	Özgün Algoritma Tasarımı	BURSA	BÖLGE BİRİNCİLİĞİ
104	1689B012434802	A***** H****<br>Z**** S* U***<br>M***** U*** A**	A**** D*****	BURSA TED BURSA ÖZEL KOLEJİ	SALGINLARIN "COVID-19" VE "KIZIL" ÖRNEKLERİ ÜZERİNDEN YENİLİKÇİ MATEMATİKSEL YAKLAŞIMLARLA MODELLENMESİ VE "SİMÜLA-TÜRK" SİMÜLASYON PROTOTİPİNİN GELİŞTİRİLMESİ	MATEMATİK	Salgın Hastalıklar ve Salgınla Mücadele	BURSA	BÖLGE İKİNCİLİĞİ
105	1689B012437903	S**** K**** Ç****<br>M***** İ***		KÜTAHYA AYSEL-SELAHATTİN ERKASAP SOSYAL BİLİMLER LİSESİ<br>KÜTAHYA KÜTAHYA NAFİ GÜRAL FEN LİSESİ	GAUSS ÇEMBER PROBLEMİNİN PARABOL KESİTİNE UYGULANMASI	MATEMATİK	STEAM (Fen, Teknoloji, Mühendislik, Sanat ve Matematik)	BURSA	BÖLGE ÜÇÜNCÜLÜĞÜ
106	1689B012420407	A****** B******<br>E**** K******	Z***** Y***** B****	BALIKESİR BANDIRMA YAVUZ SULTAN SELİM ANADOLU LİSESİ	YENİ BİR TREND DİJİTAL DETOKS: LİSE ÖĞRENCİLERİNİN DİJİTAL BAĞIMLILIK VE AKADEMİK ERTELEME DÜZEYİNE ETKİSİ	PSİKOLOJİ	Bağımlılık ve Bağımlılıkla Mücadele	BURSA	BÖLGE BİRİNCİLİĞİ
107	1689B012412338	E*** Ü*** Ç****<br>Z***** E**** A****	Ş***** A******	BİLECİK REFİK ARSLAN ÖZTÜRK FEN LİSESİ	İNFOGRAFİK TASARLAMA ETKİNLİKLERİ İLE ÖĞRENCİLERDE SKOLYOZ FARKINDALIĞININ GELİŞTİRİLMESİ	PSİKOLOJİ	Halk Sağlığı ve Koruyucu Sağlık Hizmetleri	BURSA	BÖLGE İKİNCİLİĞİ
108	1689B012436968	S**** N** Ç*****<br>A*** H****** Ş****<br>R***** K**** M*****	M**** Ç*****	KÜTAHYA TAVŞANLI KIZ ANADOLU İMAM HATİP LİSESİ<br>KÜTAHYA TAVŞANLI ARSLANBEY MESLEKİ VE TEKNİK ANADOLU LİSESİ	LİSE ÖĞRENCİLERİNDEKİ AKILLI TELEFON BAĞIMLILIĞINA KARŞI FARK(N)ET UYGULAMASI	PSİKOLOJİ	Bağımlılık ve Bağımlılıkla Mücadele	BURSA	BÖLGE ÜÇÜNCÜLÜĞÜ
109	1689B012420431	D**** D***<br>S****** M****<br>T**** B****	Z***** Y***** B****	BALIKESİR BANDIRMA YAVUZ SULTAN SELİM ANADOLU LİSESİ	YENİ BİR EBEVEYN DAVRANIŞI OLARAK SHARENTİNG: DİJİTAL DÜNYANIN PAYLAŞAN ANNELERİ	SOSYOLOJİ	Dijital Dönüşüm	BURSA	BÖLGE BİRİNCİLİĞİ
110	1689B012421849	B**** Ü****<br>R****** Ö*****	M***** A****	ESKİŞEHİR CEMAL MÜMTAZ SOSYAL BİLİMLER LİSESİ	ÇEVRİMİÇİ HABİTUS ÜZERİNDEN YAPILANDIRILAN BİR EBEVEYNLİK MODELİ OLAN SHARENTİNG EYLEMİNİN ÇOCUK MAHREMİYETİ BAĞLAMINDA ANALİZİ	SOSYOLOJİ	Dijital Dönüşüm	BURSA	BÖLGE İKİNCİLİĞİ
111	1689B012423870	A*** E**** T*****<br>Z***** K*****	Ş***** A******	BİLECİK REFİK ARSLAN ÖZTÜRK FEN LİSESİ	BAĞLAM TEMELLİ ÖĞRENME ETKİNLİKLERİNİN SÜRDÜRÜLEBİLİR KALKINMA KAPSAMINDA ÇEVRESEL FARKINDALIK ÜZERİNE ETKİSİ	SOSYOLOJİ	Çevre ve Çevreyi Koruma	BURSA	BÖLGE İKİNCİLİĞİ
112	1689B012424618	M******* K*****<br>D**** O*<br>A*** V***** V****	Y**** E*** A***	BALIKESİR İSTANBULLUOĞLU SOSYAL BİLİMLER LİSESİ	TÜRKİYE'DEKİ OKULLARDA CEP TELEFONU YASAĞI POLİTİKASINI SOSYOLOJİK BİR BAKIŞ AÇISIYLA İNCELEMEK: PAYDAŞLARLA NİTEL BİR ÇALIŞMA	SOSYOLOJİ	Bağımlılık ve Bağımlılıkla Mücadele	BURSA	BÖLGE ÜÇÜNCÜLÜĞÜ
113	1689B012402433	H**** Ö*******<br>İ**** Ö******<br>C***** M****	S***** E****	ESKİŞEHİR ATATÜRK LİSESİ	OSMANLI DEVLETİNDE TANZİMAT İLE BAŞLAYAN BATILILAŞMA HAREKETLERİNİN OSMANLI AİLE YAŞAMINA YANSIMASI:AHMET CEVDET PAŞA ÖRNEĞİ	TARİH	Kültürel Miras	BURSA	BÖLGE BİRİNCİLİĞİ
114	1689B012428306	O****** D*********<br>Y**** K******<br>S**** S*** Ö****	H**** Y*****	ESKİŞEHİR SABİHA GÖKÇEN MESLEKİ VE TEKNİK ANADOLU LİSESİ	İLK TÜRK YOLCU UÇAĞI NU.D.38 VE HAVACILIK MEŞALESİNİN İLK KIVILCIMI SELAHATTİN REŞİT ALAN	TARİH	Havacılık ve Uzay Bilimleri	BURSA	BÖLGE BİRİNCİLİĞİ
115	1689B012436132	C**** E*** A****	A***** K****	BURSA BTSO KAMİL TOLON BİLİM VE SANAT MERKEZİ	FOTOĞRAFIN TANIKLIĞI İLE BURSA İLİNDEKİ TARİHİ MEKâNLARDA DEĞİŞİM	TARİH	Kültürel Miras	BURSA	BÖLGE İKİNCİLİĞİ
116	1689B012417550	M**** C******	M***** A****	ESKİŞEHİR CEMAL MÜMTAZ SOSYAL BİLİMLER LİSESİ	HISTOBOT: İNFOGRAFİKLERLE DESTEKLENMİŞ CHATBOT İLE TARİH EĞİTİMİ	TARİH	Dijital Dönüşüm	BURSA	BÖLGE ÜÇÜNCÜLÜĞÜ
117	1689B012418886	E** K****<br>E** S****	H**** Ö*******	BURSA HALİL İNALCIK BİLİM VE SANAT MERKEZİ	YENİLİKÇİ DİKEY EKSENLİ FANIN TEORİK SİMÜLASYONLA VE PRATİK OPTİMİZASYONU	TEKNOLOJİK TASARIM	STEAM (Fen, Teknoloji, Mühendislik, Sanat ve Matematik)	BURSA	BÖLGE BİRİNCİLİĞİ
118	1689B012424617	A*** T****<br>E**** K********	S***** C****	BALIKESİR GÖNEN ÖMER SEYFETTİN FEN LİSESİ	IŞIK ŞAMANDIRALARI: AFET ALANLARI İÇİN MOBİL AYDINLATMA İNOVASYONU	TEKNOLOJİK TASARIM	Doğal Afetler ve Afet Yönetimi	BURSA	BÖLGE İKİNCİLİĞİ
119	1689B012409495	S* H**** U***	G**** F*****	BURSA ÖZEL ÇAĞDAŞ ÖNCÜ FEN LİSESİ	SESLERİN YÖNÜNÜ FARK EDEBİLİYORUM	TEKNOLOJİK TASARIM	Giyilebilir Teknolojiler	BURSA	BÖLGE ÜÇÜNCÜLÜĞÜ
120	1689B012431031	Y**** E******<br>Z***** T*******	Ö**** S****	KÜTAHYA KÜTAHYA BİLİM VE SANAT MERKEZİ	TÜRK MİTOLOJİSİNİN ÖĞRETİMİNDE OYUNLAŞTIRMA VE MİKA (MİTOLOJİNİN KAĞANLARI) OYUNU	TÜRK DİLİ VE EDEBİYATI	Oyun ve Oyunlaştırma	BURSA	BÖLGE BİRİNCİLİĞİ
121	1689B012413962	E*** T****<br>M**** T*** A******<br>G**** G**** K***	S***** S****	ESKİŞEHİR ÖZEL ŞEHİR KOLEJİ FEN LİSESİ	GEÇMİŞTEN GELEN SESE KULAK VER (DİJİTAL DÖNÜŞÜM TEKNOLOJİLERİYLE TASARLANMIŞ ŞAİRLER DERGİSİ)	TÜRK DİLİ VE EDEBİYATI	Dijital Dönüşüm	BURSA	BÖLGE İKİNCİLİĞİ

SIRA NO	BAŞVURU NO	ÖĞRENCİ ADI SOYADI	DANIŞMAN ADI SOYADI	OKULU	PROJENİN ADI	ANA ALANI	TEMATİK ALANI	BÖLGESİ	BÖLGE AŞAMASI SONUCU
122	1689B012421420	A*** M**** D*****<br>İ*** A****	Y**** E*** A***	BALIKESİR İSTANBULLUOĞLU SOSYAL BİLİMLER LİSESİ	3D SANAL GERÇEKLİK ÖĞRENME ORTAMINDA GERÇEKLEŞTİRİLEN UZAKTAN EĞİTİMİN ANADİLDE KONUŞMA BECERİLERİ VE KONUŞMA KAYGILARI ÜZERİNE ETKİSİ	TÜRK DİLİ VE EDEBİYATI	Uzaktan Eğitim	BURSA	BÖLGE ÜÇÜNCÜLÜĞÜ
123	1689B012418881	M******* F**** A***	H**** Ö*******	BURSA HALİL İNALCIK BİLİM VE SANAT MERKEZİ	OSTEOARTRİT HASTALARININ REHABİLİTASYONU İÇİN YAPAY ZEKâ DESTEKLİ FİZYOTERAPİ TAKİP ASİSTANI GELİŞTİRİLMESİ; DİZ-KALÇA EKLEMİ ÖRNEĞİ	YAZILIM	Yapay Zekâ	BURSA	BÖLGE BİRİNCİLİĞİ
124	1689B012428800	A** E*** Ö*****	H**** S****	BURSA ALİ OSMAN SÖNMEZ MESLEKİ VE TEKNİK ANADOLU LİSESİ	MOTOR NÖRON HASTALARININ ÇEVRELERİ İLE İLETİŞİMİNİ SAĞLAYAN BEYİN BİLGİSAYAR ARAYÜZÜ DESTEKLİ MOBİL UYGULAMA; CAN DOSTUM	YAZILIM	Biyomedikal Cihaz Teknolojileri	BURSA	BÖLGE İKİNCİLİĞİ
125	1689B012417470	B*** T*** Ö****<br>D*** T*****<br>H**** B**** Ç*******	M**** Ö****	ÇANAKKALE BİGA İÇDAŞ FEN LİSESİ	YİTU İLE HER ZAMAN GÜVENDEYİM	YAZILIM	Yapay Zekâ	BURSA	BÖLGE ÜÇÜNCÜLÜĞÜ
126	1689B012407285	E*** D****<br>M******* E****	T**** A****	ERZİNCAN İPEKYOLU MESLEKİ VE TEKNİK ANADOLU LİSESİ	CİSPLATİN/YEŞİL SENTEZ GÜMÜŞ NANOPARTİKÜLLERİ KOMBİNASYONUNUN ANTİKANSER VE ANTİOKSİDAN ETKİNLİKLERİNİN ARAŞTIRILMASI	BİYOLOJİ	Malzeme Bilimi ve Nanoteknoloji	ERZURUM	BÖLGE BİRİNCİLİĞİ
127	1689B012429500	A*** A***	A**** P**** K*****	ERZURUM REMZİ SAKAOĞLU BİLİM VE SANAT MERKEZİ	ZNO NANOPARTİKÜLLERİNİN ROBİNİA PSEUDOACACİA (YALANCI AKASYA) YARDIMLI YEŞİL SENTEZİ VE BİYOGÜVENLİĞİNİN ARAŞTIRILMASI	BİYOLOJİ	Malzeme Bilimi ve Nanoteknoloji	ERZURUM	BÖLGE BİRİNCİLİĞİ
128	1689B012430257	D**** A******<br>A** E** A****	F**** G*****	TRABZON ORTAHİSAR BİLİM VE SANAT MERKEZİ	FEROKAP (AKILLI FEROMON TUZAĞI)	BİYOLOJİ	Ekolojik Denge	ERZURUM	BÖLGE İKİNCİLİĞİ
129	1689B012429326	O*** A****	E**** Y*******	KARS PROF. DR. FAHRETTİN KIRZIOĞLU BİLİM VE SANAT MERKEZİ	HUŞUYORUM	BİYOLOJİ	Tarım ve Hayvancılık Teknolojileri	ERZURUM	BÖLGE ÜÇÜNCÜLÜĞÜ
130	1689B012422274	D**** Y*****	H***** V********* K*****	ARDAHAN GÖLE BEDİRHAN BEY ANADOLU İMAM HATİP LİSESİ	ARDAHAN GÖLE İLÇESİNE BAĞLI KÖYLERİN ALTERNATİF TURİZM POTANSİYELİNİN BELİRLENMESİ	COĞRAFYA	Sürdürülebilir Şehirler ve Toplumlar	ERZURUM	BÖLGE BİRİNCİLİĞİ
131	1689B012423767	S*** Ş****<br>V**** K******	T**** A*****	TRABZON AKÇAABAT BORSA İSTANBUL ANADOLU LİSESİ<br>TRABZON AKÇAABAT FEN LİSESİ	OKUL DIŞI ÖĞRENME ORTAMLARINDA AFET EĞİTİMİ	COĞRAFYA	Doğal Afetler ve Afet Yönetimi	ERZURUM	BÖLGE İKİNCİLİĞİ
132	1689B012435210	Ş***** K*****<br>M**** Y*****<br>N***** V*** İ***	V***** K*******	TRABZON TRABZON SOSYAL BİLİMLER LİSESİ	YAPAY ZEKÂ DESTEKLİ SANAL REHBER UYGULAMASI	COĞRAFYA	Yapay Zekâ	ERZURUM	BÖLGE ÜÇÜNCÜLÜĞÜ
133	1689B012433471	E**** C*****<br>Z**** T******<br>E** C*****	F**** Ş****	TRABZON AKÇAABAT FEN LİSESİ	FİDA-GERİ DÖNÜŞÜMLE OLUŞAN, EL SANATLARI İLE COŞAN, MAHARETTEN MARİFETE UZANAN BİR İYİLİK YOLCULUĞU	DEĞERLER EĞİTİMİ	Değerler Eğitimi	ERZURUM	BÖLGE BİRİNCİLİĞİ
134	1689B012437666	M***** B**** U*****<br>C**** P****	E***** O******	KARS PROF. DR. FAHRETTİN KIRZIOĞLU BİLİM VE SANAT MERKEZİ<br>KARS KARS FEN LİSESİ	"NAKIŞ, GEÇMİŞE FARKLI BİR BAKIŞ"	DEĞERLER EĞİTİMİ	Kültürel Miras	ERZURUM	BÖLGE BİRİNCİLİĞİ
135	1689B012421175	A****** A******<br>S*** H********<br>A**** H**** K****	H**** A***** C**	TRABZON SAFFET ÇEBİ MESLEKİ VE TEKNİK ANADOLU LİSESİ	DİJİTALİ AKIL EDEREK KULLAN, HAYATI FARK EDEREK YAŞA	DEĞERLER EĞİTİMİ	Bağımlılık ve Bağımlılıkla Mücadele	ERZURUM	BÖLGE İKİNCİLİĞİ
136	1689B012411218	Y***** Ş**********	H**** Ü*****	BAYBURT MUSTAFA KÖSEOĞLU ÇOK PROGRAMLI ANADOLU LİSESİ	TEKNOAT İLE GELECEĞE AT	DEĞERLER EĞİTİMİ	Atık Yönetimi ve Geri Dönüşüm	ERZURUM	BÖLGE ÜÇÜNCÜLÜĞÜ
137	1689B012415074	M****** İ**** P*****	M****** A***	ERZURUM REMZİ SAKAOĞLU BİLİM VE SANAT MERKEZİ	VİDALI KRİKO SİSTEMİ İLE TASARLANAN AKUSTİK LEVİTASYON CİHAZI İLE LOKAL KAVİTASYON YAPIMI	FİZİK	Biyomedikal Cihaz Teknolojileri	ERZURUM	BÖLGE BİRİNCİLİĞİ
138	1689B012423378	M******* E*** Ç****<br>T*** R**** A****<br>B***** L****	E**** P*****	TRABZON TRABZON FARUK BAŞARAN BİLİM VE SANAT MERKEZİ	SÜRDÜRÜLEBİLİR ENERJİ İÇİN YENİLİKÇİ TÜRBİN OPTİMİZASYONU	FİZİK	Yenilenebilir Enerji	ERZURUM	BÖLGE İKİNCİLİĞİ
139	1689B012428845	Y**** E*** Ş*****<br>Y**** Y*******<br>U*** T**** T******	L**** K***	TRABZON TRABZON FARUK BAŞARAN BİLİM VE SANAT MERKEZİ	YÜKSEK YÜZEY ÇÖZÜNÜRLÜKLÜ UYGUN MALİYETLİ OTONOM RADYASYON ERKEN UYARI SİSTEMİ: TRABZON ÖRNEKLEMİ	FİZİK	STEAM (Fen, Teknoloji, Mühendislik, Sanat ve Matematik)	ERZURUM	BÖLGE ÜÇÜNCÜLÜĞÜ
140	1689B012401575	S****** Ş********<br>N***** K***<br>İ**** Ö***	D**** K**	ERZURUM TEKMAN ANADOLU LİSESİ	PERİYODİK TABLO OYUNU: PERİYOPYA	KİMYA	Oyun ve Oyunlaştırma	ERZURUM	BÖLGE BİRİNCİLİĞİ
141	1689B012436858	M**** N**** D*******<br>A**** S*** Ç******	Ç***** U*****	IĞDIR HAYDAR ALİYEV FEN LİSESİ	ENZİMATİK BOYAMA İLE TEMİZ TEKSTİL ÜRETİMİ	KİMYA	Çevre ve Çevreyi Koruma	ERZURUM	BÖLGE İKİNCİLİĞİ
142	1689B012428714	E** N** A***<br>F******* K********	B**** G****	BAYBURT DEMİRÖZÜ GÖKÇEDERE ÇOK PROGRAMLI ANADOLU LİSESİ	SU YAŞAM KAYNAĞIMIZDIR	KİMYA	Sorumlu Üretim ve Tüketim	ERZURUM	BÖLGE ÜÇÜNCÜLÜĞÜ
143	1689B012416258	M****** Y**** Ü*******	O*** S***** U******	RİZE RİZE TÜRKİYE ODALAR VE BORSALAR BİRLİĞİ FEN LİSESİ	ÇEVRESİ SABİT TAM SAYI ÜÇGENLERİNİN SAYISINI BULAN ALGORİTMA VE HONSBERGER TEOREMİ İÇİN YENİ BİR İSPAT	MATEMATİK	Özgün Algoritma Tasarımı	ERZURUM	BÖLGE BİRİNCİLİĞİ
144	1689B012418420	İ**** S********	M****** A***	ERZURUM REMZİ SAKAOĞLU BİLİM VE SANAT MERKEZİ	SİERPİNSKİ FRAKTALLARINDAN TASARLANAN ALTIGENLER OYUNUNUN PASCAL-HAYYAM ÜÇGENİ VE KUMMER TEOREMİ İLE KANITLANMASI	MATEMATİK	Özgün Algoritma Tasarımı	ERZURUM	BÖLGE İKİNCİLİĞİ
145	1689B012411233	B****** A*****<br>A** T**** B*****<br>G***** B*****	F**** A****	TRABZON TRABZON FARUK BAŞARAN BİLİM VE SANAT MERKEZİ	FOTOĞRAFIN GİZEMLİ ŞİFRESİ	MATEMATİK	Özgün Algoritma Tasarımı	ERZURUM	BÖLGE ÜÇÜNCÜLÜĞÜ
146	1689B012430189	İ*** G******* B****<br>D***** K******	A**** Ö******	TRABZON TRABZON SOSYAL BİLİMLER LİSESİ	BOŞANMIŞ AİLELERİN BOŞANMA SÜRECİNDE YAŞADIKLARININ ÇOCUKLARININ PSİKOLOJİSİNE ETKİSİ	PSİKOLOJİ	Aile İçi İletişim	ERZURUM	BÖLGE BİRİNCİLİĞİ
147	1689B012433834	N******* K*******<br>İ*** S******	A***** B****	TRABZON TRABZON SOSYAL BİLİMLER LİSESİ	ANİMELERİN ORTAÖĞRETİM ÖĞRENCİLERİNİN DAVRANIŞLARI ÜZERİNDE MEYDANA GETİRDİĞİ DEĞİŞİKLİKLER	PSİKOLOJİ	Medya Okuryazarlığı	ERZURUM	BÖLGE İKİNCİLİĞİ
148	1689B012412131	S*** D****<br>M***** B****	N***** G******	ERZİNCAN ATATÜRK MESLEKİ VE TEKNİK ANADOLU LİSESİ	ERZİNCAN VALİLİĞİ İNİSİYATİFİYLE YÜRÜTÜLEN MENTÖRÜM SENSİN PROJESİ'NDEKİ DEZAVANTAJLI MAHALLELERDEKİ OKULUMUZ ÖĞRENCİLERİNİN SORUMLULUK DUYGUSUNA VE DAVRANIŞINA ETKİSİ"	PSİKOLOJİ	Değerler Eğitimi	ERZURUM	BÖLGE ÜÇÜNCÜLÜĞÜ
149	1689B012421524	İ***** B*****<br>Ö*** S**** G**<br>F**** D***** T****	V***** Ş**	ERZURUM TEVFİK İLERİ ANADOLU LİSESİ	THE KALİK LOSTA EĞİTİM PROGRAMLARI İLE ATIK YÖNETİMİ	SOSYOLOJİ	Sürdürülebilir Şehirler ve Toplumlar	ERZURUM	BÖLGE BİRİNCİLİĞİ
150	1689B012422594	B***** A****<br>R**** Ş****	T**** T****	ERZURUM TEKMAN ANADOLU İMAM HATİP LİSESİ	AİLE İÇİ İLETİŞİMİ OLUMLU YÖNDE ETKİLEYECEK OYUNLAR	SOSYOLOJİ	Aile İçi İletişim	ERZURUM	BÖLGE BİRİNCİLİĞİ
151	1689B012405946	N**** U*******<br>Z**** E**** F****<br>E*** M****	A**** A*******	RİZE GÜNEYSU ŞEHİT KEMAL MUTLU FEN LİSESİ	BİLİŞİM ÇAĞINDA TEMEL BİR İNSAN HAKKI OLARAK UNUTULMA HAKKI	SOSYOLOJİ	Siber Güvenlik	ERZURUM	BÖLGE İKİNCİLİĞİ
152	1689B012419023	E****** S********<br>M******* Ç****<br>E***** Ö****	N***** B****	GÜMÜŞHANE GÜMÜŞHANE İBN-İ SİNA MESLEKİ VE TEKNİK ANADOLU LİSESİ	GENÇ ETİK	SOSYOLOJİ	Medya Okuryazarlığı	ERZURUM	BÖLGE ÜÇÜNCÜLÜĞÜ

SIRA NO	BAŞVURU NO	ÖĞRENCİ ADI SOYADI	DANIŞMAN ADI SOYADI	OKULU	PROJENİN ADI	ANA ALANI	TEMATİK ALANI	BÖLGESİ	BÖLGE AŞAMASI SONUCU
153	1689B012434541	Z***** E*** Y****<br>F**** A*****<br>Z**** G****** Y********	M****** Ö*****	TRABZON TRABZON MERKEZ FEN LİSESİ	BİR MOBİL ÖĞRENME UYGULAMASI "DİJİTAL TRABZON MAARİF TAKVİMİ"	TARİH	Kültürel Miras	ERZURUM	BÖLGE BİRİNCİLİĞİ
154	1689B012421169	Ö*** C** Y*******<br>M***** A** A***	H**** S****	TRABZON SAFFET ÇEBİ MESLEKİ VE TEKNİK ANADOLU LİSESİ	KONUŞAN TARİH	TARİH	Yapay Zekâ	ERZURUM	BÖLGE İKİNCİLİĞİ
155	1689B012427235	A*** A*******<br>S**** N*** T**	C********* B***	TRABZON TRABZON YOMRA FEN LİSESİ	OKULLAR YOKKEN OYUNLAR VARDI	TARİH	Oyun ve Oyunlaştırma	ERZURUM	BÖLGE ÜÇÜNCÜLÜĞÜ
156	1689B012433022	C** A**** K***<br>Y**** E*** F****	V***** D*******	ERZURUM ŞÜKRÜPAŞA ANADOLU LİSESİ	TRAFİK KAZASI TESPİT VE BİLDİRİ SİSTEMİ	TEKNOLOJİK TASARIM	Yapay Zekâ	ERZURUM	BÖLGE BİRİNCİLİĞİ
157	1689B012430089	E** K**** İ***<br>A**** Y**** B******	O*** E*****	ERZİNCAN ERZİNCAN BİLİM VE SANAT MERKEZİ	SU OKURYAZARI KOMBİ SİSTEMİ	TEKNOLOJİK TASARIM	Su Okuryazarlığı	ERZURUM	BÖLGE İKİNCİLİĞİ
158	1689B012421605	H**** S*****<br>M******* A*** G****	T***** B***** B****	ERZURUM ERZURUM LİSESİ	KOÇ AI	TEKNOLOJİK TASARIM	Yapay Zekâ	ERZURUM	BÖLGE ÜÇÜNCÜLÜĞÜ
159	1689B012412141	Z***** Y*******<br>Y**** H**** K*******	F***** E******	BAYBURT BAYBURT LİSESİ	ÖZ TÜRKÇE	TÜRK DİLİ VE EDEBİYATI	Türk Dili ve Lehçeleri	ERZURUM	BÖLGE BİRİNCİLİĞİ
160	1689B012413748	A*** E**** Ö*****<br>A*** İ*** K********	İ**** S****	ERZİNCAN ERZİNCAN BİLİM VE SANAT MERKEZİ	BİLKOT (BİLDİRİMLERLE KONU TEKRAR UYGULAMASI)	TÜRK DİLİ VE EDEBİYATI	Robotik ve Kodlama	ERZURUM	BÖLGE BİRİNCİLİĞİ
161	1689B012433059	M***** Ş*******	S*** K******	RİZE PAZAR KIZ KULESİ MESLEKİ VE TEKNİK ANADOLU LİSESİ	YAZIMI KARIŞTIRILAN KELİMELERİN LABİRENT OYUNU İLE ÖĞRENİLMESİ	TÜRK DİLİ VE EDEBİYATI	Oyun ve Oyunlaştırma	ERZURUM	BÖLGE İKİNCİLİĞİ
162	1689B012404545	B**** N** Ş*****<br>R***** K******	S**** K*********	ERZURUM ATATÜRK ANADOLU LİSESİ	SANATTA DİSİPLİNLER ARASI BİR YAKLAŞIM: EDEBİYATTA ENSTALASYON SANATINDAN YARARLANILABİLİR Mİ?	TÜRK DİLİ VE EDEBİYATI	Görsel ve İşitsel Sanatlar	ERZURUM	BÖLGE ÜÇÜNCÜLÜĞÜ
163	1689B012415799	O**** B**** K**<br>A*** C**	S******* S*****	ARTVİN ERTUĞRUL KURDOĞLU FEN LİSESİ	LOCKED-IN SENDROMLU HASTALAR İÇİN ALTERNATİF İLETİŞİM PROGRAMI (ALS EYE TRACKER)	YAZILIM	Görüntü ve Ses Tanıma Teknolojileri	ERZURUM	BÖLGE BİRİNCİLİĞİ
164	1689B012424944	A**** K******	U*** C***	TRABZON GAZİ ANADOLU LİSESİ	NÜKLEER ENERJİ DİJİTAL OYUNU	YAZILIM	Dijital Oyun Tasarımı	ERZURUM	BÖLGE İKİNCİLİĞİ
165	1689B012436870	H***** Ö***<br>N*** A*** K******	S***** Y*****	RİZE FATMA-NURİ ERKAN BİLİM VE SANAT MERKEZİ<br>RİZE RİZE TÜRKİYE ODALAR VE BORSALAR BİRLİĞİ FEN LİSESİ	ACİL DURUM HAYVAN TAHLİYE SİSTEMİ (ADHTS)	YAZILIM	Doğal Afetler ve Afet Yönetimi	ERZURUM	BÖLGE ÜÇÜNCÜLÜĞÜ
166	1689B012428507	K*** B*** Ö**<br>E** N** Y****	S**** Y*****	İSTANBUL ÖZEL BİLFEN ÜSKÜDAR FEN LİSESİ	ATIK PLASTİKLERDEN LİPAZ HİDROLİZLİ MİKROPLASTİKLERİN OLUŞTURULMASI VE KARAKTERİZASYONU İLE ÜRÜNÜN NANOGÜMÜŞ KAPLI ANTİMİKROBİYAL HAMMADEYE DÖNÜŞTÜRÜLMESİ	BİYOLOJİ	Malzeme Bilimi ve Nanoteknoloji	İSTANBUL-ASYA	BÖLGE BİRİNCİLİĞİ
167	1689B012431291	E** A****<br>Z***** K*** T*****	B**** Y*******	İSTANBUL ÖZEL SANCAKTEPE OKYANUS ANADOLU LİSESİ	PREBİYOTİK İÇERİĞİ ZENGİNLEŞTİRİLMİŞ VE LAKTOBASİLLUS PLANTERUM İLE FERMENTE BİYOFİLMLERİN ÜRETİLMESİ	BİYOLOJİ	Genetik ve Biyoteknoloji	İSTANBUL-ASYA	BÖLGE BİRİNCİLİĞİ
168	1689B012403563	A**** F**** S******	P**** Ö******	İSTANBUL ARAŞTIRMA GELİŞTİRME EĞİTİM VE UYGULAMA MERKEZİ LİSESİ	CAMELLİA SİNENSİS VE COFFEA ARABİCA'NIN UYANDIRICI ETKİSİNİN MOLEKÜLER DÜZEYDE İNCELENMESİ	BİYOLOJİ	Genetik ve Biyoteknoloji	İSTANBUL-ASYA	BÖLGE İKİNCİLİĞİ
169	1689B012436864	Z**** Ç*****<br>Ş**** E** D*****	H**** E**	İSTANBUL KARTAL PROF. DR. ŞABAN TEOMAN DURALI BİLİM VE SANAT MERKEZİ	PSORİASİS TEDAVİSİNDE YENİLİKÇİ BİR YAKLAŞIM: DRACUNCULUS VULGARİS BİTKİ ÖZÜTÜNÜN KARAKTERİZASYONU VE ETKİ MEKANİZMASININ ARAŞTIRILMASI	BİYOLOJİ	Genetik ve Biyoteknoloji	İSTANBUL-ASYA	BÖLGE ÜÇÜNCÜLÜĞÜ
170	1689B012411897	E**** D****<br>M*** Ü****	Y**** K*****	İSTANBUL ÖZEL KÜÇÜK PRENS LİSESİ	TAHILLARIN DEPOLANMASINDA ÖZGÜN YÖNTEM: SCUTOİD DOKULU MODEL SİLO TASARIMI İLE SİLO YÖNETİM SİSTEMİ (SYS)	COĞRAFYA	STEAM (Fen, Teknoloji, Mühendislik, Sanat ve Matematik)	İSTANBUL-ASYA	BÖLGE BİRİNCİLİĞİ
171	1689B012404568	E** Ş****<br>S*** K************<br>D*** B*****	Y**** K*****	İSTANBUL ÖZEL KÜÇÜK PRENS LİSESİ	TARIM 4.0 KAPSAMINDA İHA VE HAVA İSTASYONUYLA ORTOFOTO HARİTA VE METEOROLOJİK VERİ ÜRETİMİ: ZİC-İ ULUĞ SİSTEMİ	COĞRAFYA	Havacılık ve Uzay Bilimleri	İSTANBUL-ASYA	BÖLGE İKİNCİLİĞİ
172	1689B012429757	Z**** T****<br>R***** E**** D****	H***** D****	SAKARYA SAKARYA ÜNİVERSİTESİ VAKFI ÖZEL ANADOLU LİSESİ	AKILLI ŞEHİR YÖNETİMİNDE WEB TABANLI COĞRAFİ BİLGİ SİSTEMLERİ KULLANIMI	COĞRAFYA	Dijital Dönüşüm	İSTANBUL-ASYA	BÖLGE ÜÇÜNCÜLÜĞÜ
173	1689B012419316	E*** B**** E***<br>D**** G**<br>A*** B***** E***	V***** A******** K****	KOCAELİ ÖZEL SINAV KOLEJİ ŞAHİN FEN LİSESİ	LİSE ÖĞRENCİLERİNİN GAZZE'DE YAŞANAN ÇATIŞMALARA İLİŞKİN METAFORİK ALGILARI	DEĞERLER EĞİTİMİ	Değerler Eğitimi	İSTANBUL-ASYA	BÖLGE BİRİNCİLİĞİ
174	1689B012433764	M****** E** Ç****<br>E*** T*****	G**** A***	İSTANBUL ÖZEL ANABİLİM FEN VE TEKNOLOJİ LİSESİ<br>İSTANBUL ÖZEL ANABİLİM ANADOLU LİSESİ	GEÇMİŞTEN GÜNÜMÜZE TEMİZLİK DEĞERİNİN ÇEVRE BİLİNCİNE ETKİSİ ÜZERİNE BİR ÇALIŞMA: OSMANLI'DAN GELEN TEMİZLİK	DEĞERLER EĞİTİMİ	Dijital Dönüşüm	İSTANBUL-ASYA	BÖLGE İKİNCİLİĞİ
175	1689B012428288	T*** D*********<br>E**** E** O******<br>Y**** E** Ç****	B**** Ö****	SAKARYA ŞEHİT ÜSTEĞMEN İBRAHİM ABANOZ ANADOLU LİSESİ	NESİLLER ARASI ETKİLEŞİM UYGULAMALARININ LİSE ÖĞRENCİLERİNİN YAŞLI AYRIMCILIĞINA YÖNELİK TUTUMUNA ETKİSİ	DEĞERLER EĞİTİMİ	Yaşamımızda İyilik, Nezaket ve Anlayış	İSTANBUL-ASYA	BÖLGE ÜÇÜNCÜLÜĞÜ
176	1689B012425557	İ**** K*** T*******<br>G***** E*** B*******<br>O**** K*** E***	Ö*** Y***** Y********	İSTANBUL İSTEK ÖZEL BELDE ANADOLU LİSESİ<br>İSTANBUL ÖZEL TED RÖNESANS ANADOLU LİSESİ	YENİ NESİL MANYETİK HIZLANDIRICI İLE KIZILELMA İNSANSIZ SAVAŞ UÇAĞININ UÇUŞ SÜRESİNİN %16 ARTTIRILMASI	FİZİK	Havacılık ve Uzay Bilimleri	İSTANBUL-ASYA	BÖLGE BİRİNCİLİĞİ
177	1689B012435529	K**** G****<br>E*** K*******<br>D*** Ç**** O****	M***** D*****	İSTANBUL İSTANBUL ATATÜRK FEN LİSESİ	NİKOFF: DİFÜZYON UZUNLUĞU SINIRLAMA MODELİYLE NİKOTİNİN KONTROLLÜ FİLTRELENMESİ VE SİGARA BAĞIMLILIĞINA YENİ BİR ÇÖZÜM	FİZİK	Bağımlılık ve Bağımlılıkla Mücadele	İSTANBUL-ASYA	BÖLGE BİRİNCİLİĞİ
178	1689B012432891	B****** B*****<br>A**** G****<br>S**** E**** K*****	M**** P**** T****	İSTANBUL ÖZEL ANABİLİM ANADOLU LİSESİ<br>İSTANBUL ÖZEL ANABİLİM FEN VE TEKNOLOJİ LİSESİ	ALBÜMİN VE KARBON KUANTUM NOKTA İÇERİKLİ DOĞAL KÜL ÇİMENTO İLE FİBONACCİ YÜZEY TASARIMLI DALGAKIRAN KIYI PANELLERİ	FİZİK	Malzeme Bilimi ve Nanoteknoloji	İSTANBUL-ASYA	BÖLGE İKİNCİLİĞİ
179	1689B012408464	İ*** A*****<br>Z***** E** A***<br>E** M****	S**** Ö****	İSTANBUL İSTANBUL FUAT SEZGİN BİLİM VE SANAT MERKEZİ	BİTKİSEL MALZEMELERDEN KUANTUM NOKTALARI ELDE EDİLEREK KIRILMA İNDİSLERİNİN ARAŞTIRILMASI VE KUANTUM NOKTALI OPTİK CAM TASARIMI	FİZİK	STEAM (Fen, Teknoloji, Mühendislik, Sanat ve Matematik)	İSTANBUL-ASYA	BÖLGE ÜÇÜNCÜLÜĞÜ
180	1689B012405692	E*** B**** D****	S**** S*****	KOCAELİ TÜBİTAK FEN LİSESİ	FINDIK ZURUFU VE MAKROALGLERDEN DÖNGÜSEL EKONOMİYE KATKI SAĞLAYACAK BİYOPLASTİK GIDA AMBALAJI SENTEZİ VE KARAKTERİZASYONU	KİMYA	Malzeme Bilimi ve Nanoteknoloji	İSTANBUL-ASYA	BÖLGE BİRİNCİLİĞİ
181	1689B012406467	R***** Ç****	P**** D*****	İSTANBUL KARTAL ANADOLU İMAM HATİP LİSESİ	TOPİKAL AJANLA DESTEKLENMİŞ POTANSİYEL HÜCREDIŞI MATRİKS VE YARA ÖRTÜSÜ YENİ HİDROJEL SENTEZİ, KARAKTERİZASYONU, ETKİ MEKANİZMASININ İNCELENMESİ	KİMYA	Malzeme Bilimi ve Nanoteknoloji	İSTANBUL-ASYA	BÖLGE BİRİNCİLİĞİ
182	1689B012421709	Z***** D*****<br>Z***** B********	S**** B******	KOCAELİ ÖZEL ENKA MESLEKİ VE TEKNİK ANADOLU LİSESİ	GIDA ATIKLARININ DEĞERLENDİRİLEREK YEŞİL SENTEZLE OLUŞAN ÇİNKO OKSİT BİYONANOPARTİKÜLLERİN TEKSTİL ATIK SULARINDAKİ METİLEN MAVİSİNİ UZAKLAŞTIRMASI	KİMYA	Malzeme Bilimi ve Nanoteknoloji	İSTANBUL-ASYA	BÖLGE İKİNCİLİĞİ
183	1689B012438780	İ*** T****<br>B**** A*****	Ö**** İ** T****	İSTANBUL ÖZEL ANABİLİM FEN VE TEKNOLOJİ LİSESİ	İNCE FİLM KAPLAMALI KUANTUM NOKTA KATKILI SOLAR HÜCRE KARAKTERİZASYONU	KİMYA	Yenilenebilir Enerji	İSTANBUL-ASYA	BÖLGE ÜÇÜNCÜLÜĞÜ

SIRA NO	BAŞVURU NO	ÖĞRENCİ ADI SOYADI	DANIŞMAN ADI SOYADI	OKULU	PROJENİN ADI	ANA ALANI	TEMATİK ALANI	BÖLGESİ	BÖLGE AŞAMASI SONUCU
184	1689B012406477	O**** E*** D*****<br>A******* F****<br>A*** E*** K*****	P**** D*****	İSTANBUL KARTAL ANADOLU İMAM HATİP LİSESİ	YENİ VE ÖZGÜN BİR POST-KUANTUM HİBRİT KRİPTOSİSTEMİNİN GELİŞTİRİLMESİ VE UYGULANMASI	MATEMATİK	Özgün Algoritma Tasarımı	İSTANBUL-ASYA	BÖLGE BİRİNCİLİĞİ
185	1689B012438793	S*** Y**** Ç****<br>E**** D*** P****	F**** Y**** Ö*** AK***	İSTANBUL ALEV ALATLI BİLİM VE SANAT MERKEZİ	RECAMAN SAYI DİZİSİ İLE İMGESEL KRİPTOLOJİ ALGORİTMASI	MATEMATİK	Özgün Algoritma Tasarımı	İSTANBUL-ASYA	BÖLGE BİRİNCİLİĞİ
186	1689B012437788	A**** A** O*****<br>T*** O****<br>O**** B**** A******	T**** B**********	KOCAELİ TÜBİTAK FEN LİSESİ	DFS İLE MATRİS ŞİFRELEME	MATEMATİK	Özgün Algoritma Tasarımı	İSTANBUL-ASYA	BÖLGE İKİNCİLİĞİ
187	1689B012435971	M**** E**** K**	İ**** B*****	İSTANBUL ÖZEL BURÇAK EYÜBOĞLU FEN VE TEKNOLOJİ LİSESİ	ÇOKGENİN KÖŞELERİNDEN FARKLI ÜÇGENLERE	MATEMATİK	Özgün Algoritma Tasarımı	İSTANBUL-ASYA	BÖLGE ÜÇÜNCÜLÜĞÜ
188	1689B012426728	Z***** A****<br>C**** S*****<br>M***** B******	A**** T********	İSTANBUL KÖY HİZMETLERİ ANADOLU LİSESİ	ERGENLERİN DİJİTAL BAĞIMLILIKLARININ YORDANMASINDA AKADEMİK ATALET VE DÜRTÜSELLİĞİN ROLÜ	PSİKOLOJİ	Bağımlılık ve Bağımlılıkla Mücadele	İSTANBUL-ASYA	BÖLGE BİRİNCİLİĞİ
189	1689B012413162	T**** M**** Ş******<br>E*** M*****<br>E*** N** O****	S***** Z*****	SAKARYA KARASU ATATÜRK ANADOLU LİSESİ	HELİKOPTER EBEVEYNE SAHİP ERGENLERDE ALEKSİTİMİ	PSİKOLOJİ	Aile İçi İletişim	İSTANBUL-ASYA	BÖLGE İKİNCİLİĞİ
190	1689B012413764	T*** F***** B********<br>N*** Ç****<br>E*** S*** K**	N**** E****	SAKARYA ŞEHİT ÜSTEĞMEN İBRAHİM ABANOZ ANADOLU LİSESİ	FARKLI TÜRDE OKULLARDA ÇALIŞAN ÖĞRETMENLERİN PRESENTEİZM DÜZEYLERİ İLE İÇSEL MOTİVASYONLARI ARASINDAKİ İLİŞKİNİN İNCELENMESİ	PSİKOLOJİ	Değerler Eğitimi	İSTANBUL-ASYA	BÖLGE ÜÇÜNCÜLÜĞÜ
191	1689B012410823	E*** E** K******<br>E*** H**** Ü***********	V***** A******** K****	KOCAELİ ÖZEL SINAV KOLEJİ ŞAHİN FEN LİSESİ	2023 KAHRAMANMARAŞ VE HATAY DEPREMLERİ SONRASI ÖĞRENCİ GÖÇLERİ: SOSYOLOJİK BİR İNCELEME	SOSYOLOJİ	Doğal Afetler ve Afet Yönetimi	İSTANBUL-ASYA	BÖLGE BİRİNCİLİĞİ
192	1689B012418601	A****** E***<br>B**** A*****<br>D**** H*********	V***** A******** K****	KOCAELİ MUAMMER DERELİ FEN LİSESİ	SOSYAL MEDYADA YANKI ODALARI VE FİLTRE BALONLARININ ETKİSİ: LİSE ÖĞRENCİLERİNE YÖNELİK UYGULAMALI BİR ARAŞTIRMA	SOSYOLOJİ	Medya Okuryazarlığı	İSTANBUL-ASYA	BÖLGE BİRİNCİLİĞİ
193	1689B012430648	E*** T**** D***<br>U*** T****<br>G**** E*** K****	C*** Ö*****	İSTANBUL AHMET YÜKSEL ÖZEMRE BİLİM VE SANAT MERKEZİ	SİSTEMLER ARASINDA KÖPRÜ KURMAK: BİLSEM, ORTAOKUL 7. 8. SINIFLARDAKİ ÜSTÜN YETENEKLİ ÇOCUKLARIN SOSYAL KABUL SÜREÇLERİ	SOSYOLOJİ	Yaşamımızda İyilik, Nezaket ve Anlayış	İSTANBUL-ASYA	BÖLGE İKİNCİLİĞİ
194	1689B012403016	Y**** E** S*****	İ***** A***	İSTANBUL İSTANBUL FUAT SEZGİN BİLİM VE SANAT MERKEZİ	NANOTEKNOLOJİNİN TOPLUMSAL DEĞİŞİM İLİŞKİSİNİN SOSYAL İNŞA KURAMI BAĞLAMINDA İNCELENMESİ	SOSYOLOJİ	Bilim İletişimi	İSTANBUL-ASYA	BÖLGE ÜÇÜNCÜLÜĞÜ
195	1689B012429362	D**** K***<br>M****** Y**** A***<br>A**** A*** U****	İ**** F****** Ç****	İSTANBUL MALTEPE KADİR HAS BİLİM VE SANAT MERKEZİ	TARİHİ ESER RESTORASYONLARINDA FOTOGRAMETRİ YÖNTEMLERİ KULLANILARAK 3 BOYUTLU YAZICILAR İLE ASLINA UYGUN YAPI ELEMANLARININ OLUŞTURULMASI	TARİH	Görüntü ve Ses Tanıma Teknolojileri	İSTANBUL-ASYA	BÖLGE BİRİNCİLİĞİ
196	1689B012408228	M******* T**** A*****<br>A**** T**** Y****<br>B****** H**** Ö*****	T**** E****	SAKARYA ADAPAZARI ANADOLU İMAM HATİP LİSESİ	TAŞLAR DA KONUŞUR; OSMANLI DA MEZAR TAŞLARININ DİLİ ÖRNEKLEMİ	TARİH	Kültürel Miras	İSTANBUL-ASYA	BÖLGE İKİNCİLİĞİ
197	1689B012423969	N***** Z***** S***<br>A**** U*****<br>E****** S***	A** Y*****	İSTANBUL KARTAL ANADOLU İMAM HATİP LİSESİ<br>GİRESUN GİRESUN FEN LİSESİ<br>KOCAELİ MEHMET AKİF ERSOY KIZ ANADOLU İMAM HATİP LİSESİ	GİRESUN'DAN İSTANBUL'A YAVUZ SULTAN SELİM KÜLLİYESİ	TARİH	Artırılmış, Sanal ve Karma Gerçeklik	İSTANBUL-ASYA	BÖLGE ÜÇÜNCÜLÜĞÜ
198	1689B012438641	Ş***** Y*****<br>E**** U***** Y*****<br>T**** C** K********	M***** D*****	İSTANBUL İSTANBUL ATATÜRK FEN LİSESİ	EPİTECH: EPİLEPSİ NÖBET TAKİP TEKNOLOJİLERİ	TEKNOLOJİK TASARIM	Giyilebilir Teknolojiler	İSTANBUL-ASYA	BÖLGE BİRİNCİLİĞİ
199	1689B012439027	A*** E*** Ö******<br>I**** G****<br>E*** K***	V**** Ş****	İSTANBUL İSTANBUL ATATÜRK FEN LİSESİ	HAYAT (HASTALIKLARIN YAZILIMLA TESPİTİ)	TEKNOLOJİK TASARIM	Biyomedikal Cihaz Teknolojileri	İSTANBUL-ASYA	BÖLGE BİRİNCİLİĞİ
200	1689B012406446	A**** M***** M******<br>A**** K****	P**** D*****	İSTANBUL KARTAL ANADOLU İMAM HATİP LİSESİ	EAGLES: YENİ NESİL AÇIK KAYNAK MİLLİ VE SİVİL İNSANSIZ KONTROL SİSTEMİ	TEKNOLOJİK TASARIM	Milli Teknoloji Hamlesi	İSTANBUL-ASYA	BÖLGE İKİNCİLİĞİ
201	1689B012403484	H**** K*****	P**** Ö******	İSTANBUL ARAŞTIRMA GELİŞTİRME EĞİTİM VE UYGULAMA MERKEZİ LİSESİ	KİMSE YOK MU	TEKNOLOJİK TASARIM	Doğal Afetler ve Afet Yönetimi	İSTANBUL-ASYA	BÖLGE ÜÇÜNCÜLÜĞÜ
202	1689B012432159	A***** O***<br>Y****** K****	İ*** D****	İSTANBUL ÖZEL EYÜBOĞLU KOLEJİ	"BİR YUDUM DA EDEBİYAT": DİJİTAL KİTAP KAFE	TÜRK DİLİ VE EDEBİYATI	Dil ve Edebiyat	İSTANBUL-ASYA	BÖLGE BİRİNCİLİĞİ
203	1689B012436535	E** Y******<br>Y**** C**** Y*****	E**** K********	İSTANBUL İSTANBUL FUAT SEZGİN BİLİM VE SANAT MERKEZİ	CUMHURİYETİMİZİN 100. YILINDA MİLLİ MÜCADELE DÖNEMİNE ROMAN KAHRAMANLARIYLA YOLCULUK	TÜRK DİLİ VE EDEBİYATI	Dijital Dönüşüm	İSTANBUL-ASYA	BÖLGE İKİNCİLİĞİ
204	1689B012403254	K**** H*******	A*** K*****	İSTANBUL ÖZEL TUZLA UĞUR ANADOLU LİSESİ	KONUŞAN ŞAİRLER: TÜRK EDEBİYATININ BÜYÜK ŞAİRLERİYLE SANAL ETKİLEŞİM	TÜRK DİLİ VE EDEBİYATI	Yapay Zekâ	İSTANBUL-ASYA	BÖLGE ÜÇÜNCÜLÜĞÜ
205	1689B012422235	R**** M*** B******<br>A*** C****<br>R**** N** S*****	Y******* E*******	İSTANBUL ÖZEL ANABİLİM ANADOLU LİSESİ<br>İSTANBUL ÖZEL ANABİLİM FEN VE TEKNOLOJİ LİSESİ	AI HAVA GÖZCÜSÜ: KİŞİSEL HAVA VE SAĞLIK TAKİBİ İÇİN BENZERSİZ BİR YAPAY ZEKâ TEKNOLOJİSİ	YAZILIM	Giyilebilir Teknolojiler	İSTANBUL-ASYA	BÖLGE BİRİNCİLİĞİ
206	1689B012429529	M****** T***** A*******<br>İ****** U*** D*****<br>Ö*** Y**** Y****	S***** E***	İSTANBUL İSTANBUL ATATÜRK FEN LİSESİ<br>İSTANBUL İSTANBUL TİCARET ODASI MARMARA ANADOLU İMAM HATİP LİSESİ	AGDES: AFET GÜZERGAH DENETİM SİSTEMİ	YAZILIM	Doğal Afetler ve Afet Yönetimi	İSTANBUL-ASYA	BÖLGE İKİNCİLİĞİ
207	1689B012436526	M******* E*** A****<br>E*** Y*****		İSTANBUL ÖZEL NUN ANADOLU LİSESİ	EXOLIFE: MAKİNE ÖĞRENMESİ KULLANARAK ÖTEGEZEGENLERİN TESPİT EDİLMESİ VE YAŞANABİLİRLİK TAHMİNİ YAPILMASI	YAZILIM	Astronomi ve Astrofizik	İSTANBUL-ASYA	BÖLGE ÜÇÜNCÜLÜĞÜ
208	1689B012405831	E** D**** K******	N*** Y*****	İSTANBUL HİSAR EĞİTİM VAKFI ÖZEL HİSAR LİSESİ	ARABİCA CİNSİ KAHVE ÇEKİRDEKLERİNDEN ELDE EDİLEN EKSOZOMLARIN MELANOMA TÜRÜ CİLT KANSERİ HÜCRELERİNE ETKİLERİ	BİYOLOJİ	Genetik ve Biyoteknoloji	İSTANBUL-AVRUPA	BÖLGE BİRİNCİLİĞİ
209	1689B012428618	Y**** Y******<br>E**** T****<br>H*** N** O********	B**** Y*****	İSTANBUL KAĞITHANE KIZ ANADOLU İMAM HATİP LİSESİ	RETİLİFE (RETİNOPATİ HASTALIĞINA ERKEN TEŞHİS VE TAKİP YAPABİLME İMKANI SUNAN SAĞLIK KİTİ)	BİYOLOJİ	Biyomedikal Cihaz Teknolojileri	İSTANBUL-AVRUPA	BÖLGE BİRİNCİLİĞİ
210	1689B012423013	C**** K********<br>S**** K**** C**	S*** K****** K*****	İSTANBUL TED ÖZEL ATAKENT ANADOLU LİSESİ	SALVİA ROSMARİNUS RİZOSFERİNDEKİ MİKROORGANİZMA FAALİYETLERİ SONUCUNDA BİYOELEKTRİK ÜRETİLMESİ VE MİKORİZA UYGULAMALARININ ÜRETİM MİKTARINA ETKİSİ	BİYOLOJİ	Yenilenebilir Enerji	İSTANBUL-AVRUPA	BÖLGE İKİNCİLİĞİ
211	1689B012429417	M**** E*******<br>Y***** Ö****	S****** Ö******	İSTANBUL VEFA LİSESİ<br>İSTANBUL ÇEMBERLİTAŞ ANADOLU LİSESİ	MOLEKÜLER DOCKİNG YÖNTEMİYLE YENİ BİR POTANSİYEL CDK4/6 İNHİBİTÖRÜNÜN GELİŞTİRİLMESİ, SENTEZLENMESİ VE ANTİKANSER ETKİSİNİN ARAŞTIRILMASI	BİYOLOJİ	Genetik ve Biyoteknoloji	İSTANBUL-AVRUPA	BÖLGE ÜÇÜNCÜLÜĞÜ
212	1689B012422522	E*** K**** G***<br>Ş*** Y*****	C** O*** B***	İSTANBUL ÖZEL DARUŞŞAFAKA LİSESİ	YÜZEY ŞEKİLLERİNİN FARKLI ÖĞRENME STİLLERİNDE ÖĞRETİLMESİNDE ARTIRILMIŞ GERÇEKLİK VE 3B MODELLERİN KULLANILMASINA ÖRNEK: GEOCUBE	COĞRAFYA	STEAM (Fen, Teknoloji, Mühendislik, Sanat ve Matematik)	İSTANBUL-AVRUPA	BÖLGE BİRİNCİLİĞİ
213	1689B012422007	T***** A***<br>Ö*** Y**** Z*****	F***** B*****	İSTANBUL ÖZEL ESENKENT OKYANUS ANADOLU LİSESİ	ATMOSFERDEN TARLALARA: SÜRDÜRÜLEBİLİR SU ÜRETİMİ	COĞRAFYA	Su Okuryazarlığı	İSTANBUL-AVRUPA	BÖLGE İKİNCİLİĞİ
214	1689B012413575	B**** S*******<br>B*** S*****<br>A**** Ö*****	S***** D******	TEKİRDAĞ TEKİRDAĞ BELEDİYESİ MEHMET SEREZ SOSYAL BİLİMLER LİSESİ<br>TEKİRDAĞ EBRU NAYİM FEN LİSESİ	YEP-YEŞİL PANSİYON PROJESİ	COĞRAFYA	Dijital Dönüşüm	İSTANBUL-AVRUPA	BÖLGE ÜÇÜNCÜLÜĞÜ

SIRA NO	BAŞVURU NO	ÖĞRENCİ ADI SOYADI	DANIŞMAN ADI SOYADI	OKULU	PROJENİN ADI	ANA ALANI	TEMATİK ALANI	BÖLGESİ	BÖLGE AŞAMASI SONUCU
215	1689B012413727	İ*** B**********<br>B**** E*** Ö***	H**** M****	İSTANBUL ÖZEL AMERİKAN ROBERT LİSESİ<br>ADANA ÇUKUROVA BİLİM VE SANAT MERKEZİ	DİJİTAL DÜRÜSTLÜK	DEĞERLER EĞİTİMİ	Yapay Zekâ	İSTANBUL-AVRUPA	BÖLGE BİRİNCİLİĞİ
216	1689B012428452	F**** D**** A****	S***** T***	İSTANBUL TED ÖZEL ATAKENT ANADOLU LİSESİ	ZİHİNSEL YETERSİZLİĞİ BULUNAN ÖĞRENCİLERİN EĞİTİMİNDE DİJİTAL EĞİTİM TEKNOLOJİLERİNİN KULLANILMASI: SOSYAL YAŞAM BECERİLERİNİ GELİŞTİRMEYE YÖNELİK WEBSİTE	DEĞERLER EĞİTİMİ	Dijital Dönüşüm	İSTANBUL-AVRUPA	BÖLGE İKİNCİLİĞİ
217	1689B012431565	D*** S*******<br>D**** S******<br>B***** C*****	H***** K****	İSTANBUL ÖZEL BAŞAKŞEHİR BAHÇEŞEHİR KOLEJİ ANADOLU LİSESİ	ADAB-I MUAŞERET: "ÖZÜMÜZ, İZİMİZDE SAKLIDIR."	DEĞERLER EĞİTİMİ	Değerler Eğitimi	İSTANBUL-AVRUPA	BÖLGE ÜÇÜNCÜLÜĞÜ
218	1689B012436130	A**** C****	H**** K******	İSTANBUL GAZİOSMANPAŞA BİLİM VE SANAT MERKEZİ	C-VORTEKS DİKEY EKSENLİ RÜZGAR TÜRBİNİ	FİZİK	Yenilenebilir Enerji	İSTANBUL-AVRUPA	BÖLGE BİRİNCİLİĞİ
219	1689B012426450	K**** E** C*******	Ö*** K******	İSTANBUL DOĞAN CÜCELOĞLU FEN LİSESİ	GELECEĞİN QUANTUM HAYALETLERİ: GRAFEN QUANTUM IRAM MALZEMELER	FİZİK	Malzeme Bilimi ve Nanoteknoloji	İSTANBUL-AVRUPA	BÖLGE İKİNCİLİĞİ
220	1689B012411197	Z***** N**<br>N*** N** N******	M***** F**** A******	İSTANBUL ATATÜRK ANADOLU LİSESİ<br>İSTANBUL SUAT TERİMER ANADOLU LİSESİ	DERİN DENİZ ŞARTLARININ HİDROSTATİK BASINCA ETKİSİNİN GERÇEKÇİ DEĞERLENDİRMESİ	FİZİK	STEAM (Fen, Teknoloji, Mühendislik, Sanat ve Matematik)	İSTANBUL-AVRUPA	BÖLGE ÜÇÜNCÜLÜĞÜ
221	1689B012419008	B****** E** B********	V****** M***** F*****	İSTANBUL GALATASARAY ÜNİVERSİTESİ GALATASARAY LİSESİ	YENİLİKÇİ NANOMALZEMELERİN MİLLİ HAVA ARAÇLARIMIZIN GÖRÜNMEZLİĞİNE YÖNELİK KAPLAMA MALZEMESİ OLARAK YÜKSEK VERİMDE KULLANIMININ ARAŞTIRILMASI	KİMYA	Malzeme Bilimi ve Nanoteknoloji	İSTANBUL-AVRUPA	BÖLGE BİRİNCİLİĞİ
222	1689B012428534	A*** N*** U***<br>M****** M**** S******	M**** K***	İSTANBUL SAMİHA AYVERDİ ANADOLU LİSESİ<br>İSTANBUL HÜSEYİN BÜRGE ANADOLU LİSESİ	IŞIMA YAPABİLEN, ŞEKİL HAFIZALI POLİMERİK STENT GELİŞTİRİLMESİ VE ANTİMİKROBİYAL ETKİNLİĞİNİN İNCELENMESİ	KİMYA	Malzeme Bilimi ve Nanoteknoloji	İSTANBUL-AVRUPA	BÖLGE İKİNCİLİĞİ
223	1689B012433122	A*** E***<br>E*** B***** A***	M**** K***	İSTANBUL VEFA LİSESİ<br>İSTANBUL ADİLE MERMERCİ ANADOLU LİSESİ	DERİ ALTI (SUBKUTAN) PORTLARA ENTEGRE EDİLEBİLECEK KENDİNİ ONARABİLEN POLİMER/NANOÇİÇEK KOMPOZİTLERİNİN GELİŞTİRİLMESİ	KİMYA	Malzeme Bilimi ve Nanoteknoloji	İSTANBUL-AVRUPA	BÖLGE ÜÇÜNCÜLÜĞÜ
224	1689B012428687	H***** G*****	B**** Ö*** E***	İSTANBUL ÖZEL DARUŞŞAFAKA LİSESİ	KARELER İLE BİR DİKDÖRTGEN OLUŞTURARAK ALANLAR YARDIMIYLA BAZI SAYISAL EŞİTLİKLERİN ELDE EDİLMESİ	MATEMATİK	Özgün Algoritma Tasarımı	İSTANBUL-AVRUPA	BÖLGE BİRİNCİLİĞİ
225	1689B012430131	F**** E***	Y****** B******	İSTANBUL BEŞİKTAŞ BİLİM VE SANAT MERKEZİ	PAKETLEMEDE KULLANILABİLECEK BİRİM KENARLI DÜZGÜN N-GENİ KAPATACAK MİNİMUM ALANLI DÜZGÜN (N+1)-GENİN ALANININ GENELLENMESİ	MATEMATİK	STEAM (Fen, Teknoloji, Mühendislik, Sanat ve Matematik)	İSTANBUL-AVRUPA	BÖLGE BİRİNCİLİĞİ
226	1689B012437007	E**** A** Y*****	Y****** B******	İSTANBUL BEŞİKTAŞ BİLİM VE SANAT MERKEZİ	ZECKENDORF TÜMLEME İŞLEMİ İLE VİGENERE ŞİFRELEME	MATEMATİK	Siber Güvenlik	İSTANBUL-AVRUPA	BÖLGE İKİNCİLİĞİ
227	1689B012427951	Ö**** E**<br>A*** E**<br>Ç***** E**	B**** Ö*** E***	İSTANBUL ÖZEL DARUŞŞAFAKA LİSESİ	KENAR UZUNLUKLARI FİBONACCİ SAYISI OLAN EŞKENAR ÜÇGENLERİN TEPE NOKTALARI İLE OLUŞTURULMUŞ ÇOKGENLERDE GİZEMLİ ALAN İLİŞKİSİ	MATEMATİK	Özgün Algoritma Tasarımı	İSTANBUL-AVRUPA	BÖLGE ÜÇÜNCÜLÜĞÜ
228	1689B012414828	İ*** A***<br>G***** Ö******	A**** A***	KIRKLARELİ LÜLEBURGAZ LİSESİ	TİK-TOK VİDEOLARININ LİSE ÖĞRENCİLERİNİN AHLAKİ DEĞERLERİ ÜZERİNE ETKİSİ	PSİKOLOJİ	Değerler Eğitimi	İSTANBUL-AVRUPA	BÖLGE BİRİNCİLİĞİ
229	1689B012422356	N**** G****	V***** T*******	İSTANBUL ESENLER PROF. DR. SADETTİN ÖKTEN BİLİM VE SANAT MERKEZİ	BİLİM VE SANAT MERKEZİ ÖĞRENCİLERİNİN SOSYAL MEDYA TUTUMLARI	PSİKOLOJİ	Bağımlılık ve Bağımlılıkla Mücadele	İSTANBUL-AVRUPA	BÖLGE İKİNCİLİĞİ
230	1689B012423136	A****** E**** D******	Ö*** T**** K****	İSTANBUL İSTANBUL TİCARET ODASI BİLİM VE SANAT MERKEZİ	ORTAOKUL BİLSEM ÖĞRENCİLERİNİN AKADEMİK ERTELEME DAVRANIŞLARI İLE MÜKEMMELİYETÇİLİK DÜZEYLERİ ARASINDAKİ İLİŞKİNİN İNCELENMESİ	PSİKOLOJİ	Bilinçli Farkındalık ve Kariyer Bilinci	İSTANBUL-AVRUPA	BÖLGE ÜÇÜNCÜLÜĞÜ
231	1689B012406431	İ*** A**********<br>S**** D******	İ*** B***	İSTANBUL EMLAK KONUT MİMAR SİNAN ANADOLU LİSESİ	İNSANI DÜNYAYA, DÜNYAYI İNSANA AÇIYOR; YAŞLI KİŞİLERİN DİJİTAL TEKNOLOJİLERLE KURDUĞU İLİŞKİ ÜZERİNE BİR İNCELEME	SOSYOLOJİ	Değerler Eğitimi	İSTANBUL-AVRUPA	BÖLGE BİRİNCİLİĞİ
232	1689B012436621	K***** Y*****<br>İ**** S**** Ö*****<br>E****** Ö*****	M***** M***** V****	İSTANBUL RECEP TAYYİP ERDOĞAN KIZ ANADOLU İMAM HATİP LİSESİ	LİSE ÖĞRENCİLERİNİN ÇEVRE BİLİNCİ, TEKSTİL ATIKLARIYLA İLGİLİ FARKINDALIĞI: DOĞA İÇİN FARK YARATAN GERİ DÖNÜŞÜM PROJESİ	SOSYOLOJİ	Atık Yönetimi ve Geri Dönüşüm	İSTANBUL-AVRUPA	BÖLGE İKİNCİLİĞİ
233	1689B012409154	B**** D**** Ç****<br>D**** A***<br>E*** A****	S***** D******	TEKİRDAĞ TEKİRDAĞ BELEDİYESİ MEHMET SEREZ SOSYAL BİLİMLER LİSESİ	KİRACI VE EV SAHİBİ SORUNUNUN SOSYOLOJİK VE HUKUKSAL AÇIDAN İNCELENMESİ	SOSYOLOJİ	İnsan Hakları ve Demokrasi	İSTANBUL-AVRUPA	BÖLGE ÜÇÜNCÜLÜĞÜ
234	1689B012420929	C**** Ç*********<br>M***** B******	C** O*** B***	İSTANBUL ÖZEL DARUŞŞAFAKA LİSESİ	PİRİ REİS'İN AKDENİZ HARİTALARININ COĞRAFİ BİLGİ SİSTEMLERİ İLE WEB ORTAMINDA YAYINLANMASI: PİRİ 1513	TARİH	Dijital Dönüşüm	İSTANBUL-AVRUPA	BÖLGE BİRİNCİLİĞİ
235	1689B012422798	K**** S*** K***<br>H**** İ****** B****	A*** H***** K******	İSTANBUL ÖZEL İHLAS FEN LİSESİ<br>İSTANBUL ÇAPA FEN LİSESİ	ZAMANIN MÜHRÜ: SÖZLÜ TARİH IŞIĞINDA 1960-1974 OLAYLARI VE KIBRISLI TÜRKLERİN ANLATILARI	TARİH	Kültürel Miras	İSTANBUL-AVRUPA	BÖLGE BİRİNCİLİĞİ
236	1689B012422369	Y***** A*****<br>N**** A****<br>B**** B**** B*****	U*** E***	EDİRNE ÖZEL EDİRNE TED KOLEJİ ANADOLU LİSESİ	EDİRNE'NİN UNUTULAN TARİHİ: BALON HANGARI	TARİH	Kültürel Miras	İSTANBUL-AVRUPA	BÖLGE İKİNCİLİĞİ
237	1689B012409951	M******* M**** D********<br>E**** İ****** Ş**	A*** H***** K******	İSTANBUL ÖZEL İHLAS FEN LİSESİ	OLİMPİYAT OYUNLARINDA PARLAYAN TÜRK YILDIZLARI: ZAFERİN İZİNDE TARİHİ BİR YOLCULUK	TARİH	Kültürel Miras	İSTANBUL-AVRUPA	BÖLGE ÜÇÜNCÜLÜĞÜ
238	1689B012428265	A**** K*****<br>T*** K*******<br>Y**** E*** K******	G***** E***	İSTANBUL ÖZEL DARUŞŞAFAKA LİSESİ	AÇIK DENİZDE ARAMA KURTARMA ÇALIŞMALARI İÇİN AKILLI GÖRÜNTÜ İŞLEME TABANLI BİR SABİT KANAT İHA GELİŞTİRİLMESİ	TEKNOLOJİK TASARIM	Havacılık ve Uzay Bilimleri	İSTANBUL-AVRUPA	BÖLGE BİRİNCİLİĞİ
239	1689B012436431	B**** S*****	K****** C***** Ş********	EDİRNE EDİRNE SÜLEYMAN DEMİREL FEN LİSESİ	OTOYOL TRANSINA YÖNELİK SÜRÜCÜ ANALİZ SİSTEMİ	TEKNOLOJİK TASARIM	Akıllı Ulaşım Sistemleri	İSTANBUL-AVRUPA	BÖLGE BİRİNCİLİĞİ
240	1689B012426627	M******* S******* K*****<br>Z***** S**** E****<br>Z***** A**	H**** O*** S*****	İSTANBUL ÖZEL FLORYA ANADOLU LİSESİ<br>İSTANBUL ÖZEL FLORYA FEN LİSESİ	NESNELERİN İLETİŞİMİ AC AKIM KONTROLLÜ AKILLI PRİZ AKAP	TEKNOLOJİK TASARIM	Robotik ve Kodlama	İSTANBUL-AVRUPA	BÖLGE İKİNCİLİĞİ
241	1689B012401926	Y**** G****<br>A*** S*********	E**** A*****	TEKİRDAĞ ÇERKEZKÖY TÜRK TEKSTİL VAKFI MESLEKİ VE TEKNİK ANADOLU LİSESİ	EKOLOJİK BEBEK GİYSİLERİ İÇİN DOĞAL BOYAMA	TEKNOLOJİK TASARIM	Çevre ve Çevreyi Koruma	İSTANBUL-AVRUPA	BÖLGE ÜÇÜNCÜLÜĞÜ
242	1689B012424160	I*** Ş*****<br>Z****** H**** B****	A****** C****** V****	İSTANBUL ÖZEL ESENKENT OKYANUS ANADOLU LİSESİ	MİNYATÜR EDEBİYAT MÜZESİ İLE ROMANLARA YOLCULUK YAPALIM	TÜRK DİLİ VE EDEBİYATI	Görsel ve İşitsel Sanatlar	İSTANBUL-AVRUPA	BÖLGE BİRİNCİLİĞİ
243	1689B012432523	M**** G****	A*** R**** Ö***	İSTANBUL İSTEK ÖZEL KAŞGARLI MAHMUT ANADOLU LİSESİ	GURBETLE SILANIN BİRBİRİNE KARIŞTIĞI GÖÇ HİKâYELERİ: GÖÇ ÖYKÜLERİNİ GELECEĞE TAŞIMAK	TÜRK DİLİ VE EDEBİYATI	Göç ve Uyum	İSTANBUL-AVRUPA	BÖLGE BİRİNCİLİĞİ
244	1689B012412332	G**** Ç****<br>M******* A*******<br>N**** T******	C**** Ç****** B******	İSTANBUL BEŞİKTAŞ SAKIP SABANCI ANADOLU LİSESİ	DEDE KORKUT KÜTÜPHANESİ: LİSE VE ORTAOKUL ÖĞRENCİLERİ İÇİN DEDE KORKUT HİKâYELERİ BİLGİ KAYNAĞI	TÜRK DİLİ VE EDEBİYATI	Kültürel Miras	İSTANBUL-AVRUPA	BÖLGE İKİNCİLİĞİ
245	1689B012410028	G***** Ç****<br>N*** S*****<br>M**** Ç****	R**** Ç*****	EDİRNE YUSUF ÇAPRAZ ANADOLU LİSESİ	TÜRKÇE-İNGİLİZCE SESLİ VE RESİMLİ NASRETTİN HOCA FIKRALARI DİJİTAL KİTABI	TÜRK DİLİ VE EDEBİYATI	Dijital Dönüşüm	İSTANBUL-AVRUPA	BÖLGE ÜÇÜNCÜLÜĞÜ

SIRA NO	BAŞVURU NO	ÖĞRENCİ ADI SOYADI	DANIŞMAN ADI SOYADI	OKULU	PROJENİN ADI	ANA ALANI	TEMATİK ALANI	BÖLGESİ	BÖLGE AŞAMASI SONUCU
246	1689B012408631	A** K*** N****<br>A** D******	F**** K***** C****	İSTANBUL ÖZEL AMERİKAN ROBERT LİSESİ	EVRİŞİMSEL SİNİR AĞI (ESA) TABANLI DİKKAT MODELİ KULLANILARAK MEME KANSERİ ULTRASONLARINDA GÖRÜNTÜ BÖLÜTLEME VE TEŞHİS	YAZILIM	Yapay Zekâ	İSTANBUL-AVRUPA	BÖLGE BİRİNCİLİĞİ
247	1689B012432266	Ö*** K***** T****<br>İ*** K*********	Ö**** S***	İSTANBUL ALMAN LİSELİLER KÜLTÜR VE EĞİTİM VAKFI ÖZEL FEN LİSESİ	SERVİKAL SAĞLIK İÇİN AKILLI DURUŞ ASİSTANI	YAZILIM	Görüntü ve Ses Tanıma Teknolojileri	İSTANBUL-AVRUPA	BÖLGE İKİNCİLİĞİ
248	1689B012423022	Ş***** Ö*********<br>Ş***** B****	T*** K****	İSTANBUL EŞREF BİTLİS ANADOLU LİSESİ	MASK R-CNN ALGORİTMASI İLE KAHRAMANMARAŞ DEPREMİNİN HASAR TESPİTİ ÇALIŞMASI: ANTAKYA ÖRNEĞİ	YAZILIM	Yapay Zekâ	İSTANBUL-AVRUPA	BÖLGE ÜÇÜNCÜLÜĞÜ
249	1689B012428446	U**** C*****<br>D**** B***<br>Z***** İ*** T****	Y****** B***** Y****	İZMİR ÖZEL EGE LİSESİ	GLİOBLASTOMA MULTİFORME BEYİN TÜMÖRÜNE NEDEN OLAN MUTASYONLU HRAS V12 GENİNİN ZEBRA BALIĞINDA (DANİO RERİO) MODELLENMESİ	BİYOLOJİ	Genetik ve Biyoteknoloji	İZMİR	BÖLGE BİRİNCİLİĞİ
250	1689B012431591	Ç**** Ş****<br>A****** S*******	H**** E*****	İZMİR İZMİR FEN LİSESİ	VİBRİO HARVEYİ LUXN PROTEİNİNİ HEDEFLEYEN İNHİBİTÖRLERİN MOLEKÜLER DOCKİNG İLE SEÇİMİ VE BİYOFİLM İNHİBİSYON POTANSİYELİNİN ARAŞTIRILMASI	BİYOLOJİ	Biyoçeşitlilik	İZMİR	BÖLGE BİRİNCİLİĞİ
251	1689B012432076	S**** K*******<br>Z***** D*** D****	C** A***	İZMİR ÖZEL TAKEV ANADOLU LİSESİ	M6A RNA MODİFİKASYONUNUN TRAIL UYGULANMIŞ MDA-MB-231 MEME KANSERİ HÜCRELERİNDE APOPTOZA ETKİSİ	BİYOLOJİ	Genetik ve Biyoteknoloji	İZMİR	BÖLGE BİRİNCİLİĞİ
252	1689B012420090	I**** Y*****<br>E*** E****<br>S*** K*******	O*** A******	İZMİR ÖZEL EGE LİSESİ	DİYABETE DÜŞMAN ÇEVREYLE DOST: NAR KABUĞU ATIKLARINDAN FULVİK ASİT DESTEKLİ ANTİDİYABETİK ORAL FİLM GELİŞTİRİLMESİ	BİYOLOJİ	Genetik ve Biyoteknoloji	İZMİR	BÖLGE İKİNCİLİĞİ
253	1689B012433107	N** S*******	E*** T****	İZMİR ÖZEL İZMİR BAHÇEŞEHİR KOLEJİ ELLİNCİ YIL FEN VE TEKNOLOJİ LİSESİ	KOLOREKTAL KANSER TEDAVİSİNDE PHB DEPOLİMERAZ ENZİMİ VE PARÇALANMA ÜRÜNLERİ ETKİLİ OLABİLİR Mİ?	BİYOLOJİ	Genetik ve Biyoteknoloji	İZMİR	BÖLGE ÜÇÜNCÜLÜĞÜ
254	1689B012426026	S****** K********<br>Ş******** A******<br>M***** F**** B*********	N********** S****	MANİSA ALAŞEHİR FEN LİSESİ	UZAKTAN ALGILAMA İLE OTONOM SULAMA SİSTEMİ	COĞRAFYA	Tarım ve Hayvancılık Teknolojileri	İZMİR	BÖLGE BİRİNCİLİĞİ
255	1689B012435375	N****** G*****<br>D*** A****<br>E*** Ö*****	G***** K****	İZMİR HABAŞ MEHMET RÜŞTÜ BAŞARAN BİLİM VE SANAT MERKEZİ<br>İZMİR TÜRKİYE ODALAR VE BORSALAR BİRLİĞİ ALOSBİ MESLEKİ VE TEKNİK ANADOLU LİSESİ	ALİAĞA'DA MOR TABELA İLE COĞRAFYAMI TANIYORUM	COĞRAFYA	Doğal Miras ve Doğal Kaynaklar	İZMİR	BÖLGE İKİNCİLİĞİ
256	1689B012427891	Y**** S**** A*******<br>R**** E*** A****<br>E*** Z*****	M*** Ç****	MANİSA SALİHLİ BİLİM VE SANAT MERKEZİ	BOZDAĞLARIN KUZEY YAMAÇLARINDA OYUNTU EROZYONU SONUCU OLUŞAN PERİBACASI BENZERİ OLUŞUMLARIN FOTOGRAMETRİK YÖNTEMLE TESPİTİ VE ANALİZİ	COĞRAFYA	Doğal Miras ve Doğal Kaynaklar	İZMİR	BÖLGE ÜÇÜNCÜLÜĞÜ
257	1689B012428302	E** D****<br>B**** E** B*****<br>Ö*** A** E****	H**** Ö****	AYDIN AYDIN LİSESİ	KONUŞAN ŞEHİTLİKLER	DEĞERLER EĞİTİMİ	Artırılmış, Sanal ve Karma Gerçeklik	İZMİR	BÖLGE BİRİNCİLİĞİ
258	1689B012404807	C***** B*******<br>M****** A****** Y*******<br>E*** N**** Ş****	B**** Y***********	DENİZLİ NEZİHE-DERYA BALTALI BİLİM VE SANAT MERKEZİ	CUMHURİYET'İMİZİN YÜZÜNCÜ YILINDA "ANILARLA CUMHURİYET"	DEĞERLER EĞİTİMİ	Değerler Eğitimi	İZMİR	BÖLGE İKİNCİLİĞİ
259	1689B012426370	M**** D*******<br>S****** Ş****	İ** Ö*****	AYDIN AYDIN SOSYAL BİLİMLER LİSESİ	ANADOLU MASALLARI İLE DEĞERLERİMİZİ ÖĞRENİYORUZ	DEĞERLER EĞİTİMİ	Değerler Eğitimi	İZMİR	BÖLGE ÜÇÜNCÜLÜĞÜ
260	1689B012418379	C**** G*****<br>A*** B****<br>A***** D****	O*** K******	İZMİR ÖZEL BİLFEN FEN LİSESİ<br>İZMİR ÖZEL BİLFEN ANADOLU LİSESİ	IŞIĞIN YOLUNDAN AYRILMA, KLOROFİLİN İZİNİ BIRAKMA!	FİZİK	STEAM (Fen, Teknoloji, Mühendislik, Sanat ve Matematik)	İZMİR	BÖLGE BİRİNCİLİĞİ
261	1689B012421671	O*** Ş****<br>C**** D**** S**	G**** K****	İZMİR İZMİR BÜYÜK ÇİĞLİ ÖZEL TÜRK KOLEJİ ANADOLU LİSESİ<br>İZMİR İZMİR BÜYÜKÇİĞLİ ÖZEL TÜRK KOLEJİ FEN LİSESİ	PROTON-PROTON ÇARPIŞMALARINDA JET SAYISININ MAKİNE ÖĞRENİMİ VE DERİN ÖĞRENME MODELLERİ KULLANILARAK TAHMİN EDİLMESİ	FİZİK	Yapay Zekâ	İZMİR	BÖLGE İKİNCİLİĞİ
262	1689B012419724	S*** V********<br>Y**** E** E****	D**** İ****	İZMİR ÖZEL ÇAKABEY LİSESİ	AYARLANABİLİR ÖZGÜN MANYETİK YÜZDÜRME SİSTEMİ TASARIMI VE BİYOMEDİKAL UYGULAMALARI	FİZİK	Biyomedikal Cihaz Teknolojileri	İZMİR	BÖLGE ÜÇÜNCÜLÜĞÜ
263	1689B012411419	C** H*****	Z***** B**** H*********	İZMİR ATATÜRK LİSESİ	SERBEST VE KİTOSAN-HALLOYSİTE BONCUKLARINA İMMOBİLİZE EDİLMİŞ TERMOALKALİFİLİK REKOMBİNANT ESTERAZ ENZİMİYLE POLİETİLEN TEREFTALAT (PET) FİBERLERİNİN ARITIMI	KİMYA	Çevre ve Çevreyi Koruma	İZMİR	BÖLGE BİRİNCİLİĞİ
264	1689B012415677	A**** G*****<br>A**** A*****	S**** Ş*****	İZMİR ÖZEL BİLFEN FEN LİSESİ	FOTOKATALİTİK REAKTÖRDE HİDROJEN ÜRETİMİ İÇİN ŞEKER PANCARI ATIK SUYUNUN DEĞERLENDİRİLMESİ	KİMYA	Hidrojen Enerjisi	İZMİR	BÖLGE İKİNCİLİĞİ
265	1689B012419037	Ö*** S* A***	F**** Ö*******	İZMİR ÖZEL BORNOVA MEKTEBİM ANADOLU LİSESİ	ATIK CAM VE KIRMIZI ÇAMURDAN BİYOTABANLI ÇEVRECİ GEOPOLİMER BETON ÜRETİMİ	KİMYA	Atık Yönetimi ve Geri Dönüşüm	İZMİR	BÖLGE ÜÇÜNCÜLÜĞÜ
266	1689B012427876	H**** A*** İ***	S***** S****	İZMİR ÖZEL TAKEV FEN LİSESİ	DOĞRUSAL DİYOFANT DENKLEM VE EŞİTSİZLİKLERİN ÇÖZÜM SAYILARI	MATEMATİK	STEAM (Fen, Teknoloji, Mühendislik, Sanat ve Matematik)	İZMİR	BÖLGE BİRİNCİLİĞİ
267	1689B012431502	E*** O*** K***	B**** K******	İZMİR BUCA İNCİ-ÖZER TIRNAKLI FEN LİSESİ	DİNAMİK KOMBİNASYONEL SIRALI OYUNLARIN KUANTUM HESAPLAMA KULLANILARAK STATİK OYUNA İNDİRGENMESİ VE EN İYİ STRATEJİNİN BELİRLENMESİ	MATEMATİK	Özgün Algoritma Tasarımı	İZMİR	BÖLGE BİRİNCİLİĞİ
268	1689B012410196	B***** Ö****<br>E*** Ö***<br>B**** Ş*** Y*****	S***** A***	İZMİR İZMİR FEN LİSESİ	AYRIK FOURİER DÖNÜŞÜMÜ ALGORİTMASI İLE VERİ GÜVENLİĞİ - FOURCE	MATEMATİK	Özgün Algoritma Tasarımı	İZMİR	BÖLGE İKİNCİLİĞİ
269	1689B012408281	H**** E** G***<br>E*** Y*****<br>E*** A***	İ***** S****	AYDIN NAZİLLİ ANADOLU LİSESİ	ARA-DEĞER TEOREMİ YARDIMIYLA DÜZLEM STABİLİZASYONU PROBLEMİ İÇİN BİR ÇÖZÜM	MATEMATİK	STEAM (Fen, Teknoloji, Mühendislik, Sanat ve Matematik)	İZMİR	BÖLGE ÜÇÜNCÜLÜĞÜ
270	1689B012408266	N*** G******<br>Ş**** B******<br>E**** N*** Y*****	C**** Y***	UŞAK TURHAN AKÇAY BİLİM VE SANAT MERKEZİ	LİSE ÖĞRENCİLERİNDE İKLİM KAYGISI VE GELECEĞE UMUT ÜZERİNE DENEYSEL BİR ÇALIŞMA	PSİKOLOJİ	Küresel Isınma ve İklim Değişikliği	İZMİR	BÖLGE BİRİNCİLİĞİ
271	1689B012426639	A***** S*****<br>Y**** D**** K***	M***** Y***** D****	İZMİR GAZİEMİR NEVVAR SALİH İŞGÖREN ANADOLU LİSESİ	LİSE ÖĞRENCİLERİNİN BİLİŞSEL ESNEKLİK DÜZEYLERİ İLE KARİYER KARARSIZLIĞI VE KARİYER KAYGISI DÜZEYLERİ ARASINDAKİ İLİŞKİLER	PSİKOLOJİ	Bilinçli Farkındalık ve Kariyer Bilinci	İZMİR	BÖLGE İKİNCİLİĞİ
272	1689B012426208	H**** H****** K****<br>D******* Y*******<br>B**** Y*****	Ö*** D*********	AYDIN AYDIN SOSYAL BİLİMLER LİSESİ	LİSE ÖĞRENCİLERİNDE TEKNOLOJİ BAĞIMLILIĞI VE PSİKOLOJİK YARDIM ALMA TUTUMU İLİŞKİSİNDE YALNIZLIĞIN ARACI ROLÜNÜN İNCELENMESİ	PSİKOLOJİ	Bağımlılık ve Bağımlılıkla Mücadele	İZMİR	BÖLGE ÜÇÜNCÜLÜĞÜ
273	1689B012401058	G***** A****<br>N***** S********<br>C***** M********	S***** C****	AYDIN KUŞADASI HASAN-FATMA ÖNAL ANADOLU LİSESİ	100 YILLIK ANILAR (LEFTERE'DEN, GÜZELÇAMLI'YA)	SOSYOLOJİ	Kültürel Miras	İZMİR	BÖLGE BİRİNCİLİĞİ
274	1689B012418407	Ö*** V*****	C***** C*** T****	MANİSA SOMA BİLİM VE SANAT MERKEZİ	ÇOCUKLARDA FİNANSAL OKURYAZARLIK BİLİNCİNİN ARTTIRILMASI PROJESİ- AİLE TASARRUF OYUNU: TAS-O	SOSYOLOJİ	Finansal Okuryazarlık	İZMİR	BÖLGE İKİNCİLİĞİ
275	1689B012434738	E*** E** T****	B**** Y***********	DENİZLİ NEZİHE-DERYA BALTALI BİLİM VE SANAT MERKEZİ	ÖZEL YETENEKLİ VE ÜSTÜN ZEKALI ÖĞRENCİLERDE BEYİN EROZYONU İLE ÇÖLLEŞME	SOSYOLOJİ	Göç ve Uyum	İZMİR	BÖLGE ÜÇÜNCÜLÜĞÜ
276	1689B012412891	E**** K*****<br>A**** Y*****<br>Z**** N** S*****	F**** E***	DENİZLİ MUSTAFA KAYNAK ANADOLU LİSESİ	KAYBOLMAYA YÜZ TUTAN DEĞER: KIZILHİSAR BARDAĞI	TARİH	Kültürel Miras	İZMİR	BÖLGE BİRİNCİLİĞİ

SIRA NO	BAŞVURU NO	ÖĞRENCİ ADI SOYADI	DANIŞMAN ADI SOYADI	OKULU	PROJENİN ADI	ANA ALANI	TEMATİK ALANI	BÖLGESİ	BÖLGE AŞAMASI SONUCU
277	1689B012418091	N*** S***<br>O*** Z****<br>C**** K******	S***** C****	AYDIN KUŞADASI HASAN-FATMA ÖNAL ANADOLU LİSESİ	TARİHİ KAÇIŞ ODASI (EĞİTİMDE KAÇIŞ ODALARININ KULLANIMINA İLİŞKİN BİR İNCELEME)	TARİH	Oyun ve Oyunlaştırma	İZMİR	BÖLGE İKİNCİLİĞİ
278	1689B012432425	M****** S**** N****<br>M***** E*** A******<br>E***** Ş*******	N**** C******	İZMİR ATATÜRK LİSESİ	İZMİR TORBALI METROPOLİS ANTİK KENTİ SANAL RESTORASYON VE MODELLEME İLE FARKINDALIK YARATMAK	TARİH	Kültürel Miras	İZMİR	BÖLGE ÜÇÜNCÜLÜĞÜ
279	1689B012419785	D**** K******<br>T**** Ö***********	S**** G*******	İZMİR ÖZEL ÇAKABEY LİSESİ	PARKİNSON HASTALARINDA DONMA FENOMENİ TESPİT VE UYARI SİSTEMİ	TEKNOLOJİK TASARIM	Giyilebilir Teknolojiler	İZMİR	BÖLGE BİRİNCİLİĞİ
280	1689B012430934	E** A*********<br>S*** R***** Ç*******<br>H***** N** D****	E**** A*******	AYDIN AYDIN FEN LİSESİ<br>AYDIN ÖZEL GELECEK EGEFEN ANADOLU LİSESİ	DEHB VE SP TANILI ÖĞRENCİLERİN AKADEMİK HAYATLARINA YARDIMCI, SES, GÖRÜNTÜ TANIMA TEKNOLOJİLERİ KULLANILARAK CİHAZ GELİŞTİRİLMESİ	TEKNOLOJİK TASARIM	Görüntü ve Ses Tanıma Teknolojileri	İZMİR	BÖLGE BİRİNCİLİĞİ
281	1689B012430972	E*** M**** T******<br>K*** F****	M*** D****	İZMİR ÖZEL TAKEV FEN LİSESİ	YAPAY ZEKA ARACILIĞIYLA MİKROPLASTİK TESPİTİ YAPAN HOLOGRAFİK MİKROSKOP	TEKNOLOJİK TASARIM	STEAM (Fen, Teknoloji, Mühendislik, Sanat ve Matematik)	İZMİR	BÖLGE İKİNCİLİĞİ
282	1689B012430142	C**** D**** Y*****<br>K**** B******<br>B**** B*****	T**** C*****	İZMİR ÖZEL GELİŞİM KOLEJİ<br>İZMİR ŞEHİT FATİH SATIR BİLİM VE SANAT MERKEZİ	HOLOGRAFİK 3 BOYUTLU NESNE GÖRÜNTÜLEME VE İZLEME SİSTEMİ	TEKNOLOJİK TASARIM	Artırılmış, Sanal ve Karma Gerçeklik	İZMİR	BÖLGE ÜÇÜNCÜLÜĞÜ
283	1689B012433268	Y***** G*****<br>E*** S*** K****<br>I*** A***	S**** Ç******	UŞAK TURHAN AKÇAY BİLİM VE SANAT MERKEZİ	ŞİİRLERİN YAPAY ZEKâ ARAÇLARIYLA GÖRSELLEŞTİRİLEREK FENEMONOLOJİK DESENLEMEDE BELİRLENEN KAVRAMLAR AÇISINDAN İNCELENMESİ	TÜRK DİLİ VE EDEBİYATI	Yapay Zekâ	İZMİR	BÖLGE BİRİNCİLİĞİ
284	1689B012433572	B***** K****<br>İ*** K***********	M***** Y**** U****	İZMİR TED ALİAĞA KOLEJİ VAKFI ÖZEL LİSESİ	YAPAY ZEKA İLE HAZIRLANAN MASALLARLA AKRAN ZORBALIĞINDA FARKINDALIK YARATILMASI	TÜRK DİLİ VE EDEBİYATI	Değerler Eğitimi	İZMİR	BÖLGE İKİNCİLİĞİ
285	1689B012402329	E**** A*******<br>E**** A*****	T**** K*******	İZMİR GAZİEMİR NEVVAR SALİH İŞGÖREN ANADOLU LİSESİ	Z-KUŞAĞI İLETİŞİMİNDE YENİ AKIM: LÜGAT PARALAMA MODASI	TÜRK DİLİ VE EDEBİYATI	Dil ve Edebiyat	İZMİR	BÖLGE ÜÇÜNCÜLÜĞÜ
286	1689B012431806	H***** E** C******<br>Y**** S**** K******<br>Ö*** A*****	G******* C*****	İZMİR İZMİR FEN LİSESİ	DEEP REİNFORCEMENT LEARNİNG İLE ORTAM KOŞULLARI VE VERİLEN GÖREVE BAĞLI MULTİDİSİPLİNER DRONE TASARIM OPTİMİZASYONU	YAZILIM	Yapay Zekâ	İZMİR	BÖLGE BİRİNCİLİĞİ
287	1689B012418980	A*** O***<br>R*** Ö****	H*** M**** S****	İZMİR ÖZEL ÇAKABEY LİSESİ	ULT AI: DERİN ÖĞRENME TABANLI ULTRASONOGRAFİ İLE MEME KANSERİ TEŞHİSİ	YAZILIM	Yapay Zekâ	İZMİR	BÖLGE İKİNCİLİĞİ
288	1689B012421282	K**** Y***<br>M******* H****** K***<br>M**** K*******	E**** A*******	AYDIN AYDIN FEN LİSESİ	FARKLI KANSER TÜRLERİNE YÖNELİK OLARAK RİSKLİ KİŞİLERİN TESPİTİNİN SAĞLANMASI VE KARAR DESTEK SİSTEMİ OLUŞTURULMASI: PATAIOJİ	YAZILIM	Dijital Dönüşüm	İZMİR	BÖLGE ÜÇÜNCÜLÜĞÜ
289	1689B012416609	C**** H********<br>E** P**** G****	M**** S******	NİĞDE NİĞDE YUNUS EMRE ANADOLU LİSESİ<br>NİĞDE AKŞEMSEDDİN BİLİM VE SANAT MERKEZİ	BAL ARISI (APİS MELLİFERA) EKTOPARAZİTİ VARROA'NIN (VARROA DESTRUCTOR) GÖRÜNTÜ İŞLEME TEKNOLOJİSİYLE BELİRLENMESİ	BİYOLOJİ	Tarım ve Hayvancılık Teknolojileri	KAYSERİ	BÖLGE BİRİNCİLİĞİ
290	1689B012424180	A***** Ö*******	Ö**** Ş*****	KAYSERİ ÖZEL BİLFEN KAYSERİ ANADOLU LİSESİ	ATOPİK DERMATİT HASTALARI İÇİN ÖZEL BİR YÖNTEM İLE ANTİMİKROBİYAL KIYAFET ÜRETİMİ	BİYOLOJİ	Halk Sağlığı ve Koruyucu Sağlık Hizmetleri	KAYSERİ	BÖLGE BİRİNCİLİĞİ
291	1689B012426124	A**** S**** G*****	G**** K****************	KAYSERİ ÖZEL BİLFEN KAYSERİ FEN LİSESİ	YAPAY ZEKA İLE İNSAN GAİTASINDAN OBEZİTE TANISI, BİYOBELİRTEÇ BELİRLENMESİ	BİYOLOJİ	Yapay Zekâ	KAYSERİ	BÖLGE BİRİNCİLİĞİ
292	1689B012428154	D*** K*****<br>T**** E**** S*****	S**** D******	SİVAS SİVAS BURUCİYE BİLİM VE SANAT MERKEZİ	KARABAŞ OTU (LAVANDULA STOECHAS) YAĞININ HT-22 HÜCRELERİNDE PENTİLENTATRAZOL İLE OLUŞTURULAN NÖRONAL HASARLANMA ÜZERİNE ETKİSİNİN ARAŞTIRILMASI	BİYOLOJİ	Halk Sağlığı ve Koruyucu Sağlık Hizmetleri	KAYSERİ	BÖLGE İKİNCİLİĞİ
293	1689B012412308	S**** Ç**** A******<br>İ****** E*** A****<br>E**** D****	A***** I*** K*******	KAYSERİ ŞEHİT HACIBEY KAYA ANADOLU LİSESİ	KAYSERİ İLİ SARIOĞLAN İLÇESİNDE YETİŞTİRİLEN ŞEKER PANCARININ İKLİM DEĞİŞİKLİĞİNE TEPKİSİ: KISITLI SULAMA PROGRAMLARI VERİM İLİŞKİSİ	BİYOLOJİ	Ekolojik Denge	KAYSERİ	BÖLGE ÜÇÜNCÜLÜĞÜ
294	1689B012402156	K******* Ç****<br>E*** E**** E****<br>Z***** E** E***	O**** K******	SİVAS TED SİVAS KOLEJİ ÖZEL ANADOLU LİSESİ	TÜRK TARİHİNDE SAVAŞLARIN KAZANILMASI VE KAYBEDİLMESİNDEKİ COĞRAFİ ETKENLER	COĞRAFYA	Bilim Tarihi ve Felsefesi	KAYSERİ	BÖLGE BİRİNCİLİĞİ
295	1689B012418370	E***** D****	D**** K******	AKSARAY AKSARAY BİLİM VE SANAT MERKEZİ	DEPREMZEDELERİN 6 ŞUBAT DEPREMİNE YÖNELİK MEKAN ALGISININ İNCELENMESİ: HATAY ÖRNEKLEMİ	COĞRAFYA	Doğal Afetler ve Afet Yönetimi	KAYSERİ	BÖLGE İKİNCİLİĞİ
296	1689B012429214	N**** Y**** U****<br>B**** T*****	H**** G******	SİVAS SİVAS BİLİM VE SANAT MERKEZİ	ENDEMİK BİTKİLERİN BİLİMSEL İLLÜSTRASYONU	COĞRAFYA	Çevre ve Çevreyi Koruma	KAYSERİ	BÖLGE ÜÇÜNCÜLÜĞÜ
297	1689B012425858	S*** D***<br>F**** H**** B*****<br>B*** Ç****	H****** E******	KAYSERİ KOCASİNAN MESLEKİ VE TEKNİK ANADOLU LİSESİ	HER YUDUMDA DEĞERLENİYORUM	DEĞERLER EĞİTİMİ	Değerler Eğitimi	KAYSERİ	BÖLGE BİRİNCİLİĞİ
298	1689B012429305	P**** I****<br>H***** Ç*******<br>N**** M********	F*** A****	KAYSERİ KOCASİNAN MESLEKİ VE TEKNİK ANADOLU LİSESİ	ULU ÇINARLARDAN GENÇ FİDANLARA KUŞAKLARARASI ETKİLEŞİM PROGRAMI	DEĞERLER EĞİTİMİ	Değerler Eğitimi	KAYSERİ	BÖLGE İKİNCİLİĞİ
299	1689B012422681	R**** G****<br>R****** H*****	İ**** M***	KAYSERİ KAYSERİ KIZ ANADOLU İMAM HATİP LİSESİ	ALİ KAŞİF İLE BİLİMDEN DEĞERLERE	DEĞERLER EĞİTİMİ	Bilim Tarihi ve Felsefesi	KAYSERİ	BÖLGE ÜÇÜNCÜLÜĞÜ
300	1689B012411662	H**** S*****<br>İ*** K******<br>A**** Ü***	R***** U*****	NEVŞEHİR NEVŞEHİR H.AVNİ İNCEKARA FEN LİSESİ	FLORESAN MOLEKÜLLER VE ISLATMAMA KARARSIZLIĞINI TEMEL ALARAK FİZİKSEL OLARAK KLONLANAMAYAN FONKSİYON GELİŞTİRME	FİZİK	Malzeme Bilimi ve Nanoteknoloji	KAYSERİ	BÖLGE BİRİNCİLİĞİ
301	1689B012432156	M******* S***** T*****	A**** Y*****	KAYSERİ SÜMER FEN LİSESİ	ELEKTROMANYETİK DARBE	FİZİK	Milli Teknoloji Hamlesi	KAYSERİ	BÖLGE BİRİNCİLİĞİ
302	1689B012431021	F**** M***** U***<br>E****** E****<br>Ş**** A*** B*****	M****** E****	AKSARAY ŞEHİT NURULLAH SABIRER FEN LİSESİ	RÜZGARA KARŞI HEP VARIM İLE RÜZGAR ENERJİ SANTRALLERİNİN VERİMLİLİĞİNİN ARTIRILMASI	FİZİK	Yenilenebilir Enerji	KAYSERİ	BÖLGE İKİNCİLİĞİ
303	1689B012400581	B******* U***	S**** Ç***	NEVŞEHİR ÖZKONAK HACI HALİL TÜRKKAN ANADOLU LİSESİ	MORÖTESİ ARMATÜRLER İLE KAMUSAL VE ÖZEL KAPALI MEKANLARDA HİJYEN SAĞLANMASI	FİZİK	Salgın Hastalıklar ve Salgınla Mücadele	KAYSERİ	BÖLGE ÜÇÜNCÜLÜĞÜ
304	1689B012428597	B**** G*****	U*** G****	KAYSERİ ÇETİN ŞEN BİLİM VE SANAT MERKEZİ	SÜPERHİDROFOBİK KAPLAMA KULLANILARAK ISLANMAYAN YARA BANTLARININ ÜRETİLMESİ	KİMYA	Malzeme Bilimi ve Nanoteknoloji	KAYSERİ	BÖLGE BİRİNCİLİĞİ
305	1689B012418044	R****** A****<br>A**** A*****<br>D**** K**	E**** K*******	SİVAS SİVAS FEN LİSESİ	SANAL LABORATUVAR KONUSUNDA ÖĞRENCİLERİN FARKINDALIKLARININ İNCELENMESİ	KİMYA	Artırılmış, Sanal ve Karma Gerçeklik	KAYSERİ	BÖLGE İKİNCİLİĞİ
306	1689B012422099	S***** N*** Y****<br>A*** G****<br>M***** G****	Z***** Ş******	KAYSERİ TED KAYSERİ KOLEJİ VAKFI ÖZEL LİSESİ	İNSAN SAÇI VE HAYVAN KILI ATIKLARININ GÜBRE YAPIMINDA KULLANILMASI.	KİMYA	Atık Yönetimi ve Geri Dönüşüm	KAYSERİ	BÖLGE ÜÇÜNCÜLÜĞÜ
307	1689B012435196	E*** Ö******	S***** A*******	KAYSERİ ÇETİN ŞEN BİLİM VE SANAT MERKEZİ	BÜTÜN ASALLARI KAPSAYAN YENİ BİR BÖLÜNEBİLME KURALI	MATEMATİK	Özgün Algoritma Tasarımı	KAYSERİ	BÖLGE BİRİNCİLİĞİ

SIRA NO	BAŞVURU NO	ÖĞRENCİ ADI SOYADI	DANIŞMAN ADI SOYADI	OKULU	PROJENİN ADI	ANA ALANI	TEMATİK ALANI	BÖLGESİ	BÖLGE AŞAMASI SONUCU
308	1689B012415493	Z***** S* S****<br>D**** Ö****<br>I**** K************	H**** T****	KAYSERİ MELİKGAZİ MUSTAFA EMİNOĞLU ANADOLU LİSESİ	RASYONELSİZ	MATEMATİK	Özgün Algoritma Tasarımı	KAYSERİ	BÖLGE İKİNCİLİĞİ
309	1689B012417431	İ**** K************<br>H**** K*****	H**** T****	KAYSERİ MELİKGAZİ MUSTAFA EMİNOĞLU ANADOLU LİSESİ	ÜÇGENİN SAKLI ÇEMBERLERİ	MATEMATİK	STEAM (Fen, Teknoloji, Mühendislik, Sanat ve Matematik)	KAYSERİ	BÖLGE ÜÇÜNCÜLÜĞÜ
310	1689B012433039	C***** K*****<br>A*** Y****	Ş***** A**** T*****	KAYSERİ ÇETİN ŞEN BİLİM VE SANAT MERKEZİ	LİSE ÖĞRENCİLERİNİN ÖNYARGILARININ ÇEŞİTLİ FAKTÖRLERE GÖRE İNCELENMESİ VE OYUNLAŞTIRMA YÖNTEMİ İLE FARKINDALIKLARININ ARTIRILMASI	PSİKOLOJİ	Yapay Zekâ	KAYSERİ	BÖLGE BİRİNCİLİĞİ
311	1689B012418897	A*** S****	O** D****	SİVAS SİVAS BURUCİYE BİLİM VE SANAT MERKEZİ	REHBERLİK VE PSİKOLOJİK DANIŞMA HİZMETLERİNİN ETKİLİLİĞİNİ ARTIRMAK: ÇEVRİMİÇİ-PDR MOBİL UYUMLU WEB UYGULAMASI GELİŞTİRİLMESİ	PSİKOLOJİ	Dijital Dönüşüm	KAYSERİ	BÖLGE İKİNCİLİĞİ
312	1689B012417827	İ*** K***<br>B******* E******<br>N*** N***** İ***	E*** K*******	SİVAS PROF. DR. NECATİ ERŞEN SOSYAL BİLİMLER LİSESİ	BİR DÖNÜM NOKTASI: KARİYER YOLCULUĞUM	PSİKOLOJİ	Bilinçli Farkındalık ve Kariyer Bilinci	KAYSERİ	BÖLGE ÜÇÜNCÜLÜĞÜ
313	1689B012410889	N*** U*****<br>S**** G** K****	S*** Ş****	KAYSERİ OSMAN ULUBAŞ KAYSERİ FEN LİSESİ	LİSE ÖĞRENCİLERİNDE TAKLİT ÜRÜN KULLANIMIN SOSYAL KİMLİK OLUŞUMUNA ETKİSİ	SOSYOLOJİ	Değerler Eğitimi	KAYSERİ	BÖLGE BİRİNCİLİĞİ
314	1689B012433850	Ç**** A*** D*****	D**** K*****	SİVAS SİVAS BİLİM VE SANAT MERKEZİ	LİSE ÖĞRENCİLERİNİN OKULLARINDA ALDIKLARI EĞİTİMLERİN YAŞAM BECERİLERİNE KATKISINA YÖNELİK GÖRÜŞLERİNİN İNCELENMESİ	SOSYOLOJİ	Bilinçli Farkındalık ve Kariyer Bilinci	KAYSERİ	BÖLGE BİRİNCİLİĞİ
315	1689B012435081	A*** E** Y****<br>Y***** S********	N**** F********	KAYSERİ TED KAYSERİ KOLEJİ VAKFI ÖZEL LİSESİ	BİREYLERİ BOŞANMAYA İTEN ETKENLERİN HUKUKÇULAR PERSPEKTİFİNDEN İNCELENMESİ (KAYSERİ İLİ ÖRNEĞİ)	SOSYOLOJİ	Aile İçi İletişim	KAYSERİ	BÖLGE İKİNCİLİĞİ
316	1689B012433131	S***** U***	O** D****	SİVAS SİVAS BURUCİYE BİLİM VE SANAT MERKEZİ	"NORMAL" VE " ÖZEL YETENEKLİ ORTAÖĞRETİM GENÇLİĞİNDE 'MAKUL', 'MUTEBER' V E 'MEŞRU' OLANIN SINIRLARI SİVAS ÖRNEĞİ	SOSYOLOJİ	Değerler Eğitimi	KAYSERİ	BÖLGE ÜÇÜNCÜLÜĞÜ
317	1689B012406252	M****** K*****<br>H****** T**** A***<br>M***** B**	M****** T*****	AKSARAY ABDÜLHAMİD HAN FEN LİSESİ	DERİN ÖĞRENME (DEEP LEARNING) İLE AKSARAY MÜZESİNDEKİ MEZAR TAŞLARININ SINIFLANDIRILMASI VE OKUNMASI	TARİH	Kültürel Miras	KAYSERİ	BÖLGE BİRİNCİLİĞİ
318	1689B012401488	A**** K************<br>S****** N** O****	C**** B*****	KAYSERİ ÖZEL BAHÇEŞEHİR ANADOLU LİSESİ	TARİHİ YAPILARDA UNUTULAN TAŞÇI İŞARETLERİNİN STEM'İ(KAYSERİ GEVHER NESİBE DARÜŞŞİFASI ÖRNEĞİ)	TARİH	Kültürel Miras	KAYSERİ	BÖLGE İKİNCİLİĞİ
319	1689B012429176	F**** M***** K*******	N****** K****	KAYSERİ ÇETİN ŞEN BİLİM VE SANAT MERKEZİ	HARİTALI KUTU OYUNLARININ CUMHURİYET TARİHİNİ ÖĞRENMEYE ETKİSİ: "TARİHE SOR" OYUNU	TARİH	Oyun ve Oyunlaştırma	KAYSERİ	BÖLGE ÜÇÜNCÜLÜĞÜ
320	1689B012408965	G***** Ü***<br>B**** K****<br>M***** M****	M***** A** Ü****	KAYSERİ MERKEZ MESLEKİ VE TEKNİK ANADOLU LİSESİ	3D KORUMA İHA	TEKNOLOJİK TASARIM	Milli Teknoloji Hamlesi	KAYSERİ	BÖLGE BİRİNCİLİĞİ
321	1689B012436909	K***** E*** K*******<br>Y**** D****<br>M***** H**** U***	Y**** İ****	KAYSERİ NUH MEHMET KÜÇÜKÇALIK ANADOLU LİSESİ<br>İSTANBUL ARAŞTIRMA GELİŞTİRME EĞİTİM VE UYGULAMA MERKEZİ LİSESİ<br>KAYSERİ SÜMER FEN LİSESİ	DEPREM SONRASI TERMAL KAMERALI ARAMA KURTARMA DRONE SİSTEMİ	TEKNOLOJİK TASARIM	Doğal Afetler ve Afet Yönetimi	KAYSERİ	BÖLGE BİRİNCİLİĞİ
322	1689B012415080	H**** Y**** Ş****	D**** E***	SİVAS SİVAS BİLİM VE SANAT MERKEZİ	DERİN ÖĞRENME MİMARİLERİ KULLANILARAK GERÇEK ZAMANLI YANGIN TESPİT SİSTEMİ	TEKNOLOJİK TASARIM	Yapay Zekâ	KAYSERİ	BÖLGE İKİNCİLİĞİ
323	1689B012420587	M*** Ç*****<br>F**** G***** G******<br>M***** Ö******	C***** B*******	NİĞDE REMİDE YILMAZ ATABEK ANADOLU LİSESİ	GÖRME ARKADAŞIM	TEKNOLOJİK TASARIM	Giyilebilir Teknolojiler	KAYSERİ	BÖLGE ÜÇÜNCÜLÜĞÜ
324	1689B012419744	K**** Y**** T****	S***** S****	SİVAS SİVAS BURUCİYE BİLİM VE SANAT MERKEZİ	ÇOCUK KİTAPLARI İÇİN OLUMSUZ İÇERİK AVCISI	TÜRK DİLİ VE EDEBİYATI	Dijital Dönüşüm	KAYSERİ	BÖLGE BİRİNCİLİĞİ
325	1689B012415986	G****** C*****<br>E** N** Y*****	N**** Y*******	SİVAS NECİP FAZIL KISAKÜREK MESLEKİ VE TEKNİK ANADOLU LİSESİ	EZBER BOZAN	TÜRK DİLİ VE EDEBİYATI	Dijital Dönüşüm	KAYSERİ	BÖLGE İKİNCİLİĞİ
326	1689B012418316	Z***** E** Ş****<br>C***** Ü***	Ş***** A***	KAYSERİ ÇETİN ŞEN BİLİM VE SANAT MERKEZİ	"ÇANAKKALE ŞEHİTLERİNE" ŞİİRİNİ DAHA İYİ ANLAMAK İÇİN DİJİTAL BİR MATERYAL ÖNERİSİ: KELİMELERDE KALEMİZ	TÜRK DİLİ VE EDEBİYATI	Dijital Oyun Tasarımı	KAYSERİ	BÖLGE ÜÇÜNCÜLÜĞÜ
327	1689B012410555	Y**** S**** K****<br>M**** E*** G*******<br>O**** M**** K****	Y****** E****	NİĞDE NİĞDE YUNUS EMRE ANADOLU LİSESİ	FİZİK TEDAVİ GEREKSİNİMİ DUYAN ÇOCUKLARIN ÖZEL EĞİTİM VE REHABİLİTASYON MERKEZİ KAYGILARININ AZALTILMASI	YAZILIM	Oyun ve Oyunlaştırma	KAYSERİ	BÖLGE BİRİNCİLİĞİ
328	1689B012405836	E*** Ş****<br>D*** B***********	D**** E***	SİVAS SİVAS BİLİM VE SANAT MERKEZİ	DERİN ÖĞRENME MİMARİLERİ KULLANILARAK MR GÖRÜNTÜLERİ İLE ALZHEİMER HASTALIĞININ TESPİT EDİLMESİ	YAZILIM	Yapay Zekâ	KAYSERİ	BÖLGE İKİNCİLİĞİ
329	1689B012411279	S******* G*****<br>E** A******	D**** E***	SİVAS SİVAS BİLİM VE SANAT MERKEZİ	DERİN ÖĞRENME MİMARİLERİ KULLANILARAK HAVA ARAÇLARINI GERÇEK ZAMANLI TESPİT EDEBİLEN OTONOM İHA	YAZILIM	Yapay Zekâ	KAYSERİ	BÖLGE ÜÇÜNCÜLÜĞÜ
330	1689B012402380	A*** S****<br>T**** Y*****	S**** Z**** G****	ANTALYA NAMIK KARAMANCI FEN LİSESİ	MİKROAKIŞKAN ÇİP ÜZERİNDE İNSAN KAN-BEYİN BARİYERİNİN MODELLENMESİ, YEŞİL SENTEZ KUANTUM NOKTALARIN GEÇİŞİNİN VE TOKSİSİTESİNİN ARAŞTIRILMASI	BİYOLOJİ	Genetik ve Biyoteknoloji	KONYA	BÖLGE BİRİNCİLİĞİ
331	1689B012410863	P**** Ö*****<br>K******* Ş**** Ö*******	G***** Y*******	AFYONKARAHİSAR SÜLEYMAN DEMİREL FEN LİSESİ	SİDERİTİS AKMANİİ (DAĞ ÇAYI)'DEN YEŞİL SENTEZLE ELDE EDİLEN ÇİNKO OKSİT NANOPARTİKÜLÜNÜN DROSOPHİLA MELANOGASTER ÜZERİNDE DAVRANIŞ TOKSİSİTESİ	BİYOLOJİ	Genetik ve Biyoteknoloji	KONYA	BÖLGE BİRİNCİLİĞİ
332	1689B012426664	Z***** E**** G****<br>V**** Ş******<br>T**** G*****	H***** Ü******	ANTALYA YUSUF ZİYA ÖNER FEN LİSESİ<br>ANTALYA ADEM-TOLUNAY ANADOLU LİSESİ	PHYSARUM POLYCEPHALUM KULLANILARAK GELİŞTİRİLEN ALGORİTMA İLE BEYİN CERRAHİSİNE YENİ BİR BAKIŞ	BİYOLOJİ	Özgün Algoritma Tasarımı	KONYA	BÖLGE İKİNCİLİĞİ
333	1689B012423436	B***** Ç********	C** Y******	ANTALYA ÖZEL KONYAALTI BAHÇEŞEHİR KOLEJİ FEN VE TEKNOLOJİ LİSESİ	BİRA MAYASINDA CRISPR KULLANILARAK TELOMER KISALMASINI ÖNLEMEK: BİR MODEL ÇALIŞMA	BİYOLOJİ	Genetik ve Biyoteknoloji	KONYA	BÖLGE ÜÇÜNCÜLÜĞÜ
334	1689B012429648	B**** Y*****<br>F**** N** Ö*****<br>B**** U***	Y****** Ö******	ISPARTA TENZİLE ERDOĞAN ANADOLU LİSESİ<br>ISPARTA 15 TEMMUZ ŞEHİTLER ANADOLU LİSESİ	MERMER OCAKLARINDA, GÖRSELLİĞİ BOZULMUŞ ALANLARDA ARONİA MELANOCARPA ÜRETİMİNİN TEŞVİK EDİLMESİYLE OLUŞACAK ÇEVRECİ ÜRETİM MODELİNİN İNCELENMESİ	COĞRAFYA	Ekolojik Denge	KONYA	BÖLGE BİRİNCİLİĞİ
335	1689B012429664	E******* E*** H****<br>N** D*****	A*** Y******* Ş****	ANTALYA GÖYNÜK FEN LİSESİ	DEPREM SONRASI TARAMA TESPİT VE AFETE MÜDAHALE İHA'SI (ASPAR)	COĞRAFYA	Doğal Afetler ve Afet Yönetimi	KONYA	BÖLGE BİRİNCİLİĞİ
336	1689B012414932	M***** B**** B*****<br>Ş****** S***	Ş***** Ç****	KONYA KONYA TÜRK TELEKOM SOSYAL BİLİMLER LİSESİ	COĞRAFYADA TÜRK İZİ	COĞRAFYA	Kültürel Miras	KONYA	BÖLGE İKİNCİLİĞİ
337	1689B012423873	H****** K***<br>Y**** K******	A**** E**	ISPARTA IŞIKKENT ANADOLU İMAM HATİP LİSESİ	YAPAY ZEKA EKOSİSTEMİNDE COĞRAFYA	COĞRAFYA	Dijital Dönüşüm	KONYA	BÖLGE ÜÇÜNCÜLÜĞÜ
338	1689B012416289	A*** F**** H******<br>S****** D****<br>Z*** T**** S*****	N******* K****	ISPARTA ISPARTA GAZİ SOSYAL BİLİMLER LİSESİ	BİZİM OYUNLARIMIZ BİZİM DEĞERLERİMİZ	DEĞERLER EĞİTİMİ	Kültürel Miras	KONYA	BÖLGE BİRİNCİLİĞİ

SIRA NO	BAŞVURU NO	ÖĞRENCİ ADI SOYADI	DANIŞMAN ADI SOYADI	OKULU	PROJENİN ADI	ANA ALANI	TEMATİK ALANI	BÖLGESİ	BÖLGE AŞAMASI SONUCU
339	1689B012410376	R******* G*****<br>N*** N** Ö*****	G***** T**** G*****	KARAMAN KARAMAN BİLİM VE SANAT MERKEZİ	ANADOLU HALI VE KİLİMLERİNDEKİ MOTİFLERİN GİZLİ MESAJLARI VE BU MOTİFLERİN GÜNÜMÜZ EŞYALARINA YANSIMASI	DEĞERLER EĞİTİMİ	Kültürel Miras	KONYA	BÖLGE İKİNCİLİĞİ
340	1689B012415120	Z***** H**** Ç****<br>R*** K*****<br>Y***** E******	G***** M****	KARAMAN KARAMAN BİLİM VE SANAT MERKEZİ	ANADOLU'NUN SON EMANETLERİ: KARAMAN PİR AHMET (KALE) CAMİİ KAPISINDAKİ EVLİYA ÇELEBİ İMZASI	DEĞERLER EĞİTİMİ	Kültürel Miras	KONYA	BÖLGE ÜÇÜNCÜLÜĞÜ
341	1689B012402391	S****** İ*** Y****	S**** Z**** G****	ANTALYA NAMIK KARAMANCI FEN LİSESİ	YÜKSEK HASSASİYETLİ, DÜŞÜK MALİYETLİ FİBER OPTİK JİROSKOPİK KUANTUM SENSÖRÜ GELİŞTİRİLEREK KUANTUM PUSULASI YAPIMINDA KULLANILMASI	FİZİK	Milli Teknoloji Hamlesi	KONYA	BÖLGE BİRİNCİLİĞİ
342	1689B012418822	N**** Ş*****<br>A********* O*****<br>S******* Ü***	E**** C*****	BURDUR RECEP TAYYİP ERDOĞAN ANADOLU İMAM HATİP LİSESİ	LAZER İLE İLETİŞİM KURMA	FİZİK	STEAM (Fen, Teknoloji, Mühendislik, Sanat ve Matematik)	KONYA	BÖLGE İKİNCİLİĞİ
343	1689B012423432	N**** Z***** A****<br>Z***** S*****<br>Y**** Ç*******	M**** M*******	AFYONKARAHİSAR DUMLUPINAR BİLİM VE SANAT MERKEZİ	YÜKSEK VERİMLİ HAVA FİLTRELERİ İÇİN SES DALGALARININ FİZİKSEL ÖZELLİKLERİNİN ARAŞTIRILMASI VE PROTATİP TASARIM ÜZERİNE ETKİSİ	FİZİK	Görüntü ve Ses Tanıma Teknolojileri	KONYA	BÖLGE ÜÇÜNCÜLÜĞÜ
344	1689B012401492	A*** G****<br>A*** D**********	S**** Z**** G****	ANTALYA NAMIK KARAMANCI FEN LİSESİ	BOROFEN SENTEZİ VE SENTEZLENEN BOROFENİN HİDROJEN DEPOLAMA KAPASİTESİNİN KUVARS KRİSTAL MİKROBALANS YÖNTEMİYLE TESPİT EDİLMESİ	KİMYA	Hidrojen Enerjisi	KONYA	BÖLGE BİRİNCİLİĞİ
345	1689B012421494	B***** U***<br>I**** A*****<br>H**** N**** K***	A*** B****	ANTALYA HASAN ÇOLAK ANADOLU LİSESİ<br>ANTALYA ÖZEL BİLFEN ANTALYA FEN LİSESİ	PORTULACA GRANDİFLORA HOOK İLE İKLİM DEĞİŞİKLİĞİ VE KURAKLIK SENARYOLARINDA BİLE SÜRDÜRÜLEBİLİR YENİ NESİL PLASTİK	KİMYA	Küresel Isınma ve İklim Değişikliği	KONYA	BÖLGE İKİNCİLİĞİ
346	1689B012429916	K******* Ö*******<br>S*** N** Ç******	Ş***** Ç****	ANTALYA ÖZEL ANTALYA MAYA ANADOLU LİSESİ	EKO-SENSÖR PAKETLEME: DOĞAL KAYNAKLARDAN ELDE EDİLEN H'YA DUYARLI BİYOPLASTİK FİLMLER	KİMYA	Malzeme Bilimi ve Nanoteknoloji	KONYA	BÖLGE ÜÇÜNCÜLÜĞÜ
347	1689B012439033	B**** A** T*****<br>E*** D*****<br>E***** K****	İ******* Ö*****	ANTALYA ANTALYA BİLİM VE SANAT MERKEZİ<br>ANTALYA ANTALYA TÜRKİYE ODALAR VE BORSALAR BİRLİĞİ FEN LİSESİ	BASAMAKLANDIRILMIŞ KRİPTOLOJİ: BİR SAYMA PROBLEMİNDEN SİBER GÜVENLİĞE	MATEMATİK	Siber Güvenlik	KONYA	BÖLGE BİRİNCİLİĞİ
348	1689B012418034	H********* A*<br>A***** A*****	K***** K*****	ANTALYA ÖZEL ANTALYA MAYA FEN VE TEKNOLOJİ LİSESİ	RNA TABANLI GENETİK KODLARIN MATEMATİKSEL ŞİFRELEME SİSTEMLERİNE UYGULANMASI: YENİLİKÇİ BİR VERİ GÜVENLİĞİ YAKLAŞIMI	MATEMATİK	STEAM (Fen, Teknoloji, Mühendislik, Sanat ve Matematik)	KONYA	BÖLGE İKİNCİLİĞİ
349	1689B012418763	A**** E*** D******<br>F**** S*** B********	Ö**** K*******	ANTALYA MURATPAŞA BİLİM VE SANAT MERKEZİ	BİYOKRİPTO	MATEMATİK	Özgün Algoritma Tasarımı	KONYA	BÖLGE ÜÇÜNCÜLÜĞÜ
350	1689B012419987	B******* U***<br>A*** Ç****	S**** Ç****	ANTALYA ALANYA BİLİM VE SANAT MERKEZİ	ÖZEL YETENEKLİ ÖĞRENCİLERİN GÖZÜNDEN BİLİM İNSANLARI	PSİKOLOJİ	Bilim Tarihi ve Felsefesi	KONYA	BÖLGE BİRİNCİLİĞİ
351	1689B012429057	B***** S****** A*****<br>N*** B*****	M***** K******* Ç*****	KARAMAN KARAMAN BİLİM VE SANAT MERKEZİ	DEPREMZEDE ÖĞRENCİLERİN ALGILADIKLARI SOSYAL DESTEK DÜZEYİ İLE DUYGU DÜZENLEME BECERİLERİ ARASINDAKİ İLİŞKİNİN İNCELENMESİ	PSİKOLOJİ	Göç ve Uyum	KONYA	BÖLGE İKİNCİLİĞİ
352	1689B012429190	M****** V** W*****<br>E*** F**** E***	M****** A****	ANTALYA ALANYA BİLİM VE SANAT MERKEZİ	GÜRÜLTÜ TÜRLERİNİN BİLİM VE SANAT MERKEZİ BİREYSEL YETENEKLERİ FARKETTİRME-2 PROGRAMI ÖĞRENCİLERİNİN ODAKLANMASINA ETKİSİNİN İNCELENMESİ	PSİKOLOJİ	Görsel ve İşitsel Sanatlar	KONYA	BÖLGE ÜÇÜNCÜLÜĞÜ
353	1689B012410464	K**** K****** B****<br>M***** S***	S**** E****	AFYONKARAHİSAR IŞIKLAR ÇOK PROGRAMLI ANADOLU LİSESİ	ERKEN YAŞTA EVLENMİŞ KADINLARIN PSİKOSOSYAL DENEYİMLERİ ÜZERİNE FENOMENOLOJİK BİR İNCELEME: AFYONKARAHİSAR ÖRNEĞİ	SOSYOLOJİ	Aile İçi İletişim	KONYA	BÖLGE BİRİNCİLİĞİ
354	1689B012420419	E*** N*** Y****<br>G**** B****<br>S***** C***** K******	S**** Ç****	ANTALYA ALANYA BİLİM VE SANAT MERKEZİ	ÖZEL YETENEKLİ ÖĞRENCİLERİN SİBER ZORBALIĞA YÖNELİK METAFORİK ALGILARI	SOSYOLOJİ	Siber Güvenlik	KONYA	BÖLGE İKİNCİLİĞİ
355	1689B012421540	Ş**** Y**** E***<br>Ö*** E*** B**<br>F**** D****	Ö**** D*******	BURDUR MEHMET UZAL SOSYAL BİLİMLER LİSESİ	CUMHURİYETİN KADIN YÜZ(100)LERİ	SOSYOLOJİ	Değerler Eğitimi	KONYA	BÖLGE ÜÇÜNCÜLÜĞÜ
356	1689B012415029	M**** Y**** K****<br>A*** I**** A*******<br>A***** M**** O***	M******* Ö*****	AFYONKARAHİSAR DUMLUPINAR BİLİM VE SANAT MERKEZİ	LOZAN ANTLAŞMASI DİJİTAL PLATFORMU	TARİH	Dijital Dönüşüm	KONYA	BÖLGE BİRİNCİLİĞİ
357	1689B012411183	D*** N****<br>M**** B*****	S**** A****	ANTALYA ANTALYA BİLİM VE SANAT MERKEZİ	TOPOGRAFİ VE MODELLEME IŞIĞINDA PİRİ REİS'İN ANTALYA'SI MÜZESİ	TARİH	Bilim Tarihi ve Felsefesi	KONYA	BÖLGE İKİNCİLİĞİ
358	1689B012418707	A***** E****<br>İ*** T*****	S**** A****	ANTALYA ANTALYA BİLİM VE SANAT MERKEZİ	KİTABEMİ ÇİZİ(YORUM) ÖYKÜLÜ(YORUM) ATALARIMI TANI(YORUM)	TARİH	Kültürel Miras	KONYA	BÖLGE ÜÇÜNCÜLÜĞÜ
359	1689B012402361	Ç**** G*****<br>A***** C**<br>M****** E** S*****	İ****** K***	ANTALYA ANTALYA BİLİM VE SANAT MERKEZİ	BAKIM VE TAMİR İÇİN UZAKTAN KONTROL EDİLEBİLEN GÖRÜNTÜ İŞLEME DESTEKLİ ROBOT KOL	TEKNOLOJİK TASARIM	STEAM (Fen, Teknoloji, Mühendislik, Sanat ve Matematik)	KONYA	BÖLGE BİRİNCİLİĞİ
360	1689B012413945	A** A***** G*****<br>M****** S**** A********<br>K**** Ç******	A******* A******	ANTALYA ÖZEL BİLFEN ANTALYA FEN LİSESİ<br>İSTANBUL ÖZEL BİLFEN ÜSKÜDAR FEN LİSESİ	HAVUZLARDAN BULAŞAN SALGIN HASTALIKLARIN ÖNLENMESİNE YÖNELİK OTONOM ROBOT PROJESİ (CLEPOS)	TEKNOLOJİK TASARIM	Salgın Hastalıklar ve Salgınla Mücadele	KONYA	BÖLGE BİRİNCİLİĞİ
361	1689B012415023	B******* K*******<br>B**** E** T*****<br>G***** K*********	F**** İ***	KONYA KARATAY FEN LİSESİ	NESNELERİN İNTERNETİ TABANLI SOKAK HAYVANLARI İÇİN AKILLI MAMA İSTASYONU-"AKMİS"	TEKNOLOJİK TASARIM	Nesnelerin İnterneti	KONYA	BÖLGE BİRİNCİLİĞİ
362	1689B012411121	T*** D****<br>E*** U***<br>K**** Y**** E****	S**** A****	ANTALYA ANTALYA BİLİM VE SANAT MERKEZİ	E-SOS: BİTKİ TAKİP VE KURTARMA SİSTEMİ	TEKNOLOJİK TASARIM	Tarım ve Hayvancılık Teknolojileri	KONYA	BÖLGE İKİNCİLİĞİ
363	1689B012417892	E*** U****	C** Y******	ANTALYA ÖZEL KONYAALTI BAHÇEŞEHİR KOLEJİ FEN VE TEKNOLOJİ LİSESİ	ARAÇ VE GEÇİT YÜKSEKLİĞİ UYGUNLUK KONTROL CİHAZI	TEKNOLOJİK TASARIM	Akıllı Ulaşım Sistemleri	KONYA	BÖLGE ÜÇÜNCÜLÜĞÜ
364	1689B012424063	Z***** A*****<br>M***** Ş*****	F**** D****	ANTALYA MURATPAŞA BİLİM VE SANAT MERKEZİ	KARAKTER DÜZEYİNDE N-GRAM YÖNTEMİ İLE KELİMETRE	TÜRK DİLİ VE EDEBİYATI	Yapay Zekâ	KONYA	BÖLGE BİRİNCİLİĞİ
365	1689B012434979	S**** A****	E*** K****	AFYONKARAHİSAR EMİRDAĞ AZİZİYE ANADOLU LİSESİ	KARAGÖZ FENO OLUYOR	TÜRK DİLİ VE EDEBİYATI	Türk Dili ve Lehçeleri	KONYA	BÖLGE İKİNCİLİĞİ
366	1689B012429272	A***** A******<br>F************ C**********	S**** B*****	KARAMAN KARAMAN LİSESİ	YABANCILARA STORİGAMİ İLE TÜRKÇE ÖĞRETİMİNDE B2 SEVİYESİNDE HİKâYE OLUŞTURMA: KARAMAN LİSESİ ÖRNEĞİ	TÜRK DİLİ VE EDEBİYATI	Dil ve Edebiyat	KONYA	BÖLGE ÜÇÜNCÜLÜĞÜ
367	1689B012415216	T**** Y**** D*********<br>E**** A******* D*********	S******* E******	ISPARTA TED ISPARTA KOLEJİ ÖZEL LİSESİ	DERİN ÖĞRENME İLE 3B SİMÜLASYONLU TORASİK BİYOPSİ VE PULMONAR CERRAHİ YARDIM UYGULAMASI: 3D PULMOSIM	YAZILIM	Yapay Zekâ	KONYA	BÖLGE BİRİNCİLİĞİ
368	1689B012413574	U*** K**** Ö***	H***** K*****	ANTALYA ANTALYA BİLİM VE SANAT MERKEZİ	İŞİTSEL UYARANLARIN FMRI'YLA ELDE EDİLEN ÇIKTILARININ DERİN ÖĞRENME YÖNTEMLERİYLE KONUŞMA YETİSİNİ KAYBETMİŞ KİŞİLERLE İLETİŞİMDE KULLANILMASI	YAZILIM	Yapay Zekâ	KONYA	BÖLGE İKİNCİLİĞİ
369	1689B012400673	Y**** C********	M******* Ç*******	KONYA MAHMUT SAMİ RAMAZANOĞLU ANADOLU İMAM HATİP LİSESİ	TELLCORD (YAPAY ZEKâ TARAFINDAN KOPYALANAN SES İLE İNSAN SESİNİ AYIRAN DERİN ÖĞRENME MODELİ))	YAZILIM	Yapay Zekâ	KONYA	BÖLGE ÜÇÜNCÜLÜĞÜ

SIRA NO	BAŞVURU NO	ÖĞRENCİ ADI SOYADI	DANIŞMAN ADI SOYADI	OKULU	PROJENİN ADI	ANA ALANI	TEMATİK ALANI	BÖLGESİ	BÖLGE AŞAMASI SONUCU
370	1689B012433209	Z***** B**** B******	Ş***** K****	ELAZIĞ ÖZEL ELAZIĞ DOĞA FEN LİSESİ	DENİZ SALYANGOZU KABUĞUNUN DİŞ DOLGU VE İMPLANT MADDESİ OLARAK KULLANIM POTANSİYELİ	BİYOLOJİ	Halk Sağlığı ve Koruyucu Sağlık Hizmetleri	MALATYA	BÖLGE BİRİNCİLİĞİ
371	1689B012434945	İ****** K**** Y*********<br>M******* B****** A*******		ŞANLIURFA ÇEAŞ ŞANLIURFA ANADOLU LİSESİ<br>ŞANLIURFA ŞANLIURFA BİLİM VE SANAT MERKEZİ	SOLANUM LYCOPERSİCUM MEYVELERİNDEN İZOLE EDİLEN EPİKÜTİKÜLER MUMLARIN KARAKTERİZASYONUNUN, ANTİBAKTERİYAL VE UV-C ABSORBSİYON YETENEKLERİNİN ARAŞTIRILMASI	BİYOLOJİ	Biyoçeşitlilik	MALATYA	BÖLGE BİRİNCİLİĞİ
372	1689B012405472	E** E**** E****	A***** B******	DİYARBAKIR REKABET KURUMU CUMHURİYET FEN LİSESİ	MENTHA LONGİFOLİA SUBSP. NOEANA (PUNG-YARPUZ) TÜRÜNÜN ANTİ-AGİNG KREM VE AĞIZ ÇALKALAMA SOLÜSYONU OLARAK POTANSİYELİNİN ARAŞTIRILMASI	BİYOLOJİ	Halk Sağlığı ve Koruyucu Sağlık Hizmetleri	MALATYA	BÖLGE İKİNCİLİĞİ
373	1689B012420500	F**** E*** P*******<br>B**** Y*****<br>Z***** Y*******	H**** C****	ŞANLIURFA ÖZEL MUTLU EĞİTİM MODELİ FEN LİSESİ<br>ŞANLIURFA ÖZEL ŞANLIURFA BAHÇEŞEHİR KOLEJİ FEN LİSESİ	ATIK MSCBM MEDYUMUNDAN AMNİYON SIVISI TÜREVLİ EKSOZOMLARIN K562 HÜCRE HATTI ÜZERİNDE ETKİSİNİN ARAŞTIRILMASI	BİYOLOJİ	Genetik ve Biyoteknoloji	MALATYA	BÖLGE ÜÇÜNCÜLÜĞÜ
374	1689B012409965	M******* F**** Ö*****<br>M***** K*****	A******* K*****	BİNGÖL TÜRKİYE BÜYÜK MİLLET MECLİSİ VAKFI BİNGÖL FEN LİSESİ	AKILLI TARIM BAKIM SİSTEMİ	COĞRAFYA	Tarım ve Hayvancılık Teknolojileri	MALATYA	BÖLGE BİRİNCİLİĞİ
375	1689B012416540	F*** Ç****<br>A*** I***<br>B***** S******	H*** G******	ADIYAMAN EBU SADIK ANADOLU İMAM HATİP LİSESİ	AÇIK UÇLU SORU HAZIRLA(AUSH)	COĞRAFYA	Yapay Zekâ	MALATYA	BÖLGE BİRİNCİLİĞİ
376	1689B012435227	E**** A***<br>E*** U***<br>F**** G** B****	E**** O***	ADIYAMAN TÜRKİYE ODALAR VE BORSALAR BİRLİĞİ KIZ ANADOLU İMAM HATİP LİSESİ	BET-TÜS	COĞRAFYA	Yenilenebilir Enerji	MALATYA	BÖLGE İKİNCİLİĞİ
377	1689B012430540	E******* Ç****<br>A**** E*** G**<br>A*** Ç****	M**** B*****	ELAZIĞ BALAKGAZİ ANADOLU LİSESİ	ETKİLEŞİMLİ, EĞLENCELİ DEPREM BİLİNCİ EĞİTİMİ: DANS, RİTİM, ANİMASYON VE HAREKETLİ SESLİ VR SİMÜLASYONUYLA GÜVENDE KAL	COĞRAFYA	Doğal Afetler ve Afet Yönetimi	MALATYA	BÖLGE ÜÇÜNCÜLÜĞÜ
378	1689B012406123	H***** N***** A***<br>B**** B****<br>H***** K***	Ö*** S*******	DİYARBAKIR ŞEHİT HALİT GÜLSER KIZ ANADOLU İMAM HATİP LİSESİ	KÜÇÜK DOKUNUŞLAR ATÖLYESİ	DEĞERLER EĞİTİMİ	Çevre ve Çevreyi Koruma	MALATYA	BÖLGE BİRİNCİLİĞİ
379	1689B012409948	A**** E** K**<br>M******* S***** B****<br>F**** B********	F**** V****	BİNGÖL TÜRKİYE BÜYÜK MİLLET MECLİSİ VAKFI BİNGÖL FEN LİSESİ	KAYIP EŞYA BULUNMASINDA WEB DESTEĞİ	DEĞERLER EĞİTİMİ	Değerler Eğitimi	MALATYA	BÖLGE BİRİNCİLİĞİ
380	1689B012415368	E*** S*** K***<br>Ö*** A*** D**	Ö**** Ö******	ELAZIĞ ELAZIĞ BİLİM VE SANAT MERKEZİ	LİSE ÖĞRENCİLERİNİN BESİN ETİKETİ OKUMA ALIŞKANLIKLARI VE TUTUMLARININ BELİRLENMESİ	DEĞERLER EĞİTİMİ	Gıda ve Gıda Arzı Güvenliği	MALATYA	BÖLGE İKİNCİLİĞİ
381	1689B012434382	T***** B**** O***<br>T**** E**** Y*******<br>Z***** M****	Ş**** A***	DİYARBAKIR ADNAN MENDERES ANADOLU LİSESİ	ERBANE DOKUNUŞLARI:GEÇMİŞTEN GELECEĞE RİTMİK YOLCULUK	DEĞERLER EĞİTİMİ	Kültürel Miras	MALATYA	BÖLGE ÜÇÜNCÜLÜĞÜ
382	1689B012434794	M******* A** K*****<br>B******** D*******	H***** K*****	BİNGÖL TÜRKİYE BÜYÜK MİLLET MECLİSİ VAKFI BİNGÖL FEN LİSESİ	ORMAN YANGINLARINI SÖNDÜRÜCÜ EM (ELEKTROMANYETİK) MANCINIK	FİZİK	STEAM (Fen, Teknoloji, Mühendislik, Sanat ve Matematik)	MALATYA	BÖLGE BİRİNCİLİĞİ
383	1689B012435133	Ö*** Y**** K**<br>C***** C*****<br>B**** K***	Z***** T*****	MALATYA TED MALATYA KOLEJİ ÖZEL ANADOLU LİSESİ	ATIK PANEL (LCD) İNDİYUM KALAY OKSİT BİLEŞİMİNİN GERİ KAZANIMI	FİZİK	Atık Yönetimi ve Geri Dönüşüm	MALATYA	BÖLGE BİRİNCİLİĞİ
384	1689B012414700	Ç**** I**** G*****		BİNGÖL TÜRKİYE BÜYÜK MİLLET MECLİSİ VAKFI BİNGÖL FEN LİSESİ	SU BORULARINDAN ENERJİ ÜRETİMİ SAĞLAYACAK TÜRBİN	FİZİK	Yenilenebilir Enerji	MALATYA	BÖLGE İKİNCİLİĞİ
385	1689B012436373	M***** A**** T****<br>S****** A******<br>Z***** B*******	A**** B******	BİNGÖL TÜRKİYE BÜYÜK MİLLET MECLİSİ VAKFI BİNGÖL FEN LİSESİ<br>BİNGÖL YUNUS EMRE ANADOLU LİSESİ<br>BİNGÖL KARŞIYAKA ANADOLU LİSESİ	SES DALGALARIYLA ARMONİ TESPİTİ	FİZİK	STEAM (Fen, Teknoloji, Mühendislik, Sanat ve Matematik)	MALATYA	BÖLGE ÜÇÜNCÜLÜĞÜ
386	1689B012423525	Z***** K******<br>A**** A***	M***** Ö*******	ADIYAMAN ADIYAMAN BİLİM VE SANAT MERKEZİ	NİCOTİANA TABACUM İLE AGNP SENTEZİ, KARAKTERİZASYONU VE GIDA PATOJENLERİ ÜZERİNE ETKİSİ	KİMYA	Malzeme Bilimi ve Nanoteknoloji	MALATYA	BÖLGE BİRİNCİLİĞİ
387	1689B012436841	B**** A**	H***** Y***** A*** YO****	ŞANLIURFA ÖZEL ŞANLIURFA TED KOLEJİ ANADOLU LİSESİ	MANTARDAN LEKE ÇIKARICI	KİMYA	Gıda ve Gıda Arzı Güvenliği	MALATYA	BÖLGE İKİNCİLİĞİ
388	1689B012407161	Y***** Y*******<br>T**** K*****<br>Y**** K*** T*******	E**** A****	BİNGÖL TÜRKİYE BÜYÜK MİLLET MECLİSİ VAKFI BİNGÖL FEN LİSESİ	KİMYA LABORATUVARINDAKİ TEHLİKELERİN YAPAY ZEKâ İLE ÖNLENMESİ	KİMYA	Yapay Zekâ	MALATYA	BÖLGE ÜÇÜNCÜLÜĞÜ
389	1689B012432056	A**** F***** S*******<br>A*** B**** Ö******<br>E*** Ç*****	M***** A*****	MALATYA MALATYA BİLİM VE SANAT MERKEZİ	GENELLEŞTİRİLMİŞ SAYI DİZİLERİ İLE WENXIANG DİYAGRAMLARININ TOPOLOJİK İNDEKSLERİ	MATEMATİK	STEAM (Fen, Teknoloji, Mühendislik, Sanat ve Matematik)	MALATYA	BÖLGE BİRİNCİLİĞİ
390	1689B012406411	S*** N** B*****<br>M***** İ****<br>S****** Ö***	B**** K******	MARDİN HATUNİYE MESLEKİ VE TEKNİK ANADOLU LİSESİ	FİBONACCİ POLİNOM MODELLEMESİYLE KISMİ KANSER TEDAVİSİ	MATEMATİK	STEAM (Fen, Teknoloji, Mühendislik, Sanat ve Matematik)	MALATYA	BÖLGE İKİNCİLİĞİ
391	1689B012416419	S*** E***	H**** B********	BİNGÖL YUNUS EMRE ANADOLU LİSESİ	THOMSEN FİGÜRÜ VE ÖZELLİKLERİ	MATEMATİK	STEAM (Fen, Teknoloji, Mühendislik, Sanat ve Matematik)	MALATYA	BÖLGE ÜÇÜNCÜLÜĞÜ
392	1689B012432410	G***** Ö*******<br>B**** N** Ö*******	E****** K*****	MALATYA HACI AVNİ KIZ ANADOLU İMAM HATİP LİSESİ	6 ŞUBAT DEPREMİ SONRASI AKRAN DAYANIŞMALI PSİKOSOSYAL ÇALIŞMALARIN ÖĞRENCİLERİN PSİKOLOJİK İYİ OLUŞ DÜZEYİNE ETKİSİ	PSİKOLOJİ	Doğal Afetler ve Afet Yönetimi	MALATYA	BÖLGE BİRİNCİLİĞİ
393	1689B012410552	S**** K****	E*** C**	ADIYAMAN BESNİ ŞEHİT EREN KUPAL MESLEKİ VE TEKNİK ANADOLU LİSESİ	KAHRAMANMARAŞ DEPREMLERİNİ YAŞAMIŞ LİSE ÖĞRENCİLERİNİN "DEPREM" KAVRAMINA YÖNELİK METAFORİK ALGILARI	PSİKOLOJİ	Doğal Afetler ve Afet Yönetimi	MALATYA	BÖLGE İKİNCİLİĞİ
394	1689B012432612	B****** K******	Ö**** Y****	ADIYAMAN KAHTA ANADOLU LİSESİ	SINAV SİMÜLASYONLARI İLE SINAV STRESİNİ AZALTMA	PSİKOLOJİ	Artırılmış, Sanal ve Karma Gerçeklik	MALATYA	BÖLGE ÜÇÜNCÜLÜĞÜ
395	1689B012400575	M******* S**** B*******	A********** Ç********	BİNGÖL BİNGÖL BİLİM VE SANAT MERKEZİ	DİJİTAL DÜNYA VE HABİTUS: Z KUŞAĞININ KİMLİK OLUŞUM SÜREÇLERİNE DİJİTAL ETKİLEŞİMLERİN DERİNLEMESİNE ETKİSİ	SOSYOLOJİ	Medya Okuryazarlığı	MALATYA	BÖLGE BİRİNCİLİĞİ
396	1689B012430525	F**** A****<br>M*** B**** T****	A**** Ş**** T*****	ŞANLIURFA HADİ KUTLU MESLEKİ VE TEKNİK ANADOLU LİSESİ	MESLEK LİSESİ ÖĞRENCİLERİNDE CHATGPT KULLANIMININ YÜKSEKÖĞRENİM SINAVLARINA HAZIRLIK SÜRECİNDEKİ ROLÜ VE BAŞARIYA ETKİSİ	SOSYOLOJİ	Yapay Zekâ	MALATYA	BÖLGE İKİNCİLİĞİ
397	1689B012428994	E*** C** Y*******<br>İ****** P****	B**** D*****	DİYARBAKIR REKABET KURUMU CUMHURİYET FEN LİSESİ	YURT DIŞINA GİTMEK İSTEYEN ÖĞRENCİLERE YÖNELİK BİLİNÇLİ TERCİHLER İÇİN FARKINDALIK OLUŞTURMA	SOSYOLOJİ	Bilinçli Farkındalık ve Kariyer Bilinci	MALATYA	BÖLGE ÜÇÜNCÜLÜĞÜ
398	1689B012436652	R******* K******<br>İ*** A*** S******	B**** K*****	MALATYA TECDE ANADOLU LİSESİ	ORTAÖĞRETİM ÖĞRENCİLERİNDE SOMUT OLMAYAN KÜLTÜREL MİRAS ALGISI	TARİH	Kültürel Miras	MALATYA	BÖLGE BİRİNCİLİĞİ
399	1689B012426042	İ**** C**** A*<br>H****** O**********	H***** Y***** B******	MARDİN MİDYAT ANADOLU LİSESİ	GİZEMLİ ŞEHİR MATİATE'NİN TURİZME KAZANDIRILMASI	TARİH	Kültürel Miras	MALATYA	BÖLGE İKİNCİLİĞİ
400	1689B012403848	B**** B*******<br>E**** Ç****	A********** Ç********	BİNGÖL BİNGÖL BİLİM VE SANAT MERKEZİ	ANADOLU'NUN KAYIP ŞEHİRLERİ: SANAL ZAMAN YOLCULUĞU (EFES)	TARİH	Artırılmış, Sanal ve Karma Gerçeklik	MALATYA	BÖLGE ÜÇÜNCÜLÜĞÜ

SIRA NO	BAŞVURU NO	ÖĞRENCİ ADI SOYADI	DANIŞMAN ADI SOYADI	OKULU	PROJENİN ADI	ANA ALANI	TEMATİK ALANI	BÖLGESİ	BÖLGE AŞAMASI SONUCU
401	1689B012401837	B**** Ş*****<br>M***** E*** H****	N**** K****	ŞANLIURFA ÇEAŞ ŞANLIURFA ANADOLU LİSESİ	AY'IN EVRELERİNİN 3D MODELLEMESİ	TEKNOLOJİK TASARIM	Astronomi ve Astrofizik	MALATYA	BÖLGE BİRİNCİLİĞİ
402	1689B012412961	M****** Y****<br>M***** S**** A****	M***** Y*****	BİNGÖL TÜRKİYE BÜYÜK MİLLET MECLİSİ VAKFI BİNGÖL FEN LİSESİ	GÖZ HAREKETLERİYLE BİLGİSAYAR KONTROL SİSTEMİ (GHBKS)	TEKNOLOJİK TASARIM	Görüntü ve Ses Tanıma Teknolojileri	MALATYA	BÖLGE BİRİNCİLİĞİ
403	1689B012421970	Ö*** S*****<br>M******* S**** Y*****	E*** P****	ELAZIĞ ELAZIĞ BİLİM VE SANAT MERKEZİ	YAPAY ZEKA DESTEKLİ TARIM ROBOTU	TEKNOLOJİK TASARIM	Tarım ve Hayvancılık Teknolojileri	MALATYA	BÖLGE İKİNCİLİĞİ
404	1689B012435635	O**** B**** E***<br>İ****** Ö****<br>A**** Y**** E*******	H***** K*****	BİNGÖL KARŞIYAKA ANADOLU LİSESİ<br>BİNGÖL 15 TEMMUZ MİLLİ İRADE ANADOLU İMAM HATİP LİSESİ	AGROSTEM-TARIMSAL İLAÇLAMA DRONU	TEKNOLOJİK TASARIM	Tarım ve Hayvancılık Teknolojileri	MALATYA	BÖLGE ÜÇÜNCÜLÜĞÜ
405	1689B012427116	Y**** A****	A***** B******	DİYARBAKIR REKABET KURUMU CUMHURİYET FEN LİSESİ	EZGİLERİN İZİNDE: YÖRESEL TÜRKÜ HARİTASI	TÜRK DİLİ VE EDEBİYATI	Dijital Dönüşüm	MALATYA	BÖLGE BİRİNCİLİĞİ
406	1689B012432608	Y**** B*******	G**** Ç*****	MALATYA MUSTAFA İCLAL BAŞER ANADOLU LİSESİ	EDEBİYATIN ŞAMPİYONU SEN OL	TÜRK DİLİ VE EDEBİYATI	Dijital Oyun Tasarımı	MALATYA	BÖLGE İKİNCİLİĞİ
407	1689B012403264	M**** S*** B*****<br>Y**** K****	A********** Ç********	BİNGÖL BİNGÖL BİLİM VE SANAT MERKEZİ	FUZULİ'NİN ŞİİRLERİNDE YAPAY ZEKA İLE DUYGUSAL TON ANALİZİ: TÜRK EDEBİYATINDA BİR İNCELEME	TÜRK DİLİ VE EDEBİYATI	Yapay Zekâ	MALATYA	BÖLGE ÜÇÜNCÜLÜĞÜ
408	1689B012433288	M******* Y*** B********<br>A*** Ç****<br>H**** Ö***	A**** B******	BİNGÖL KARŞIYAKA ANADOLU LİSESİ<br>BİNGÖL TÜRKİYE BÜYÜK MİLLET MECLİSİ VAKFI BİNGÖL FEN LİSESİ	ADPROVIOT ACİL DURUM MÜDEHALE ROBOTU	YAZILIM	Robotik ve Kodlama	MALATYA	BÖLGE BİRİNCİLİĞİ
409	1689B012404587	U*** Z***	M****** G*****	MALATYA BATTALGAZİ ANADOLU LİSESİ	SU BEY	YAZILIM	Milli Teknoloji Hamlesi	MALATYA	BÖLGE İKİNCİLİĞİ
410	1689B012432942	M****** E*** K***<br>Y**** G**** Ş****	R**** Ş****	ADIYAMAN ALTINŞEHİR ANADOLU LİSESİ<br>ADIYAMAN ADIYAMAN FEN LİSESİ	YAPAY ZEKâ İLE BEYİN TÜMÖR TESPİTİ VE ERKEN TANI SİSTEMİ	YAZILIM	Yapay Zekâ	MALATYA	BÖLGE ÜÇÜNCÜLÜĞÜ
411	1689B012422712	E*** Y********<br>B***** K******<br>E*** Ç****	G****** T****	GİRESUN BULANCAK MESLEKİ VE TEKNİK ANADOLU LİSESİ	SANAL LABORATUVAR	BİYOLOJİ	Artırılmış, Sanal ve Karma Gerçeklik	SAMSUN	BÖLGE BİRİNCİLİĞİ
412	1689B012427720	R*** Ş****<br>Y***** A******* T****<br>S*** B******	G***** K*****	ORDU ÖZEL ORDU BAHÇEŞEHİR KOLEJİ FEN VE TEKNOLOJİ LİSESİ	CACO3 YAPILI BALIK OTOLİTLERİNİN ÇİNKO FOSFAT SİMANINA KATKISI: BİYOLOJİK DİŞ SİMANI	BİYOLOJİ	Atık Yönetimi ve Geri Dönüşüm	SAMSUN	BÖLGE BİRİNCİLİĞİ
413	1689B012401453	D*** A*****<br>D**** A*****	S**** Y***** U***	GİRESUN BULANCAK FEN LİSESİ<br>GİRESUN GİRESUN MİMAR SİNAN ANADOLU LİSESİ	PORTAKAL KABUKLARINDAN ELDE EDİLEN PEKTİN BAZLI, BACİLLUS SUBTİLİS İLE BİYOYAPISAL FONKSİYONU GÜÇLENDİRİLMİŞ BİYOYAPIŞTIRICI ÜRETİMİ (BİYOPEK-BS)	BİYOLOJİ	Malzeme Bilimi ve Nanoteknoloji	SAMSUN	BÖLGE İKİNCİLİĞİ
414	1689B012430818	C** M******	M***** K****	ORDU ÖZEL ORDU BAHÇEŞEHİR KOLEJİ FEN VE TEKNOLOJİ LİSESİ	KAĞIT TABANLI KOLORİMETRİK SENSÖR VE YARI İLETKEN SENSÖR İLE KANSER HIZLI ÖN TANISI	BİYOLOJİ	Biyomedikal Cihaz Teknolojileri	SAMSUN	BÖLGE ÜÇÜNCÜLÜĞÜ
415	1689B012415000	A**** A*** K******<br>T***** Y********	S********* Ö******	ORDU DR. M. HİLMİ GÜLER BİLİM VE SANAT MERKEZİ	UZAKTAN ALGILAMA TEKNİKLERİNDEN FAYDALANARAK DEPREMLERİN ÖNGÖRÜLMESİ FİKRİNİN DÜNYA VE TÜRKİYE'DEN ÖRNEKLERLE İNCELENMESİ	COĞRAFYA	Veri Madenciliği	SAMSUN	BÖLGE BİRİNCİLİĞİ
416	1689B012432783	R****** Ö****<br>A*** N** K****<br>R****** Ö****	K**** K****	GİRESUN GİRESUN FEN LİSESİ	TEKNOLOJİK BİR BAKIŞTAN EFSANELERİN TURİSTİK ÇEKİCİLİK ÜZERİNE ETKİSİNİN İNCELENMESİ: EFSADA-GİRESUN	COĞRAFYA	Kültürel Miras	SAMSUN	BÖLGE İKİNCİLİĞİ
417	1689B012428455	E**** İ*** T********<br>İ*** Y***** G****	N****** K*****	AMASYA ŞEHİT FERHAT ÜNELLİ BİLİM VE SANAT MERKEZİ	MANDALA UYGULAMALARINDA AMASYA ENDEMİK BİTKİLERİ: BİR DOĞAL MİRAS FARKINDALIK ÇALIŞMASI	COĞRAFYA	Doğal Miras ve Doğal Kaynaklar	SAMSUN	BÖLGE ÜÇÜNCÜLÜĞÜ
418	1689B012427875	D**** B****<br>E**** B**** T****<br>A*** K***	S*** S**** Ç*****	GİRESUN GİRESUN FEN LİSESİ	YILDIZLARIN İZİNDE	DEĞERLER EĞİTİMİ	Değerler Eğitimi	SAMSUN	BÖLGE BİRİNCİLİĞİ
419	1689B012434616	M****** B*****<br>E*** M**** G****	Ü*** P**** D**	SAMSUN ATAKUM ANADOLU İMAM HATİP LİSESİ	LİSAN-I NEZAKET	DEĞERLER EĞİTİMİ	Yaşamımızda İyilik, Nezaket ve Anlayış	SAMSUN	BÖLGE BİRİNCİLİĞİ
420	1689B012435250	R***** S****<br>S***** Ö******<br>Z***** S*** Ç****	M***** D*****	SAMSUN ATAKUM ANADOLU İMAM HATİP LİSESİ	LİSE ÖĞRENCİLERİNDE KARAKTER EĞİTİMİ PROGRAMI (KEP) RIZA HEYBESİ ÖRNEĞİ	DEĞERLER EĞİTİMİ	Değerler Eğitimi	SAMSUN	BÖLGE İKİNCİLİĞİ
421	1689B012415187	M*** C*****<br>S**** E*** N**	K***** D****	SAMSUN MİMAR SİNAN MESLEKİ VE TEKNİK ANADOLU LİSESİ	MEB E BAĞLI OKULLARDA SADECE MUSLUK VANALARININ KISILMASI YÖNTEMİNİN ÜLKEMİZDEKİ SU TASARRUFUNA ETKİSİNİN İNCELENMESİ	DEĞERLER EĞİTİMİ	Su Okuryazarlığı	SAMSUN	BÖLGE ÜÇÜNCÜLÜĞÜ
422	1689B012428232	İ*** D***** K****<br>Z***** B*******	A****** H********	ORDU ÖZEL ORDU BAHÇEŞEHİR KOLEJİ FEN VE TEKNOLOJİ LİSESİ	SİPİNACİA OLERACEA VE CİTRUS SİNENSİS KABUĞUNUN KARBON KUANTUM NOKTALARINDAN ORGANİK ESNEK GÜNEŞ PANELİ EKLENTİSİ	FİZİK	Yenilenebilir Enerji	SAMSUN	BÖLGE BİRİNCİLİĞİ
423	1689B012404700	E*** N** U***<br>Z***** K*****	Y**** Ş****	ÇORUM ALACA BİLİM VE SANAT MERKEZİ	BAYKUŞ KANADINDAN İLHAM ALARAK SESSİZ PERVANE TASARIMI	FİZİK	Havacılık ve Uzay Bilimleri	SAMSUN	BÖLGE İKİNCİLİĞİ
424	1689B012432251	E*** Y*******<br>B**** S***	İ****** P**********	SAMSUN SAMSUN ANADOLU LİSESİ	IŞIĞIN KIRILMA ÖZELLİĞİNDEN YARARLANARAK KIZARTMA YAĞLARININ KULLANILABİLİRLİĞİNİN BELİRLENMESİ	FİZİK	Gıda ve Gıda Arzı Güvenliği	SAMSUN	BÖLGE ÜÇÜNCÜLÜĞÜ
425	1689B012400005	N*** K****<br>B*** K****	E*** K****** U****	ORDU DR. M. HİLMİ GÜLER BİLİM VE SANAT MERKEZİ	ARONYA İNCİLERİ	KİMYA	Sağlıklı Beslenme	SAMSUN	BÖLGE BİRİNCİLİĞİ
426	1689B012423218	E*** E** T*****<br>E** C**** U*****	S********* Ö******	ORDU ÖZEL ORDU BAHÇEŞEHİR KOLEJİ FEN VE TEKNOLOJİ LİSESİ<br>ORDU DR. M. HİLMİ GÜLER BİLİM VE SANAT MERKEZİ	FARKLI YÖNTEMLERLE ELDE EDİLEN EKSTRAKTLARIN ASETİLKOLİNESTERAZ VE BUTİRİLKOLİNESTERAZ İNHİBİSYON ETKİNLİĞİNİN BELİRLENMESİ	KİMYA	Sağlıklı Beslenme	SAMSUN	BÖLGE İKİNCİLİĞİ
427	1689B012418470	A*** S**** Ö******<br>G****** B**** Ö*****<br>N******* K****	S***** T**	ORDU ÖZEL NAMIK ALTAŞ KOLEJİ FEN LİSESİ	KESME ÇİÇEKLERİN VAZO ÖMRÜNÜ UZATMAK İÇİN YENİLİKÇİ ÇÖZÜM: MELATONİN İÇEREN POLİKAPROLAKTON NANOFİBERLER	KİMYA	Malzeme Bilimi ve Nanoteknoloji	SAMSUN	BÖLGE ÜÇÜNCÜLÜĞÜ
428	1689B012436523	M****** Ç**** K*******<br>D**** K*******<br>H***** T******	N***** G***	ORDU FATSA FEN LİSESİ<br>GİRESUN BULANCAK FEN LİSESİ<br>ORDU FATİH ANADOLU LİSESİ	N BOYUTLU UZAYI N-1 UZAY İLE KESME KESİM NOKTA SAYISINI VEREN KAPALI FORMÜLÜ BULMA	MATEMATİK	STEAM (Fen, Teknoloji, Mühendislik, Sanat ve Matematik)	SAMSUN	BÖLGE BİRİNCİLİĞİ
429	1689B012414214	M******* K******* A*******<br>E*** K***<br>E**** E*** Y*****	E*** K****** G****	KASTAMONU KASTAMONU FEN LİSESİ	PARABOLOİD	MATEMATİK	Özgün Algoritma Tasarımı	SAMSUN	BÖLGE İKİNCİLİĞİ
430	1689B012428792	R***** P*****<br>A**** B**** D*****<br>F**** R*** İ******	E*** İ***	SAMSUN SAMSUN BİLİM VE SANAT MERKEZİ	TOPLAMLARI EŞİT İKİ SAYI GRUBUNUN PARÇALANIŞ SAYISI İLE İLİŞKİSİ	MATEMATİK	Özgün Algoritma Tasarımı	SAMSUN	BÖLGE ÜÇÜNCÜLÜĞÜ
431	1689B012427528	Z******* G**<br>M******* T**** D****<br>M***** A*** Ü***	M***** B*****	TOKAT ERBAA ANADOLU İMAM HATİP LİSESİ	BENİM OYUNUM BEN O'YUM	PSİKOLOJİ	Yaşamımızda İyilik, Nezaket ve Anlayış	SAMSUN	BÖLGE BİRİNCİLİĞİ

SIRA NO	BAŞVURU NO	ÖĞRENCİ ADI SOYADI	DANIŞMAN ADI SOYADI	OKULU	PROJENİN ADI	ANA ALANI	TEMATİK ALANI	BÖLGESİ	BÖLGE AŞAMASI SONUCU
432	1689B012418310	M***** E*** U***<br>S**** B****	A**** A*****	TOKAT YILMAZ KAYALAR FEN LİSESİ	OKUL RİSK HARİTALARININ YAPAY ZEKâ MODELLEMELERİ İLE ANALİZ EDİLMESİ	PSİKOLOJİ	Yapay Zekâ	SAMSUN	BÖLGE İKİNCİLİĞİ
433	1689B012408284	B****** G****	Ö**** Ç****** A****	GİRESUN GİRESUN BİLİM VE SANAT MERKEZİ	KİM DAHA YARATICI: YAPAY ZEKA MI İNSAN MI?	PSİKOLOJİ	Yapay Zekâ	SAMSUN	BÖLGE ÜÇÜNCÜLÜĞÜ
434	1689B012423426	Z***** B**<br>E** K**************	G***** Ş**** B*****	SAMSUN SAMSUN BİLİM VE SANAT MERKEZİ	LİSELİ ERGENLERDE AFET SONRASI YAŞAMIN ANLAMI VE ÖLÜM ALGISI.	SOSYOLOJİ	Doğal Afetler ve Afet Yönetimi	SAMSUN	BÖLGE BİRİNCİLİĞİ
435	1689B012426982	Ş**** B*****<br>M**** E******<br>E*** E**** Ç*****	H*** H**** A**	TOKAT YILMAZ KAYALAR FEN LİSESİ	EKOLOJİK AYAK İZİMİN FARKINDAYIM	SOSYOLOJİ	Küresel Isınma ve İklim Değişikliği	SAMSUN	BÖLGE BİRİNCİLİĞİ
436	1689B012411883	H****** A***** A*****<br>H**** N** B****<br>D**** S* M****	B***** S***	ÇORUM HASANPAŞA MESLEKİ VE TEKNİK ANADOLU LİSESİ	ŞİMDİ ALMA ZAMANI	SOSYOLOJİ	Finansal Okuryazarlık	SAMSUN	BÖLGE İKİNCİLİĞİ
437	1689B012407999	Z***** S**** Ö***<br>H**** O***	E**** Y*****	GİRESUN GİRESUN SOSYAL BİLİMLER LİSESİ	ERGENLERİN ALGILANAN EBEVEYN SOSYOTELİZM DÜZEYLERİ İLE AİLE İŞLEVLERİNDE YAŞANAN SORUNLAR ARASINDAKİ İLİŞKİNİN İNCELENMESİ	SOSYOLOJİ	Aile İçi İletişim	SAMSUN	BÖLGE ÜÇÜNCÜLÜĞÜ
438	1689B012430910	B**** B****	B***** K****	SAMSUN CANİK MESLEKİ VE TEKNİK ANADOLU LİSESİ	TÜRK TARİHİNİ SANAL GERÇEKLİKLE KEŞFET: 100 YILIN ÖYKÜSÜ	TARİH	Metaverse	SAMSUN	BÖLGE BİRİNCİLİĞİ
439	1689B012425992	D**** N**** Ü****<br>E***** Ç**********<br>Ş***** K****	N******* S***	GİRESUN GİRESUN FEN LİSESİ	42.ALAYDAN 42 İPLE TAMZARA DOKUMASI	TARİH	Kültürel Miras	SAMSUN	BÖLGE İKİNCİLİĞİ
440	1689B012432044	Z***** R********<br>B******* T********	M******* K**	KASTAMONU ABDURRAHMANPAŞA LİSESİ	TARİHİNİ BİL KAZAN	TARİH	Kültürel Miras	SAMSUN	BÖLGE ÜÇÜNCÜLÜĞÜ
441	1689B012406719	İ** T**** İ****<br>M******* E*** D******<br>M******* E*** D****	E**** S*****	ORDU YUSUF BAHRİ ANADOLU İMAM HATİP LİSESİ	ÇOK AMAÇLI ARAMA KURTARMA İHA'SI (ÇAKA)	TEKNOLOJİK TASARIM	Doğal Afetler ve Afet Yönetimi	SAMSUN	BÖLGE BİRİNCİLİĞİ
442	1689B012417787	Y**** E*** G*******<br>H**** H****** B****<br>M******* E*** D******	A**** A*****	TOKAT YILMAZ KAYALAR FEN LİSESİ	VAKUM ÖZELLİKLİ KOMBİ TASARIMI	TEKNOLOJİK TASARIM	Su Okuryazarlığı	SAMSUN	BÖLGE BİRİNCİLİĞİ
443	1689B012431283	A******* U*** G******<br>H****** E*	H**** Y******	TOKAT ZİLE MESLEKİ VE TEKNİK ANADOLU LİSESİ	GAZ PEDALINA DUYARLI FREN DESTEK SİSTEMİ	TEKNOLOJİK TASARIM	Akıllı Ulaşım Sistemleri	SAMSUN	BÖLGE İKİNCİLİĞİ
444	1689B012412727	İ** A*** Ö*****<br>A**** O*** K****<br>Y**** T*** Y****	H****** T*****	AMASYA TÜRK TELEKOM ANADOLU İMAM HATİP LİSESİ	DEVRİLEN YOLCU ARAÇLARINDA YARALANMALARA KARŞI KORUYUCU YASTIK SİSTEMİ	TEKNOLOJİK TASARIM	Akıllı Ulaşım Sistemleri	SAMSUN	BÖLGE ÜÇÜNCÜLÜĞÜ
445	1689B012414076	K**** N** S*****<br>K**** D*****<br>G**** E***	H****** E*****	SAMSUN MİTHATPAŞA ANADOLU LİSESİ	Z KUŞAĞININ E-DİLİ	TÜRK DİLİ VE EDEBİYATI	Medya Okuryazarlığı	SAMSUN	BÖLGE BİRİNCİLİĞİ
446	1689B012426998	N**** T**** T****<br>D**** A****<br>İ*** Ş*** Y*******	S*** S**** Ç*****	GİRESUN GİRESUN FEN LİSESİ	'YÜZÜKLERİN EFENDİSİ' NDEN 'HÜSN Ü AŞK'A' DİJİTAL DÜNYADA FANTASTİK BİR YOLCULUK	TÜRK DİLİ VE EDEBİYATI	Kültürel Miras	SAMSUN	BÖLGE BİRİNCİLİĞİ
447	1689B012408379	H********* S****	İ***** B****	SAMSUN NAMIK KEMAL ANADOLU LİSESİ	YERALTI EDEBİYATININ LİSE GENÇLİĞİNE BIRAKTIĞI ETKİ ÜZERİNE BİR FARKINDALIK DEĞERLENDİRMESİ	TÜRK DİLİ VE EDEBİYATI	Dil ve Edebiyat	SAMSUN	BÖLGE İKİNCİLİĞİ
448	1689B012434283	A**** O*** S****<br>M****** S**** S***	Ö*** F**** K****	KASTAMONU KASTAMONU FEN LİSESİ	İMLATÜRK: TÜRKÇE İMLA ÖĞRETİCİSİ	TÜRK DİLİ VE EDEBİYATI	Oyun ve Oyunlaştırma	SAMSUN	BÖLGE ÜÇÜNCÜLÜĞÜ
449	1689B012438267	B**** E******<br>B******* A*** Ö******	A*** Ü***	SAMSUN AZİZ ATİK FEN LİSESİ	CARDGUARD: ONLİNE ALIŞVERİŞLERDE YÜKSEK GÜVENLİK - KART ŞİFRELEME VE YAPAY ZEKâLI SİTE KONTROLÜ	YAZILIM	Siber Güvenlik	SAMSUN	BÖLGE BİRİNCİLİĞİ
450	1689B012403580	S**** E*** Ö****	H**** A****	TOKAT ULUSLARARASI TOKAT ANADOLU İMAM HATİP LİSESİ	STEGONTROPY - BLOK KARMAŞASI TABANLI YENİ BİR VERİ GİZLEME TEKNİĞİ	YAZILIM	Özgün Algoritma Tasarımı	SAMSUN	BÖLGE İKİNCİLİĞİ
451	1689B012429040	M**** İ*******	S***** U***	SAMSUN SAMSUN GARİP ZEYCAN YILDIRIM FEN LİSESİ	YAPAY ZEKA'DA U-NET VE CAPSNET'İN HİBRİT KULLANIMI İLE AKCİĞER TARAMALARINDA NODÜL TESPİTİ VE SEGMENTASYONU	YAZILIM	Yapay Zekâ	SAMSUN	BÖLGE ÜÇÜNCÜLÜĞÜ
452	1689B012408103	N**** K*******<br>Y***** Y**** B******	T**** A****	VAN YUSUF GÖKÇENAY BİLİM VE SANAT MERKEZİ	BİYOLOJİ EĞİTİMİNDE METAVERSE ORTAMININ LİSE ÖĞRENCİLERİ İÇİN KULLANIMI: BİYOVERSE OYUNU	BİYOLOJİ	Dijital Oyun Tasarımı	VAN	BÖLGE BİRİNCİLİĞİ
453	1689B012428283	E****** Y****<br>M**** A***	E*** A*****	BATMAN TÜRK TELEKOM ANADOLU LİSESİ	DÖKÜLMÜŞ YAPRAKLARDAN BİYOÇÖZÜNÜRLÜĞÜ YÜKSEK BİYOPLASTİK ÜRETİMİ	BİYOLOJİ	Atık Yönetimi ve Geri Dönüşüm	VAN	BÖLGE BİRİNCİLİĞİ
454	1689B012404725	E** K*********<br>R**** K***	G**** K*******	AĞRI TAŞLIÇAY ANADOLU İMAM HATİP LİSESİ	KIRMIZI LAHANA BİTKİSİ İLE RENKLENDİRİLMİŞ ARPA BİTKİSİNDEN BİYOPLASTİK YAPIMI	BİYOLOJİ	Malzeme Bilimi ve Nanoteknoloji	VAN	BÖLGE İKİNCİLİĞİ
455	1689B012429605	Ş**** Z**** K*****<br>E**** S*** K*****	H***** U***	BİTLİS BİTLİS AHMET EREN BİLİM VE SANAT MERKEZİ	PLASTİK ÇAĞIN GÖRÜNMEYEN YÜZÜ: TEK KULLANIMLIK KARTON BARDAKLARDAKİ MİKROPLASTİKLER	BİYOLOJİ	Gıda ve Gıda Arzı Güvenliği	VAN	BÖLGE ÜÇÜNCÜLÜĞÜ
456	1689B012422035	C*** Ü***	C**** İ***	ŞIRNAK ULUDERE ÇOK PROGRAMLI ANADOLU LİSESİ	LİSE ÖĞRENCİLERİNİN "SU" KAVRAMINA İLİŞKİN METAFORİK ALGILARI (ULUDERE ÖRNEĞİ)	COĞRAFYA	Su Okuryazarlığı	VAN	BÖLGE BİRİNCİLİĞİ
457	1689B012430236	M******* İ**** K***	M**** G****	AĞRI AĞRI HÜSEYİN CELAL YARDIMCI FEN LİSESİ	VOLKANİK TOPRAKLARIN TARIM ALANLARINA TAŞINMASININ YÜKSEK VERİMLİ ÜRÜN ALIMINA ETKİSİ, AĞRI İLİ ÖRNEĞİ	COĞRAFYA	Doğal Miras ve Doğal Kaynaklar	VAN	BÖLGE BİRİNCİLİĞİ
458	1689B012419951	E**** Ş***** B*****<br>R****** Y*****	S**** Y****	SİİRT MARMARA ANADOLU LİSESİ	AFET YÖNETİMİ VE DEPREM	COĞRAFYA	Doğal Afetler ve Afet Yönetimi	VAN	BÖLGE İKİNCİLİĞİ
459	1689B012403417	N******* Y*******<br>S**** Y****	V*** Y*****	AĞRI ŞERİFE BACI ANADOLU LİSESİ	ORTAÖĞRETİM COĞRAFYA DERSLERİNDE CBS'NİN YERİ, ÖNEMİ VE GEREKLİLİĞİNE İLİŞKİN ÖĞRENCİ GÖRÜŞLERİ	COĞRAFYA	Medya Okuryazarlığı	VAN	BÖLGE ÜÇÜNCÜLÜĞÜ
460	1689B012419938	Y***** Ç****<br>B******* İ******<br>M***** D***	Y**** Ç****	BATMAN MEVLANA ANADOLU LİSESİ	FABL VE EĞİTSEL OYUN ETKİNLİKLERİNİN DEĞERLER EĞİTİMİ ÜZERİNE ETKİSİ: YARDIMLAŞMA VE SORUMLULUK	DEĞERLER EĞİTİMİ	Değerler Eğitimi	VAN	BÖLGE BİRİNCİLİĞİ
461	1689B012423618	B***** D**** Ş*****	M***** T***	VAN YUSUF GÖKÇENAY BİLİM VE SANAT MERKEZİ	YENİ DÜNYA BENİM DEĞERLERİM (YARDIM MELEĞİ)	DEĞERLER EĞİTİMİ	Dijital Oyun Tasarımı	VAN	BÖLGE BİRİNCİLİĞİ
462	1689B012418988	H****** Ç****<br>A*** N*** K*********	Ö**** Ö***	SİİRT SİİRT SOSYAL BİLİMLER LİSESİ	İLERİ DÖNÜŞEBİLİYORUM FARKINDA MISIN?	DEĞERLER EĞİTİMİ	Ekolojik Denge	VAN	BÖLGE İKİNCİLİĞİ

SIRA NO	BAŞVURU NO	ÖĞRENCİ ADI SOYADI	DANIŞMAN ADI SOYADI	OKULU	PROJENİN ADI	ANA ALANI	TEMATİK ALANI	BÖLGESİ	BÖLGE AŞAMASI SONUCU
463	1689B012422195	H**** T****<br>M***** B****	N****** E******	BATMAN TÜRK TELEKOM ANADOLU LİSESİ	ANLAYIŞ UYUM HUZUR	DEĞERLER EĞİTİMİ	Aile İçi İletişim	VAN	BÖLGE ÜÇÜNCÜLÜĞÜ
464	1689B012403944	B****** S***<br>M****** B****	F*** T*****	BATMAN MEHMET AKİF ERSOY ANADOLU LİSESİ<br>BATMAN BATMAN MESLEKİ VE TEKNİK ANADOLU LİSESİ	MİKRO KONTROL KART İLE HARİCİ FAZ NÖTR DURUMLARINDA KAÇAK ELEKTRİK TESPİTİ	FİZİK	Sorumlu Üretim ve Tüketim	VAN	BÖLGE BİRİNCİLİĞİ
465	1689B012430713	Z***** Ö****	S**** T****	HAKKARİ HAKKARİ ANADOLU LİSESİ	RÜZGARSIZ RÜZGAR ENERJİSİ	FİZİK	Yenilenebilir Enerji	VAN	BÖLGE İKİNCİLİĞİ
466	1689B012411379	Y**** Y****	Y**** Ö******	MUŞ MUŞ MESLEKİ VE TEKNİK ANADOLU LİSESİ	ATIKLAR, YENİDEN KULLANIM İLE FİZİK DENEYLERİYLE BULUŞUYOR	FİZİK	Sürdürülebilir Şehirler ve Toplumlar	VAN	BÖLGE ÜÇÜNCÜLÜĞÜ
467	1689B012411248	B**** L*****<br>A******* E*** O***	M***** A****	BATMAN BATMAN BİLİM VE SANAT MERKEZİ	LİPOPEPTİT ANTİBİYOTİK İLAÇ OLAN DAPTOMİSİN'İN ELEKTROKİMYASAL DAVRANIŞLARI VE MULTİDİSİPLİNER BİR YAKLAŞIM İLE DNA ETKİLEŞİMLERİNİN FARKLI TEKNİKLER İLE İNCELENMESİ	KİMYA	Halk Sağlığı ve Koruyucu Sağlık Hizmetleri	VAN	BÖLGE BİRİNCİLİĞİ
468	1689B012419888	E**** K*******<br>A**** K*******	S**** A******	ŞIRNAK ŞÜKRÜ GELİŞ FEN LİSESİ	CRATAEGUS L. AZAROLUS VAR. AZAROLUS VE C. MONOGYNA TAKSONLARINA AİT MEYVELERDE ELEMENTEL ANALİZ VE ANTİOKSİDAN AKTİVİTELERİNİNBELİRLENMESİ	KİMYA	Halk Sağlığı ve Koruyucu Sağlık Hizmetleri	VAN	BÖLGE İKİNCİLİĞİ
469	1689B012413493	M******* K**** C******<br>Y**** K******	F**** Y**** G*****	VAN ÖZEL DÜŞÜNÜR KOLEJİ FEN LİSESİ	OPUNTİA(KAYNANADİLİ) KATKILI DOĞAL DİŞ MACUNU	KİMYA	Halk Sağlığı ve Koruyucu Sağlık Hizmetleri	VAN	BÖLGE ÜÇÜNCÜLÜĞÜ
470	1689B012425967	M******* Y**** Y*****	Ş**** K****	VAN ARİF NİHAT ASYA ANADOLU LİSESİ	İKİNCİ DERECEDEN KUVVET ARITIŞININ GENELLEŞTİRİLMESİ	MATEMATİK	Özgün Algoritma Tasarımı	VAN	BÖLGE BİRİNCİLİĞİ
471	1689B012431957	R****** A***** U******<br>M**** A***<br>H***** F**** A****	İ**** U***	BATMAN ABDÜLHAMİD HAN ANADOLU LİSESİ	S FORMÜLÜ	MATEMATİK	STEAM (Fen, Teknoloji, Mühendislik, Sanat ve Matematik)	VAN	BÖLGE İKİNCİLİĞİ
472	1689B012423103	S**** B*****	S****** S****	MUŞ ŞEHİT DAVUT KARAÇAM KIZ ANADOLU İMAM HATİP LİSESİ	FİBONACCİ-KRİPTO24	MATEMATİK	Özgün Algoritma Tasarımı	VAN	BÖLGE ÜÇÜNCÜLÜĞÜ
473	1689B012429204	A*** T*****<br>N****** A****	R**** Ş***** Ş*****	SİİRT SİİRT İBRAHİM HAKKI KIZ ANADOLU İMAM HATİP LİSESİ	EĞİTİM ORTAMINDA GÖÇMENLERE SUNULAN REHBERLİK VE PSİKOLOJİK DANIŞMANLIK HİZMETLERİNİN KARŞILAŞTIRILMASI: ALMANYA VE TÜRKİYE ÖRNEĞİ	PSİKOLOJİ	Göç ve Uyum	VAN	BÖLGE BİRİNCİLİĞİ
474	1689B012427745	İ*** D****<br>G**** T****	E*** A*****	BATMAN TÜRK TELEKOM ANADOLU LİSESİ	DİSLEKSİ DOSTU, YAPAY ZEKA DESTEKLİ DİSLEKSİNİN ERKEN TEŞHİSİ AMAÇLI WEB TABANLI UYGULAMA	PSİKOLOJİ	Yapay Zekâ	VAN	BÖLGE İKİNCİLİĞİ
475	1689B012410465	Y**** A******<br>M***** D****<br>İ**** K******	M***** B*****	BİTLİS SADULLAH GENCER ANADOLU LİSESİ	EKO ANKSİYETE YAŞIYOR OLABİLİRSİN! (İKLİM DEĞİŞİKLİĞİNİN PSİKOLOJİK SAĞLIK ÜZERİNDEKİ ETKİLERİ VE ÇÖZÜM ÖNERİLERİ - AHLAT ÖRNEĞİ)	PSİKOLOJİ	Küresel Isınma ve İklim Değişikliği	VAN	BÖLGE ÜÇÜNCÜLÜĞÜ
476	1689B012430773	D**** G**** İ****<br>S***** A****	Ö**** Ö***	SİİRT SİİRT SOSYAL BİLİMLER LİSESİ	SİİRT'İN SESSİZ ŞAHİTLERİ: CAS EVLERİ	SOSYOLOJİ	Kültürel Miras	VAN	BÖLGE BİRİNCİLİĞİ
477	1689B012434827	H**** V****<br>S****** A***	A**** T***	ŞIRNAK ŞEHİT KENAN YILDIZ ANADOLU LİSESİ	"AŞKO, KUŞKO" DİLİNİN TÜRKÇE'YE ETKİSİ	SOSYOLOJİ	Türk Dili ve Lehçeleri	VAN	BÖLGE İKİNCİLİĞİ
478	1689B012428393	Z**** G******<br>D**** S*** D****<br>R**** S****	O**** Ç******* Y*******	VAN İPEKYOLU ANADOLU LİSESİ	KENTSEL HAFIZADA İŞ YERİ TABELALARININ DİLBİLİMSEL AÇIDAN İNCELENMESİ: VAN İLİ ÖRNEĞİ	SOSYOLOJİ	Sürdürülebilir Şehirler ve Toplumlar	VAN	BÖLGE ÜÇÜNCÜLÜĞÜ
479	1689B012412750	M**** N** A****<br>H****** D****<br>Y***** K****	G**** N** A******	AĞRI TUTAK ANADOLU İMAM HATİP LİSESİ	GEÇMİŞTEN GELECEĞİMİZE TÜRK VE İSLAM SEMBOLLERİNİN SENTEZİYLE YENİ TASARIM ÖNERİLERİ	TARİH	Kültürel Miras	VAN	BÖLGE BİRİNCİLİĞİ
480	1689B012419823	Y**** E*** S****<br>A**** N**** R*****<br>M****** İ**** A****	Z***** A****	BATMAN AHMEDİ HANİ ANADOLU İMAM HATİP LİSESİ	YOK OLMADAN	TARİH	Kültürel Miras	VAN	BÖLGE İKİNCİLİĞİ
481	1689B012431183	B**** Ç****<br>M******* T**	D**** B****	MUŞ MUŞ MESLEKİ VE TEKNİK ANADOLU LİSESİ	UYGARLIKLARIN UNUTULAN AYAK İZLERİ	TARİH	Kültürel Miras	VAN	BÖLGE ÜÇÜNCÜLÜĞÜ
482	1689B012430661	M***** E*** S****<br>E**** A****	F**** G*****	BATMAN BATMAN BİLİM VE SANAT MERKEZİ	STAND-BY KONUMUNDAN KAYNAKLANAN ELEKTRİK İSRAFINI ENGELLEMEK AMACIYLA TASARRUF PRİZİNİN TASARLANMASI.	TEKNOLOJİK TASARIM	STEAM (Fen, Teknoloji, Mühendislik, Sanat ve Matematik)	VAN	BÖLGE BİRİNCİLİĞİ
483	1689B012436774	M***** G****<br>D**** A***<br>Ö**** Y****	M**** S****	BATMAN BATMAN ANADOLU LİSESİ<br>BATMAN BATMAN FEN LİSESİ	LİSE ÖĞRENCİLERİNİN ROBOTİK KODLAMA EĞİTİMİNE DÖNÜK UZAKTAN KONTROLLÜ ÇOK İŞLEVLİ İNSANSI ROBOT TASARIMI	TEKNOLOJİK TASARIM	Robotik ve Kodlama	VAN	BÖLGE BİRİNCİLİĞİ
484	1689B012431725	H**** T**	E*** A*****	BATMAN TÜRK TELEKOM ANADOLU LİSESİ	ACİL KABLOSUZ LOKAL HABERLEŞME ÇAĞRI SİSTEMİ VE GÖÇÜK ALTINDA KALAN CANLILARIN KONUM TESPİTİ	TEKNOLOJİK TASARIM	Doğal Afetler ve Afet Yönetimi	VAN	BÖLGE İKİNCİLİĞİ
485	1689B012411902	A********* G****<br>A**** E*** B*********	M**** Z******	BATMAN MALABADİ MESLEKİ VE TEKNİK ANADOLU LİSESİ	ERKEN UYARI SİSTEMLERİYLE SEL VE HEYELAN TEHLİKELERİNE KARŞI KORUNMA	TEKNOLOJİK TASARIM	Doğal Afetler ve Afet Yönetimi	VAN	BÖLGE ÜÇÜNCÜLÜĞÜ
486	1689B012425379	Z**** S****<br>P****** K****<br>H**** G******	E**** A****	BİTLİS AHLAT SELÇUKLU ANADOLU LİSESİ	KONUŞMA VE DİNLEME SINAVLARININ EĞİTİM PAYDAŞLARINDAKİ YANSIMALARI (LİSE ÖRNEĞİ)	TÜRK DİLİ VE EDEBİYATI	Dil ve Edebiyat	VAN	BÖLGE BİRİNCİLİĞİ
487	1689B012430568	N****** K****<br>A****** Y*****	Z***** Ç***** G*****	BATMAN TÜRK TELEKOM ANADOLU LİSESİ	EĞİTİZM	TÜRK DİLİ VE EDEBİYATI	Dil ve Edebiyat	VAN	BÖLGE BİRİNCİLİĞİ
488	1689B012427017	H**** Ç****<br>E**** S****<br>G***** G*******	T*** Ö****	BATMAN GÜLTEPE ANADOLU LİSESİ	ENGELLİ BİREYLER İÇİN SANATSAL BİR ÇALIŞMA	TÜRK DİLİ VE EDEBİYATI	Dil ve Edebiyat	VAN	BÖLGE İKİNCİLİĞİ
489	1689B012403816	M***** U***<br>H***** Ş****<br>B**** Ç*****	H***** B**** D****	SİİRT SİİRT İBRAHİM HAKKI KIZ ANADOLU İMAM HATİP LİSESİ	İNSANLIĞIN İÇ SESİ: DERLEM TEMELLİ BİR KÜLTÜREL ANAHTAR SÖZCÜK ÇALIŞMASI	TÜRK DİLİ VE EDEBİYATI	Dil ve Edebiyat	VAN	BÖLGE ÜÇÜNCÜLÜĞÜ
490	1689B012416668	E*** G**** Y*****<br>E*** A** T**********	E**** T****	VAN EDREMİT BİLİM VE SANAT MERKEZİ	ÖĞRENMEYE SAĞLIKLI BİR BAŞLANGIÇ: SINIF HAVA KALİTESİ	YAZILIM	Robotik ve Kodlama	VAN	BÖLGE BİRİNCİLİĞİ
491	1689B012401866	R****** A****<br>Y**** G****	E****** O**** R***	VAN MEHMET ERDEMOĞLU MESLEKİ VE TEKNİK ANADOLU LİSESİ	ÇOKLU HASTALIK TAHMİN SİSTEMİ	YAZILIM	Veri Madenciliği	VAN	BÖLGE İKİNCİLİĞİ
492	1689B012416245	Ş**** T***	M***** A***	BATMAN TÜRK TELEKOM ANADOLU LİSESİ	SINAVLARA HAZIRLIKTA, YAPAY ZEKâ DESTEKLİ YANLIŞ DOKTORU	YAZILIM	Uzaktan Eğitim	VAN	BÖLGE ÜÇÜNCÜLÜĞÜ
